# Where do we stand on fMRI in awake mice?

**DOI:** 10.1093/cercor/bhad478

**Published:** 2023-12-13

**Authors:** Francesca Mandino, Stella Vujic, Joanes Grandjean, Evelyn M R Lake

**Affiliations:** Department of Radiology and Biomedical Imaging, Yale School of Medicine, New Haven, CT 06520, United States; Department of Computer Science, Yale University, New Haven, CT 06520, United States; Donders Institute for Brain, Behaviour, and Cognition, Radboud University, Nijmegen, The Netherlands; Department for Medical Imaging, Radboud University Medical Center, Nijmegen, The Netherlands; Department of Radiology and Biomedical Imaging, Yale School of Medicine, New Haven, CT 06520, United States; Department of Biomedical Engineering, Yale University, New Haven, CT 06520, United States

**Keywords:** rodent, functional magnetic resonance imaging, fMRI, awake, unanesthetized, anesthesia

## Abstract

Imaging awake animals is quickly gaining traction in neuroscience as it offers a means to eliminate the confounding effects of anesthesia, difficulties of inter-species translation (when humans are typically imaged while awake), and the inability to investigate the full range of brain and behavioral states in unconscious animals. In this systematic review, we focus on the development of awake mouse blood oxygen level dependent functional magnetic resonance imaging (fMRI). Mice are widely used in research due to their fast-breeding cycle, genetic malleability, and low cost. Functional MRI yields whole-brain coverage and can be performed on both humans and animal models making it an ideal modality for comparing study findings across species. We provide an analysis of 30 articles (years 2011–2022) identified through a systematic literature search. Our conclusions include that head-posts are favorable, acclimation training for 10–14 d is likely ample under certain conditions, stress has been poorly characterized, and more standardization is needed to accelerate progress. For context, an overview of awake rat fMRI studies is also included. We make recommendations that will benefit a wide range of neuroscience applications.

## Introduction

Historically, anesthesia has been a mainstay in neuroimaging of animal model species because it reduces subject motion—which corrupts data—and animal stress. Yet, (i) anesthesia fundamentally alters brain activity in ways that can obscure or confound study outcome measures (Gozzi et al. 2008; Barttfeld et al. 2015; Rungta et al. 2017; Slupe and Kirsch 2018), (ii) when an animal is anesthetized, it is not possible to investigate the full range of brain and behavioral states, and (iii) most human neuroimaging data are acquired from awake subjects. To ease translation across species, data acquired from awake animals may reduce some of the complexities involved (although many challenging aspects remain, e.g. differences in anatomy and brain function). This recognized need for neuroimaging data acquired from awake animals has prompted a concerted effort toward these implementations (see select reviews, Grinvald and Petersen 2015; Tran and Gordon 2015; Yu et al. 2015; Gao et al. 2017; Petersen 2017; Miranda et al. 2021; Ferris 2022; Durand et al. 2023).

There is a wide array of neuroimaging modalities available for interrogating brain function across species. Adapting experimental protocols to accommodate awake animals poses different challenges for different modalities and species. Functional magnetic resonance imaging (fMRI) is among the more difficult when performing awake imaging because of the significant acoustic noise (>120 dB), intolerance of head *and* body motion, limited working-space, and high magnetic field (which means metal components cannot be used). This means that the development of awake fMRI protocols is less widespread than awake protocols used in other fields (e.g. optical imaging). Despite these challenges, fMRI can be performed in both human subjects and animal models, making it an ideal candidate for translational research.

This is a review of published scientific articles on awake mouse fMRI identified through a systematic literature search. We choose to focus on mice as they are a widely used species in neuroimaging research due to their small size, low cost, fast breeding cycle, and genetic malleability. For context, we include an overview of the awake rat fMRI literature as these approaches predate, and have inspired, much of the work done in mice. We dissect strategies used to collect data, including animal immobilization, acclimation training, evaluation of stress, as well as image acquisition, data processing, and outcome metrics. Our goal is to ascertain which methods have been effective in reducing motion, animals and data excluded, and animal stress. While an analysis of the imaging data itself is beyond the scope of this review, we provide recommendations for best experimental practices and reporting. We aim to ease cross-study comparisons, facilitate standardization, and accelerate progress for a wide range of neuroscience applications. For clarity, the text roughly follows the sequential steps of an awake fMRI experiment.

### Systematic review: method and results

**Fig. 1:**
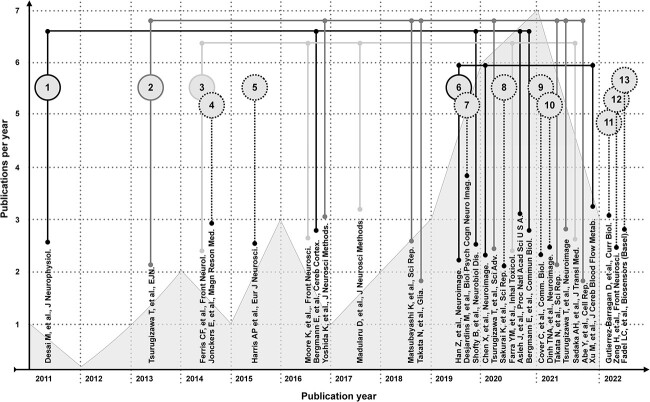
Taxonomy of articles identified in our systematic review of the awake mouse fMRI literature. Articles are listed in chronological order. The number of articles published per year is indicated by a gray histogram background. Articles which share co-authors, and by extension an experimental approach, are linked by lines. Groups which have contributed one article to the literature are indicated by numbers with dashed outlines. The seminal article from each independent group is numbered. A version of this figure with color-coding is included in the supplemental material (Fig. S1).

We identify a pool of articles following recent guidelines for systematic literature review (Atkinson and Cipriani 2018; Bramer et al. 2018). Our scope includes articles where blood-oxygen-level dependent (BOLD) fMRI data are collected from awake mice. Three databases (BioRxiv, ScienceDirect, and PubMed) are searched using the query statements in Table S1. Of the ~300 articles identified, 30 are within our scope. Articles beyond our scope discuss awake mouse fMRI but do not include these data. The high frequency with which these experiments are discussed attests to the impact studies that do collect these data are having on the field. The 30 articles appear in a wide range of neuroimaging journals which reflects the broad reach of this topic (complete list in Table S2). A taxonomy of the articles is illustrated in Fig. 1. Articles are shown in chronological order with the first publication appearing in 2011 (Desai et al. 2011). We note an increase in the number of publications per year and the number of labs performing these experiments. At the end of 2022, there were 13 groups worldwide that had published studies with awake mouse fMRI data.

### Overview of the data

A summary of the data is given in Table 1. Most studies (20/30) are performed on wild-type (WT) C57BL/6 mice. 10/30 studies examine models of human disorders/diseases/injuries or take advantage of the manipulatable mouse genome through optogenetic or gene knock-out applications (Ferris et al. 2014; Moore et al. 2016; Matsubayashi et al. 2018; Takata et al. 2018; Shofty et al. 2019; Asleh et al. 2020; Sakurai et al. 2020; Tsurugizawa et al. 2020; Abe et al. 2021). 24/30 studies give biological sex information. Among these, 22 report using males (total of 373), and two report using females (total of 23). An average/median (± standard deviation, SD) of 18 ± 9 mice is included per study. Age at imaging is estimated wherever possible. “Ns” are for animals that undergo awake fMRI (animals that only undergo other experiments are not counted).

**Table 1:** Mice studied using awake fMRI.

Study (Chronological)	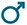	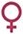	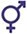	Age	WT	Other	Share
Desai 2011	Unk^*^	Unk^*^	5	Unk	C57BL/6 J	-	✕
Tsurugizawa 2013	18	0	18	6–7 w	C57BL	-	✕
Ferris 2014	Unk^*^	Unk^*^	29	12 m	C57BL/6 J	zQ175^+/−^ (*n* = 7) & zQ175^+/+^ (*n* = 10)	✕
Jonckers 2014	5	0	5	13 w	C57BL/6	-	✕
Harris 2015	20	0	20	14 w	C57BL/6	-	✕
Moore 2016	29	0	29	13 w	-	Oprm1^−/−^ (*n* = 19) & Oprm1^+/+^ (*n* = 10)	✕
Bergmann 2016	12	0	12	12–14 w	C57BL/6 J	-	✕
Yoshida 2016	9	0	9	>8 w	C57BL/6	-	✕
Madularu 2017	33	0	33	Adult	C57BL/6	-	✕
^a^ Matsubayashi 2018	0	9	9	>8 w	C57BL/6 J	-	✕
Takata 2018	Unk^*^	Unk^*^	21	8–12 w	-	Chrm4-tTA:tetO-ChR2(C128S) (*n* = 12) & Mlc1-tTA::tetO-ChR2(C128S)-EYFP (*n* = 9)	✕
Han 2019	8	0	8	10–12 w	C57BL/6	-	✕
Desjardins 2019	Unk^*^	Unk^*^	16	>8 w	C57BL/6 J	VGAT-ChR2(H134R)-EYFP (N = 9), Emx1-Cre/Ai32 (*n* = 5)	✕
Shofty 2019	22	0	22	12–14 w	C57BL/6 J	Nf1^+/−^ (*n* = 11)	✕
Chen 2020	40	0	40	7–10 w	C57BL/6	-	✕
Tsurugizawa 2020	25	0	25	10–17 w	C57BL/6 J	15q dup (*n* = 10)	✕
Sakurai 2020	6	0	6	12 m	C57BL/6	B6C3-Tg (APPswe/PSEN1dE9) (*n* = 3)	✕
Farra 2020	0	0	24	9–13 w	C57BL/6	-	✕
Asleh 2020	Unk^*^	Unk^*^	18	Unk	C57BL/6 J	Plp-Nf1^+/−^ (*n* = 11)	✓
Bergmann 2020	19	0	19	10–13 w	B6129PF/J1	-	✓
Cover 2021	15	0	15	12 w	C57BL/6 J	-	✓
Dinh 2021	7	0	7	11 w	C57BL/6	-	✕
Takata 2021	7	0	7	Adult	C57BL/6 J	-	✕
Tsurugizawa 2021 ^b^	27	0	27	10–15 w	C576J/BL		✕
Sadaka 2021	26	0	26	15–18 w	C57BL/J6	-	✕
Abe 2021	Unk^*^	Unk^*^	17	8–12 w	-	PV-ChR2 (*n* = 8) & PV-PAC (*n* = 9)	✕
Xu 2021	15	0	15	7–9 w	C57BL/6	-	✕
Gutierrez-Barragan 2022	10	0	10	<6 m	C57BL/6 J	-	✓
Zeng 2022	6	0	6	18–23 w	C57BL/6	-	✕
Fadel 2022	14	14	28	6–8 w	C57BL/6	-	✕
Total number of mice	373	23	526				69

Studies with WT and “other”: number of WT mice make up the difference between those listed under “other” and the total (e.g. Ferris et al. total *n* = 29, zQ175^+/−^ (*n* = 7) and zQ175^+/+^ (*n* = 10), thus *n* = 12 WT were also imaged).

^a^Matsubayashi—All mice had spinal cord injuries.

^b^Unk (unknown)—both sexes were included (breakdown not specified), week (w), month (m).

### Immobilization

**Fig. 2:**
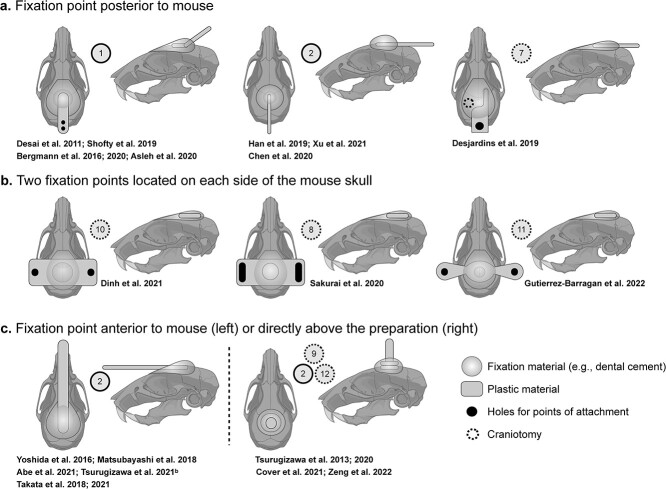
Surgically implanted head-posts. Head-posts used for immobilization during awake mouse fMRI fall into four categories based on the location of the fixation point(s): Posterior to the skull (a), on both the right and left side of the skull (b), anterior to the skull (c, left), and directly above the skull (c, right). Cartoon drawings of the preparations are shown in two views (from above, and the left side). The articles that implement each preparation are listed below the images. Every preparation uses a fixation material (e.g. dental cement) to attach a plastic piece (custom-made) to the bone. Many groups also etch, scratch, or thin the bone to obtain a more robust attachment. A version of this figure with color-coding is included in the supplemental material (Fig. S2).

In anesthetized experiments, the head is usually immobilized using ear-bars and an incisor-bar. These three points of contact reduce head motion, while anesthesia and the use of paralytic agents minimize body movements. In awake experiments, two approaches to immobilizing the head have emerged: (i) head-cradling devices that do not require surgery, and (ii) surgically implanting a head-post (Fig. 2). Most (22/30) studies in awake mice opt for a surgically implanted head-post. Body-restraint systems vary across studies (Table S3).

#### Head restraint

Groups of authors (Fig. 1) tend to use the same approaches which makes disentangling successful and unsuccessful strategies difficult (e.g. was it a short acclimation protocol or the decision not to use a head-post that contributed to high subject motion?). However, of studies that do not use a head-post (8/30), 3/8 do not report any outcome measures of motion (Farra et al. 2020; Moore et al. 2016; Jonckers et al. 2014), 3/8 report a higher-than-average number of excluded animals or scans based on motion or failure to habituate (Harris et al. 2015; Madularu et al. 2017a; Sadaka et al. 2021), and 1/8 reports high variance in outcome measures of motion (Fadel et al. 2022; “*Data processing approaches*”). The remaining study, by Ferris et al., is an exception and reports extremely low motion (Ferris et al. 2014) (potentially influenced by their long TR (repetition time)—6 s—and short scan times: 5–7 min). Of studies that report measures of animal stress (3/8) (Jonckers et al. 2014; Harris et al. 2015; Madularu et al. 2017a; “*Animal stress*”), there is no apparent difference between animals with and without a surgically implanted head-post. However, stress is an underdeveloped measure in awake rodent fMRI, and the studies that do not use head-posts predominantly come from one group (5/8) that uses a relatively short acclimation protocol of ~ 4 d, (“*Acclimation*”). Overall, we conclude that surgically implanted head-posts (if compatible with the study design) are favorable for awake mouse fMRI. In addition to reducing head motion, they circumvent the need to expose animals to anesthesia for the purpose of mounting, or unmounting, the animal into and out of the set-up (Desai et al. 2011; Yoshida et al. 2016; Matsubayashi et al. 2018; Takata et al. 2018; Han et al. 2019; Chen et al. 2020; Cover et al. 2021; Gutierrez-Barragan et al. 2022; Zeng et al. 2022) and help to reduce susceptibility artifacts if well executed.

Based on how we grouped head-posts (by fixation point, Fig. 2), we do not observe any indication that one approach outperforms any other at reducing stress (“*Animal stress*”) or motion (“*Data processing approaches*”). Common elements include a large footprint on the skull (combined with scoring or etching) to help reduce the likelihood of detachment. Notably, in experiments on mice, detachments are uncommon and have only been reported once (Zeng et al. 2022) (and may have occurred because of the long acclimation protocol—28 d—used in this study, “*Acclimation*”). With little to no modification, each head-post design is compatible with complementary multimodal experiments that require optical or physical access to cortical or deeper brain structures. Two designs: anterior and posterior fixation points (Fig. 2a, middle, 2c, left), are compatible with the Bruker CryoProbe (Yoshida et al. 2016; Matsubayashi et al. 2018; Takata et al. 2018, 2021; Han et al. 2019; Chen et al. 2020; Abe et al. 2021; Tsurugizawa and Yoshimaru 2021; Xu et al. 2021) which improves the signal to noise ratio (SNR) of fMRI and BOLD sensitivity (Ratering et al. 2008; Baltes et al. 2011; Niendorf et al. 2015). There is little to no variation in surgical invasiveness and by extension expected recovery time (although this has only been explicitly evaluated using recovery of pre-surgical body weight in one study [Yoshida et al. 2016]). Given the similarity in size, shape, and materials, all designs are likely comparable in terms of weight, but this information is generally not reported. There is also minimal variation across designs in how cumbersome the head-plates appear. However, it is possible that having a long bar protruding forward into the animal’s field of view is disruptive (Fig. 2c, left)—but this is pure speculation. Altogether, we do not see clear evidence for one design over another (for most applications). However, more complete reporting of head-plate features is needed.

#### Body restraint

With only two exceptions (Desjardins et al. 2019; Sakurai et al. 2020), studies opt for modest to extensive body restraint. Designs include a combination of gauze and tape, and a tube or box to cover the body (Table S3). It is reasonable to posit, that unrestrained mice will move more, but be less stressed. There is not enough evidence in the literature currently to properly weigh this trade-off. Of the two studies that do not use body restraint, neither quantify animal motion or stress (Desjardins et al. 2019; Sakurai et al. 2020). However, Desjardins et al. do collect videography of mice during fMRI which reveals behaviors such as grooming, which is indicative of calm, well acclimated animals. Data were excluded when movement was observed. This resulted in the removal of a fraction of trials akin to experiments done outside of the scanner (Desjardins et al. 2019).

### Acclimation

Without exception, the collection of awake mouse fMRI data is preceded by acclimating animals to the experiment. This can include exposure to the experimenter (through handling), immobilization (“*Immobilization*”), and scanner noise. Protocols vary substantially across labs. Elements include time to recover from surgery (if a head-post is utilized), how sources of stress (*stressors*, e.g. scanner noise) are introduced (either incrementally, through increasing the duration and intensity, or all at once), protocol length, and if a “mock-scanner” or the actual system is used. Figure S3 gives an overview of the 30 acclimation protocols published to date grouped by common authorship. Each lab tends to develop their own protocol and groups with more than one published study tend to apply the same protocol repeatedly with marginal modification over time. We note substantial variation across groups with some common themes (summary, Table 2). As differences between protocols can stem from planned scan length and use of anesthesia, this information is included for reference (“*BOLD-fMRI data acquisition*”).

**Table 2:** Common elements in acclimation protocols for awake mouse fMRI.

Study		Recover	Anes.	Handle	Mock	Real	Increment	Total	Scan length
Desai 2011		Unk	No	✕	✕	✓	✕	4	~8 mins × 4 runs
^a^ ^*^ Bergmann 2016		3	Yes	✓	✕	✓	✓	11	
^a^ ^*^ Shofty 2019		3	Yes	✓	✕	✓	✓	11	
^a^ ^*^ Asleh 2020		7	Yes	✓	✕	✓	✓	11	
^a^ ^*^ Bergmann 2020		3	Yes	✓	✕	✓	✓	11	
Ferris 2014		NA	Yes	✕	✓	✕	✕	4	5 or 7 mins
Moore 2016		NA	Yes	✕	✓	✕	✕	4	20 mins
Madularu 2017		NA	Yes	✕	✓	✕	✕	5	10 mins × 2 runs
Farra 2020		NA	Yes	✕	✓	✕	✕	4	15 mins
Sadaka 2021		NA	Yes	✕	✓	✕	✕	4	
Tsurugizawa 2013		7	Yes	✕	✓	✓	✓	4	40 mins
Yoshida 2016		11	No	✕	✓	✕	✕	7	5 mins
^a^ Matsubayashi 2018		11	No	✕	✓	✕	✕	7	10 mins
Takata 2018		11	No	✕	✓	✕	✕	7	8.5 mins
Tsurugizawa 2020		7	Yes	✓	✓	✓	✓	4	11 & 11.5 mins (2 runs total)
Takata 2021		11	No	✕	✓	✕	✕	7	10 mins × 2 runs
Abe 2021		7	Yes	✕	✓	✕	✕	10	7 mins
Tsurugizawa 2021 ^b^		7	Yes	✕	✓	✓	✓	6	10 mins
Han 2019		7	No	✕	Unk	Unk	✕	3	6 mins
Chen 2020		Unk	No	✕	Unk	Unk	✓	7	~6 or 8 mins × 5 runs
Xu 2021		7	Unk	✕	Unk	Unk	✓	7	~6 mins
Jonckers 2014		NA	Yes	✕	Unk	Unk	✓	8	5 mins
^b^ Harris 2015		NA	Yes	✓	✓	✕	✓	10	~17 mins
^*^ Desjardins 2019		7	Yes	✕	✓	✕	✓	Unk	Unk
Sakurai 2020		3	Unk	✕	✕	✓	✕	3	3.5 mins
Cover 2021		5	No	✓	✓	✓	✓	21	10 mins × 2–3 runs for 2 d
Dinh 2021		7	Yes	✕	✓	✓ & ✕	✓	10	2 mins × 2 runs
^b^ Gutierrez-Barragan 2022		10–15	No	✓	✓	✕	✓	20	32 mins
^b^ ^*^ Zeng 2022		21	No	✓	✕	✓	✓	~28	3.5 mins
Fadel 2022		NA	Yes	✕	✓	✕	✓	4	12 mins
	Range (days) Mean ± SD (days)	3–21 8 ± 4	Yes – 18 No—10	✕—21 ✓—9	✕—7 ✓—19	✕—15 ✓—12	✕ – 13 ✓—17	3–28 8 ± 6	3.5–40 19 ± 13

Days where data are collected are not included in “Total”.

Anesthesia (Anes.), Mock-scanner (“Mock”), real scanner (“Real”), stressors added incrementally (“Increment”), standard deviation (SD), Unknown (Unk), Isoflurane (Iso.).

^a^Additional training and imaging timepoints follow acclimation and the first imaging session these are not in the “Total”.

^b^Protocol includes gap-days which are not included in “Total”.

^c^Reward given (e.g. chocolate sprinkle, condensed milk, or sugar water) for acclimation training only (not for other purposes).

Studies that use head-posts, allow about a week for recovery (8 ± 4 d, mean ± SD), but the range is wide (3–21 d). Body weight drops following head-post surgery and takes 7–10 d to recover to baseline (Yoshida et al. 2016). Similarly, approximately a week is allotted to acclimate animals to the experiment (8 ± 6 d, mean ± SD), but again the range is wide (3–28 d). Exposure to stressors, in most studies (21/30), starts on day 1, with no prior handling-dedicated days, and most studies (16/30) use an incremental introduction of stressors (increasing duration and intensity across sessions). The majority (19/30) use a mock-scanner for acclimation and only move to the real system for data collection (see Table S4 for a summary of mock-scanner designs). This is unsurprising given the high financial cost of scan time. Common elements of mock-systems include restraint devices (same or equivalent to those used in the real system), a dark chamber (to mimic the bore), and speakers capable of matching scanner volumes (~87–120 dB) (Madularu et al. 2017*^a^*; Abe et al. 2021; Cover et al. 2021; Dinh et al. 2021; Gutierrez-Barragan et al. 2022). The level of detail with which mock-systems are described varies across studies. This makes it challenging to compare how well different groups recreate the in-scanner experience and what effect this has on training success. One study, by Dinh et al., explicitly tests whether real-system training results in better outcomes and concludes that motion is reduced, and the quality of the fMRI data is improved when training includes time in the real system (Dinh et al. 2021).

Despite variability in acclimation protocols across studies, we try to draw some general conclusions. If we consider total duration, all but three studies acclimate animals for < 2 wk, and approximately half of those acclimate animals for < 1 wk (Figure S4a). Across studies, there is no clear relationship between quantitative measures of stress (respiration rate, heart rate, and corticosterone) and days of acclimation training (“*Animal stress*”). This is largely due to variability across studies in quantitative measures of stress (“*Animal stress*”). Number of acclimation training days and motion (translation or framewise displacement, FD) are plotted in Figure S4b (“*Data processing approaches*”). Together, the available stress and motion data indicate that with 10–14 d of acclimation, there are low levels of, or a drop in, corticosterone (Harris et al. 2015; Dinh et al. 2021; Gutierrez-Barragan et al. 2022), any decrease in heart rate (Tsurugizawa et al. 2013, 2020; Yoshida et al. 2016) or breath rate (Harris et al. 2015; Madularu et al. 2017*^a^*) has occurred, and motion is reproducibly < 50 μm. Thus, a *conservative* guideline is to adopt a 10–14-d protocol. There is little evidence that longer protocols garner significant benefits, but there are only three studies that carry out these experimental designs (Cover et al. 2021; Gutierrez-Barragan et al. 2022; Zeng et al. 2022). Shorter protocols, 6–8 d, may prove to be just as effective as 10–14-d protocols, especially if key training elements (e.g. time in the real scanner instead of a mock-system (Dinh et al. 2021)) are included in the design. Finally, there do seem to be gains (lower stress and motion) if training goes beyond 4–6 d.

A handful of studies (8/30) describe conducting acclimation/imaging at the same time of day to minimize the effects of circadian rhythms (Table S5). Whether or not the time of day is kept consistent, most studies acquire data during the day (light-phase), only two studies (both from one group) report using a reverse light cycle (Bergmann et al. 2016, 2020). Overall, the interaction between learning, stress, and light/dark phase has yet to be investigated systematically in the context of awake mouse fMRI. Similarly, another promising research direction is the study of spontaneous sleep which has only recently been investigated (Yu et al. 2023) in line with a body of complementary work using other imaging modes (e.g. optical techniques, see Miyamoto 2022).

### Animal stress

The goals of acclimating mice to awake fMRI are to (i) reduce subject motion and (ii) reduce stress. These are inter-related since (in theory) an animal that is well acclimated (less stressed) will struggle less (less motion). Though, it should be noted that freezing is an established fear response in mice (Cybulska-Kłosowicz 2016), and an awake animal that is well acclimated will engage in a normal range of movements (e.g. grooming [Desjardins et al. 2019]) if these are not restricted by the set-up. Beyond reducing motion, reducing stress can be important due to the wide range of effects that both stress and noradrenaline have on physiology, brain activity (McEwen 2007), and by extension functional connectivity (Wang et al. 2022). These considerations are not limited to the acute stress experienced during data acquisition, but also include chronic effects associated with acclimation training (Low et al. 2016; Han et al. 2019). The wide range of measures that have been employed to estimate stress in mice being acclimated to awake fMRI are summarized in Table S6.

**Fig. 3:**
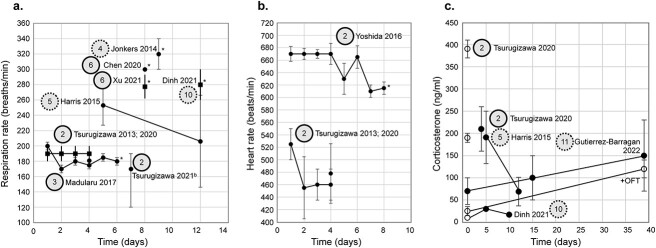
Quantitative measures of stress. Respiration rate (a), heart rate (b), and corticosterone (c) are the three most common quantitative measures of stress used to evaluate acclimation (Table S6). All data (a, b, & c) are collected from adult male C57BL/6 J mice except a subset of heart rate measurements obtained in the 2020 Tsurugizawa et al. study (Tsurugizawa et al. 2020) which included 15q dup animals (a model of 15q11–13 duplication (15q dup), a copy number variation model of autism on a C57BL/6 J background) (Table 1). Notably, 15q dup mice show no differences in heart rate compared to wild-type (WT). Moreover, corticosterone measures in (Tsurugizawa et al. 2020) were only acquired from WT animals. Thus, sex and strain are not driving inter-study differences. On all plots, study (1st author and date) is indicated. An asterisk indicates physiological data collected during fMRI acquisition, or on the “imaging day” if in-scanner training was performed (Fig. S3). In all plots, some values are taken from the text while others are inferred from figures. Data from the same study are linked by lines. Reported error (standard deviation, or standard error, see Table 1), is indicated by error bars (with caps). (a) Studies which reported a range, (Tsurugizawa et al. 2020; Dinh et al. 2021), are plotted as squares with solid lines (no caps) indicating the range and the data point indicating the middle of the range. (c) Studies reporting measures of corticosterone. Dinh et al. report longitudinal data (*n* = 2) acquired before training (day 0) as well as after 5 and 10 d of acclimation. Data are acquired from the same mice (linked with a solid line). Control data (day 0) are plotted using an open circle. Tsurugizawa et al. report data from three groups: *N* = 5 (without training, day 0, open circle), *n* = 4 (day 1, solid circle), and *n* = 5 (day 4, solid circle). It is unclear whether data are acquired from the same animals (no lines). Finally, Gutierrez-Barragan et al. collect data from co-housed control (*n* = 7) and acclimated (*n* = 7) groups. Corticosterone measures are acquired after handling (prior to mock-scanner training, Fig. S3) in the acclimated group, as a baseline (day 0, solid circle) alongside measures from a control group (no handling, day 0, open circle). Handling is not considered part of training in this plot. Following 24 d of mock-scanner training (solid circle, solid line) or regular housing (open circle, dotted line) corticosterone levels are re-measured. Notably, control mice undergo an open field test (OFT) prior to this second corticosterone measurement. A version of this figure with color-coding is included in the supplemental material (Fig. S5).

Although most studies report some measure of stress, there is an appreciable fraction (9/30) that do not (Moore et al. 2016; Takata et al. 2018; Shofty et al. 2019; Asleh et al. 2020; Bergmann et al. 2020; Farra et al. 2020; Sakurai et al. 2020; Abe et al. 2021; Takata et al. 2021). Of the studies that report quantitative measures, the most common are: (i) respiration rate (11/30), (ii) heart rate (4/30), (iii) and corticosterone (4/30) (Fig. 3). A subset reports changes in physiology over time, or, for corticosterone, measures from acclimated mice are compared to a control (imaging naïve) group (Tsurugizawa et al. 2020;Dinh et al. 2021; Gutierrez-Barragan et al. 2022).

Overall, a wide range of values for quantitative measures of stress (Fig. 3) are reported across studies. Discrepancies are not attributable to sex or strain (Table 1). Unfortunately, the measurement that is most common and easiest to access, respiration rate, is likely the least informative. The small number of studies (4/30) that report corticosterone collectively show the most cross-study agreement and evidence of acclimation-related stress reduction (Harris et al. 2015; Tsurugizawa et al. 2020; Dinh et al. 2021; Gutierrez-Barragan et al. 2022). Further, recent advances make it possible to obtain measures of corticosterone from small blood volumes that can be collected repeatedly from mice using a tail nick (as done by Gutierrez-Barragan et al. following the methodology described in [Kim et al. 2018]) replacing previous approaches which required large blood volumes and necessitated euthanasia. However, there is a large and non-overlapping range in reported “non-stressed” or “target” corticosterone concentrations that have been cited (mostly from work in mice where stress is induced more broadly). Specifically, Xie et al. report corticosterone levels in “unstressed” as 60–100 ng/ml and “stressed” as >150 ng/ml (Xie et al. 2013), whereas Cawthorn et al. report “unstressed” as <20 ng/ml and “stressed” as 20–80 ng/ml (Cawthorn et al. 2016), and finally, Zhang et al. describe “unstressed” as ~12 ng/ml and “stressed” as ~25 ng/ml (Zhang et al. 2015). To date, only two studies on awake mouse fMRI explicitly place measures of corticosterone elicited by acclimation training in a broader context (Dinh et al. 2021; Gutierrez-Barragan et al. 2022). The most complete description being by Gutierrez-Barragan et al. (see their Supplementary Fig. 1e, Gutierrez-Barragan et al. 2022).

Overall, the data indicate that stress is challenging to evaluate, and we find no clear target, which future work should use as a benchmark, in the existing literature. However, efforts to reduce, and carefully consider the effects of stress are important. We propose that the best strategy currently is to include appropriate control groups which allow the stress-related effects of acclimation to awake fMRI to be quantified. Notably, this is not a problem that is unique to studies on mice. Most awake mouse fMRI studies rely on complementary work in awake rat fMRI (particularly work by King et al. 2005) for choosing physiological measures of stress and for designing acclimation protocols. Yet, there are similar problems in this body of work with how stress has been quantified. An in-depth discussion of the awake rat fMRI literature (including quantitative measures of stress) is covered in “*Awake fMRI in rats*” section. Further, there are differences in how mouse and rat physiological measures relate to stress that are not yet fully understood, and preliminary evidence that there are differences in how these species respond to acclimation training (Jonckers et al. 2014; Madularu et al. 2017a).

### BOLD-fMRI data acquisition

A wide variety of approaches are used to collect, process, and analyze awake mouse fMRI data. Many decisions are dictated by available hardware and the scientific question being investigated. Overall, it is not yet clear whether best practices for the collection and handling of awake mouse fMRI data should differ from those used for anesthetized experiments (which have been summarized elsewhere [Grandjean et al. 2020, 2023; Mandino et al. 2020]). While directly addressing this question is beyond the scope of this article, it would be a valuable contribution to the literature. More widespread data sharing would facilitate this work (Table 1), but given the uniqueness of awake mouse fMRI data, it is unsurprising that most datasets are not (yet) publicly available.

Awake mouse fMRI studies are conducted on high field systems (range, 4.7–14 T) with the majority (26/30) performed at 7 or 9.4 T (Fig. S6a). An appreciable fraction (6/30) use custom coils while most use commercially available coils, at either ambient temperature (15/30), or cooled—CryoProbe (Bruker BioSpin), (9/30)—which improves SNR and BOLD sensitivity (Ratering et al. 2008; Baltes et al. 2011; Niendorf et al. 2015). There is an approximately equal split between GE-, SE- EPI implementations, and other sequences (Table S7). The TR (repetition time)—a key parameter in fMRI—(Figure S6b), has a particularly wide range 0.35–15.00 s (median 2.00 s). This is due to both hardware limitations and the scientific purposes of different studies. The amount of data acquired also varies substantially across studies (range, 3.5–40 min) (Figure S6c). The spatial (in-plane, Figure S6d, and through-plane, Figure S6e) resolution depends on the field-of-view (FOV), or brain coverage, other sequence parameters (e.g. TR) and hardware capabilities (B_0_ and coil). Across studies, the average ± SD in- and through-plane resolution is 0.22 ± 0.07 mm, and 0.6 ± 0.2 mm, respectively.

### Data processing approaches

Once data are obtained, various processing steps and criteria for data/subject exclusion are applied. These steps are taken to remove noise from the data and to facilitate analyses that help answer the study question. Processing steps, the software used, and exclusion criteria based on motion are summarized in Table S8. Various software packages are used for data denoising and analyses. The most popular are SPM (19/30) (Wellcome Trust Center for Neuroimaging, UK), MATLAB® (Mathworks, Natick, MA, USA; 14/19), FSL (7/30) (Smith et al. 2004; Woolrich et al. 2009; Jenkinson et al. 2012), AFNI (6/30) (Cox 1996) and ANTs (5/30) (Avants et al. 2011). All studies apply motion correction, while approximately half (16/30) regress motion parameters from the timeseries (Power et al. 2014). Most use 6- or 12-parameter models (9/30 and 4/30, respectively), while a few (3/30) investigate more parameters. Of the articles in this review that compare different motion regression approaches, none find a significant impact on study outcomes (Chen et al. 2020; Dinh et al. 2021; Gutierrez-Barragan et al. 2022). Although Dinh et al. note that using 12 in place of six parameters improves time-course quality (with some regional dependence) (Dinh et al. 2021).

**Fig. 4:**
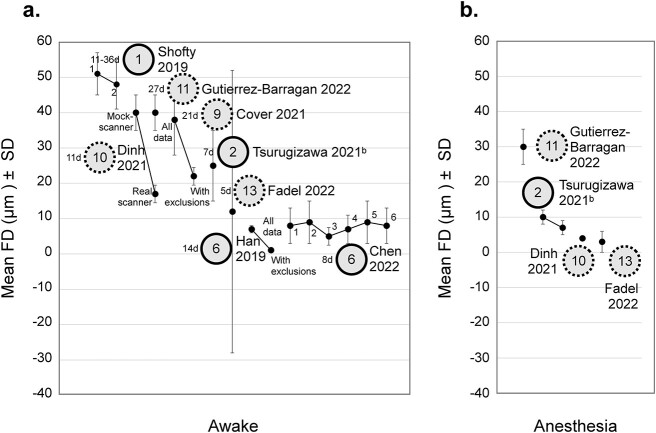
Motion outcome measures. Studies that report motion using either a six-parameter estimate (Fig. S7) or FD (a & b). In (a), data from awake mice are plotted. In (b), data from anesthetized mice are plotted. Data from the same study (different groups) are included. A version of this figure with color-coding is included in the supplemental material (Fig. S8).

Nuisance regressors other than motion include: detrending (12/30), demeaning (4/30), and regressing cerebral spinal fluid (9/30), white matter (6/30), and the global signal (4/30). Approximately half (16/30) apply no frequency filtering. Of studies that do, the most common band is 0.01 < *f* < 0.01 Hz (6/30). Most studies (21/30) apply spatial smoothing; the median (± SD) size being: 600 ± 180 μm. Two-thirds of studies (20/30) do not remove imaging frames from the timeseries that correspond to epochs of high subject motion (i.e. motion censoring). Of those that do, most (6/30) use FD > 0.05 mm (range, 0.075–0.15 mm). Finally, there is a roughly even split (13 vs. 17 of 30) between studies that do versus do not apply criteria for trial, run, scan, or session exclusion (usually based on motion or fraction of frames excluded for high motion). The scientific goals of different studies can dictate the processing steps, as can the characteristics of the data (e.g. smoothing is usually based on voxel size). Table S8 summarizes exclusion criteria based on motion. Note that motion estimates are used both as a criterion for data exclusion and as an outcome measure. The amount of data which passes these thresholds (the criterion), and the average motion reported (the output measure) are summarized in Table S9 for studies that report six-parameter motion estimates and Table S10 for studies that report FD. Average motion (output measure) is plotted in Fig. 4.

Most studies (22/30) report the amount of data excluded based on motion criteria and many report some measures of motion. Yet, there is still a large fraction that, beyond applying motion correction, do not report any metrics (8/30) (Xu et al. 2021; Abe et al. 2021; Farra et al. 2020; Takata et al. 2018; Matsubayashi et al. 2018; Moore et al. 2016; Jonckers et al. 2014; Tsurugizawa et al. 2013). This is concerning given the importance of this measurement in awake mouse fMRI experiments. To some extent, the coverage as well as spatial and temporal resolution of the data (Table S7) affect motion estimates (Chen et al. 2020). However, on average, studies achieve head motion < 50 μm (translation and rotation, or FD). Encouragingly, the small fraction of studies that compare motion estimates before and after acclimation show improvement (3/30) (Desai et al. 2011; Tsurugizawa et al. 2020; Zeng et al. 2022), and studies that compare awake to anesthetized animals show comparable outcomes (5/30) (Yoshida et al. 2016; Dinh et al. 2021; Tsurugizawa and Yoshimaru 2021; Fadel et al. 2022; Gutierrez-Barragan et al. 2022). This is despite the wide array of immobilization devices and acclimation protocols that are used (“*Immobilization*” & “*Acclimation*”). These generally high rates of success translate into most studies excluding very little or no data based on motion (Figure S9).

Beyond mouse and scan, some studies report exclusion of trials (5–20%) (Desjardins et al. 2019) or imaging frames (1–11%) (Takata et al. 2021; Gutierrez-Barragan et al. 2022). A few additional exclusion criteria are summarized in Table S11. A few studies quantify SNR (Jonckers et al. 2014; Chen et al. 2020) and temporal SNR (tSNR) (Desai et al. 2011; Yoshida et al. 2016; Han et al. 2019; Chen et al. 2020) as a measure of acclimation success; but the outcomes are mixed. Some show “high” SNR/tSNR in awake animals, while others do not. Further, these measures are not easily compared across studies due to various ways of reporting and substantially different outcomes (“high” SNR/tSNR differing by an order of magnitude across studies).

Although some characteristics of fMRI data obtained from experiments on awake versus anesthetized animals differ (e.g. frequency and magnitude of motion), many of the general principles for how fMRI data should be processed to remove noise (and preserve signal) still apply. Thus, available general guidelines (e.g. Power et al. 2014) for how to treat fMRI data should be followed.

## Awake fMRI in rats

**Fig. 5:**
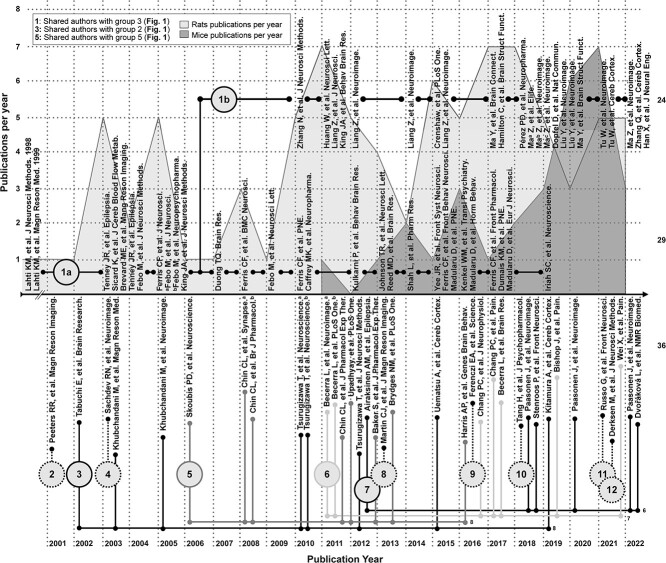
Taxonomy of articles identified in our systematic review of the awake rat fMRI literature. Summary of articles which collect fMRI data from awake rats (as Fig. 1 does for mice). Articles are listed in chronological order. The number of articles on rats published per year is indicated by a gray histogram background. For reference, the number of articles on mice published per year is also indicated. Articles which share co-authors, and by extension an experimental approach, are linked by lines. Groups which have contributed one article to the literature are indicated by numbers with dashed outlines. The seminal article from each independent group is numbered. The total number of articles in subgroups 1b, 1a, and all others are noted on the far right (24, 29, and 36, respectively). Groups (labeled here as: 1, 3, and 5) have also published awake mouse data (see legend). A version of this figure with color-coding is included in the supplemental material (Fig. S10).

Given the strong influence that work in awake rats has had on awake mouse fMRI, we include an overview of this literature. Using a systematic search (PubMed, Table S1), we identify 88 relevant articles (years 1998–2022, Table S12 and Fig. 5). Of these, over half (53/88 studies) come from two subgroups (1a Table S13, and 1bTable S14) with shared authorship (depicted above the timeline in Fig. 5). As above (for mice), groups (and subgroups) are based on authorship and—by extension—methods. The remaining articles (36/88, depicted below the timeline, Fig. 5) are split between four groups with multiple publications, and seven groups with a single publication (Table S15). In total, there are 12 independent groups—one less than in the awake mouse fMRI literature (Fig. 1). Further, despite there being no common authorship, a sizable fraction (11/36) “below the timeline” use the restraint system and acclimation protocol outlined by Group 1 (bringing the total up to 64/88 or 72%). Herein, we call this the “dominant approach.”

### Acclimation training in awake rat fMRI

Without exception, those using the dominant approach anesthetize rats (usually with isoflurane) prior to placing them in a commercialized restraint system (Animal Imaging Research, Holden MA, USA). No surgically implanted devices are used for head immobilization and the mounting procedure has not changed substantially since the original descriptions (Lahti et al. 1998; Tenney et al. 2004). The only notable changes are that the use of ear bars stopped (Madularu et al. 2015; Ma et al. 2018c) and limb restraint started (Zhang et al. 2010; Ferris et al. 2015; Han et al. 2022). The device restrains the head using a plastic holder with integrated radio frequency coil, incisor bar, and custom plastic part which depresses the snout. The body is restrained using a suspension system that uncouples the head and body (to reduce motion artifacts).

Group 1a (29/88 studies), in early years (years 1998–2007), did no acclimation training (Table S13, top rows). Later work (years 2004–2019) includes acclimation training in a mock-scanner (Table S13, bottom rows). No handling of rats by the experimenter precedes training, and there is no incremental introduction of stressors (with one exception [Reed et al. 2013]). The mean (± SD) and range of acclimation training time is 4 ± 2 and 0–8 d. Most imaged rats are male (564/729) and belong to either the Sprague Dawley (SD) or Long Evans (LE) strain (with one exception [Tenney et al. 2004]). Of studies that report excluding rats based on motion (10/29), the discarded fraction of the sample is high, 23 ± 16% and 5–60% (mean ± SD and range), and increases over time (years 1998–2017, R^2^ = 0.51 *P* = 0.02, MATLAB, *corr*). We suspect that this tracks with emerging concerns in the fMRI community regarding motion in these data. On the other hand, a similar fraction (13/29 studies) excludes zero data. None of the studies in Group 1a apply motion correction or other common data-denoising strategies. Exclusions are usually based on a visual inspection of the data.

In Group 1b (24/88 studies), rats are acclimated in a mock-scanner for longer, relative to Group 1a (7 ± 1 and 3–8 d, Table S14). All but two studies (Huang et al. 2011; King et al. 2011) use an incremental approach where immobilization time is increased in 15-min steps until the total scan time is reached (total time is repeated on remaining training days). No handling of rats precedes acclimation training. All rats imaged by studies in Group 1b are male (512/529) or unspecified (17/529) (Han et al. 2022) and most (21/24) belong to the LE strain. Although almost half (13/24) do not report exclusions (Table S14), less data is discarded (due to motion) by studies in Group 1b relative to 1a (5 ± 8% and 0–8%). In general, more widely accepted data processing steps are applied, including frame censoring, motion correction (SPM), regression of motion estimates (six or more), and requiring that a minimum amount of data be retained following censoring (Power et al. 2014). The most recent and complete description of data processing methods is given by Liu et al. (2020). To further address motion, a few studies regenerate findings in a low-motion subgroup (<125 μm FD) (Liang et al. 2012; Hamilton et al. 2017; Ma et al. 2017), or in both a low and high motion subgroup (Ma and Zhang 2018; Liu et al. 2019).

Groups other than Group 1 that use the dominant approach (11/88 studies) apply methods that are more like subgroup 1a than 1b. They apply a short acclimation protocol (3 ± 1 and 2–5 d) and exclude similarly high amounts of data (19 ± 10% and 7–38%). On the other hand, most (7/11) incrementally increase immobilization time (like subgroup 1b), and a few (3/11) add daily handling to familiarize animals to the experimenter prior to acclimation training (Table S15). These studies are herein referred to as Group 1-like.

The remaining studies (36/88) do not follow the dominant approach. Of these, a minority use a preparation that does not require anesthesia to mount (or remove) rats into (or out of) the set-up (8/36 of 88). Unsurprisingly, these studies use longer acclimation protocols (14 ± 4 and 9–21 d). A small number (16/36 of 88) perform a surgery to affix a device to the skull to aid with head immobilization (e.g. screws or nuts, pockets to accommodate bars, or 1–3 posts). Overall, Groups 2–12 (below the timeline in Fig. 5), implement acclimation protocols that are less than a weeklong (6 ± 4 and 0–21 d) but there are exceptions (10/36 of 88). Most rats that are imaged by Groups 2–12 are male (588/624). Thus, in total (across all 88 studies), 9% of imaged rats are female (173/1882). Finally, although LE and SD are popular, there are more strains (e.g. Wistar) that are imaged by Groups 2–12 (Table S15). No publications (from any group) make cross-sex or strain comparisons.

### Exclusion rates in awake rat fMRI

**Fig. 6:**
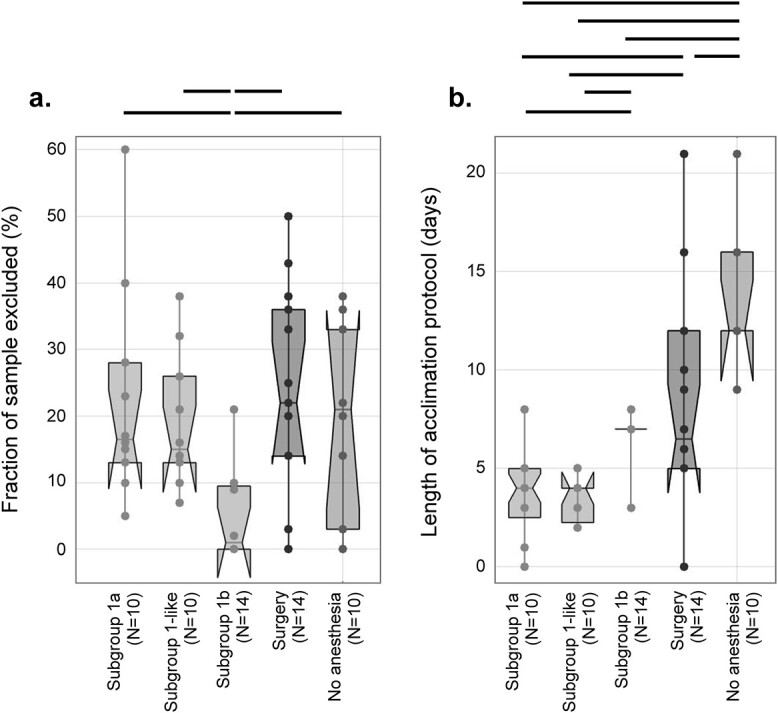
Exclusion (based on motion) and length of acclimation protocol for approaches in rats. Studies in rats are divided into five columns: Subgroup 1a, group 1-like, subgroup 1b, “surgery,” and “no anesthesia.” group 1-like are studies that do not share co-authors with group 1 (Fig. 5) but apply the same immobilization and acclimation methods (Table S15). “Surgery” are studies that use a surgically implanted device to aid with head immobilization. “No anesthesia” are studies that acclimate animals and perform data collection without the use of an anesthetic for mounting or unmounting of the rat into or out of the set-up. In (a), the fraction of data excluded due to motion is plotted. In (b), the length of the acclimation protocols used is plotted. Significant differences (*P* < 0.05, MATLAB, *ttest2*) are indicated by black bars.

The amount of data discarded due to motion and the time spent acclimating rats to awake fMRI are plotted in Fig. 6 for subgroups 1a, 1b, Group 1-like, as well as studies that use no anesthesia (pooled across Groups 2–12), or a surgical implant for head immobilization (Groups 2–12). From Fig. 6 we infer that subgroup 1b excludes the smallest amount of data and implements an acclimation protocol that is longer than subgroup 1a (and Group 1-like), but shorter than those that do not use anesthesia. Although this comparison helps us draw some broad inferences, it should be noted that studies use different exclusion criteria (e.g. motion threshold) and that there is considerable heterogeneity within and across the categories we impose.

### Quantitative measures of stress in awake rat fMRI

Of all studies (64/88) that apply the dominant approach, stress is quantitatively measured in only five (King et al. 2005; Upadhyay et al. 2011; Becerra et al. 2011a; Liang et al. 2014; Brydges et al. 2013). In lieu of measuring stress, this body of work overwhelmingly relies on one study by King et al. (2005) (also frequently referenced in the awake mouse literature). In this key study, rats (*n* = 8 male, SD) are acclimated using both a mock-scanner (days 2, 3, 4, 6, and 8) and the real system (days 1 and 5). Respiration, heart rate, and corticosterone are measured on days 1 (real-scanner), 3 (mock-scanner), 5 (real-scanner), and 8 (mock-scanner) as well as at baseline (75 ± 25 ng/ml). Decreases between days 1 and 5 (both real-scanner days) in respiratory rate (160 ± 20 to 105 ± 7 bpm), heart rate (410 ± 20 to 335 ± 20 bpm), and corticosterone (240 ± 70 to 125 ± 25 ng/ml) are found. Critically, King et al. conduct day 1 training inside the real-scanner and compare subsequent measurements to this (elevated) baseline. This choice surely impacts how these measures should be interpreted. Further, this step (day 1 inside the real-scanner) is not replicated in subsequent work.

**Fig. 7:**
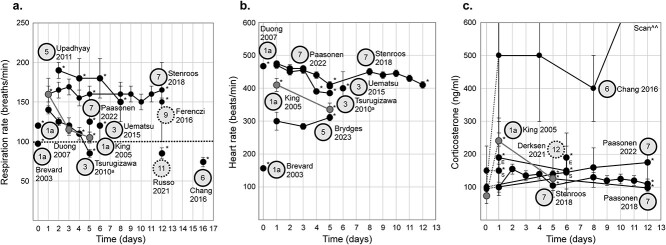
Quantitative measure of stress from rats. Respiration rate (a), heart rate (b), and corticosterone (c) for awake rat fMRI studies. Data from the same study are linked by lines. Dotted lines link data collected at baseline (time, 0 d) and data collected during acclimation training or imaging. Data points indicated by an asterisk are collected on imaging days (real-scanner). In (a), a bold horizontal dotted line (at 100 bpm) is shown because many studies only proceed with fMRI data acquisition after this rate has been exceeded (as an indication that the effects of anesthesia have worn off) Fig. 5. Strains break down as: SD (Brevard et al. 2003; King et al. 2005; Duong 2007; Tsurugizawa et al. 2010a; Chang et al. 2016; Paasonen et al. 2022), Wistar (Uematsu et al. 2015; Paasonen et al. 2018; Stenroos et al. 2018; Derksen et al. 2021), Lister hooded (Brydges et al. 2013; Russo et al. 2021), LE (Upadhyay et al. 2011), and unknown (Ferenczi et al. 2016). Sex is not specified by two studies (Duong 2007; Ferenczi et al. 2016) but is otherwise male. Two studies do not use anesthesia (Chang et al. 2016; Paasonen et al. 2022). In (c), two data points appear on days 1 and 6 (Derksen et al. 2021). These are collected at the “start” (S) and “end” (E) of the imaging session (as indicated on the plot). A version of this figure with color-coding is included in the supplemental material (Fig. S11).

Across all studies, 15/88 provide quantitative measures of stress (respiration, heart rate, or corticosterone)—a much smaller fraction than the awake mouse literature (11/30). The measures of stress that are reported from awake rats are plotted in Fig. 7. Overall, despite differences being observed within studies, no clear target ranges emerge across studies. Rat strain (and sex) do not appear to be obscuring any patterns (but no study explicitly investigates these effects). Of note, Russo et al. (2021) collect corticosterone measures from feces at 24 and 72 h. After acclimation training or imaging as an acute and chronic stress measure (see their Fig. 1b). These data are not included in our Fig. 7, since all other corticosterone measures come from blood samples. However, we thought that the approach was intriguing (given that it is noninvasive) and that the data show some interesting results (albeit in a small sample, *n* = 6 males).

### Motion in awake rat fMRI

**Fig. 8:**
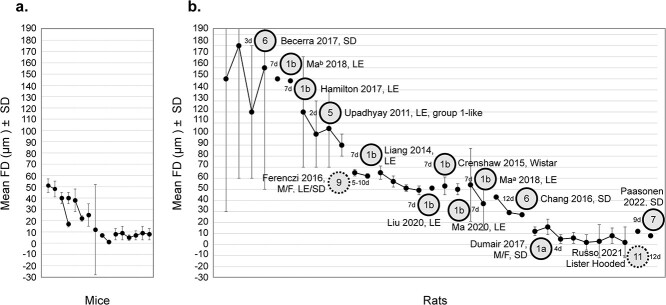
Motion outcome measures from rats. Studies in mice (a) and rats (b) that report mean framewise displacement (FD). Data in (a), from mice, are reproduced from Fig. 4 for reference. Data in (b), are from rats Fig. 5. Data from the same study are linked by lines. In (b), subgroups 1a and 1b, as well as group 1-like are noted alongside rat strain and the length of the acclimation protocol. Two studies, by Ferenczi et al. (2016) and Dumais et al. (2017) use both male and female rats—Indicated as M/F—(all others use only male rats). Three studies, by Chang et al. (2016), Ferenczi et al. (2016) and Paasonen et al. (2022), use a surgical implant to aid in reducing head motion. Chang et al. (2016) and Paasonen et al. (2022) are the only two that do not use anesthesia to mount and unmount rats from the immobilization set-ups. A version of this figure with color-coding is included in the supplemental material (Fig. S12).

A total of ~30% of studies (25/88) specify quantitative criteria for data exclusion based on motion. Most report FD (median cutoff: 0.25 mm). Those that report translation (6/88 studies)—all subgroup 1a—report X & Y: 0.109–0.312, and Z: 0.12–0.312 mm (based on voxel dimensions). A handful (17/88 studies) report motion as an outcome measure. Three studies—all from subgroup 1a—report translation measures for X: 4–60 μm, Y: 7–150 μm, and Z: 18–141 μm (King et al. 2005; Ferris et al. 2008; Iriah et al. 2019). Mean FD (reported by 14/88 studies) is plotted in Fig. 8b (alongside measures from mice, Fig. 8a, reproduced from Fig. 4b for reference). Half of the studies plotted in Fig. 8b come from subgroup 1b and use the same sex (male), restraint system, and 7-d acclimation protocol (Table S14). All but one study (Crenshaw et al. 2015) uses the LE stain. Yet, the range of resultant motion is wide 37–150 μm indicating significant inter-implementation outcomes. Except for one study, by Dumais et al. (2017) which appears to be an outlier, those that use a longer acclimation protocol, 9- or 12-d (Chang et al. 2016; Russo et al. 2021; Paasonen et al. 2022), observe low motion (8–27 μm), and those that use a shorter protocol, 2 or 3 d (Upadhyay et al. 2011; Becerra et al. 2017), observe high motion (90–180 μm). Of the three studies that use a surgically implanted device to aid with head motion reduction, those that also use a long (12-d) protocol, versus a short one (5–10 d), observe lower motion (Chang et al. 2016; Ferenczi et al. 2016; Paasonen et al. 2022) (Fig. 8b). Notably, Paasonen et al. (2022) use an imaging sequence that is less sensitive to body movements than other studies.

## Conclusions from awake rat fMRI

Most studies on awake rats follow an approach that has dominated the literature since 1998 (Lahti et al. 1998). We find that the evidence which supports the use of this approach (King et al. 2005) is weak and outdated based on the current literature. Further, implementations which have relied on King et al. have done a poor job of replicating their methods (i.e. performing acclimation training in the real-scanner). Overall, relative to studies in awake mice, methodological diversity in the rat literature is low (especially given the 13-yr head start). The widespread use of one protocol makes it possible to infer some general conclusions about inter-implementational variability (e.g. the wide range of motion outcome measures in Fig. 8 for Group 1b), but it also means that there are fewer strategies to compare.

In opposition to the mouse literature, surgical implants for head immobilization in rats incur marginal benefits. This is in-part due to their larger size (and strength) which does lead to more detachments (and subsequent exclusion). However, this may only be true when short acclimation protocols (a week or less) are used and stressors are not introduced slowly. Along this line, there is some evidence that longer acclimation protocols (7–12 d), with very gradual stressor introduction result in more favorable outcomes (low motion and exclusion rates—but not lower stress). Against many claims in the literature, we find no evidence that effective acclimation training reduces respiration, heart rate, or corticosterone across studies (Fig. 7). This means that there is no generalizable way to measure stress reduction associated with acclimation training (despite within-study observations). Further, as in the mouse literature, there is a profound need for more work using female animals.

## Awake versus anesthetized mice, when is it better to do one or the other?

Reasons to perform awake mouse fMRI (in place of anesthetized experiments) include: (i) the physiological effects of anesthesia influencing the biological question of interest, (ii) that anesthesia reduces the BOLD signal (reducing study effect size), (iii) that anesthetized animals cannot perform tasks, and (iv) that data from awake animals are a more appropriate match to human data, or data from other modalities (e.g. optical imaging) where the use of anesthesia is less common. Somewhat surprisingly, given this array of reasons, the amount of direct evidence for differences in study outcomes between awake and anesthetized data is somewhat limited. Studies that directly compare awake to anesthetized data (8/30) are summarized in Table 3. There is also a collection of studies (5/30) where there is no anesthetized counterpart. These include studies where certain tasks, olfactory cued (Han et al. 2019), fear conditioning (Harris et al. 2015), and reward licking (Sakurai et al. 2020; Cover et al. 2021) are performed, or data from multiple modalities are compared (Desjardins et al. 2019). This leaves the majority (17/30) that either provide a very limited comparison to anesthetized literature (Tsurugizawa et al. 2013; Bergmann et al. 2016; Moore et al. 2016; Asleh et al. 2020; Chen et al. 2020; Sadaka et al. 2021; Zeng et al. 2022), or no comparison at all (Ferris et al. 2014; Madularu et al. 2017a; Matsubayashi et al. 2018; Takata et al. 2018; Shofty et al. 2019; Bergmann et al. 2020; Farra et al. 2020; Tsurugizawa et al. 2020; Abe et al. 2021; Takata et al. 2021). As expected, studies that include a direct comparison between awake and anesthetized groups find broad differences in outcome measures despite there being a wide variety in anesthesia (and awake) imaging protocols, scientific questions, and study designs.

**Table 3:** Differences between awake and anesthetized mouse fMRI data.

Study	Anesthesia protocol(s)	Differences between awake and anesthetized outcome measures
Desai 2011	Iso. (0.7%)	Increase in response magnitude in target and off-target areas and an increase in inter-regional temporal correlations.
Yoshida 2016	Iso. induction (4%), maintenance (1.5%), stop at 10 min then med. (0.3 mg/kg) s.c. bolus, then infusion (0.6 mg/kg/h)	Temporal SNR greater and less variable. Both show bilateral functional connectivity (ICA). Basal ganglia network unique to anesthetized animals. MANCOVA reveals an increase in connectivity in the retrosplenial cortex and hippocampus. FC generally more abundant (in a ROI-by-ROI comparison).
Tsurugizawa 2021 ^b^	Group1: Iso. (0.8%), Group2: Iso. (0.5%) & med. (0.1 mg/kg) s.c. bolus, then infusion (0.05 mg/kg/h). *Iso. dose “lower” due to no scavenging and tight space within the CryoProbe coil*	Study purpose is to compare functional connectivity (static and dynamic). Data are from the same animals during one session (Fig. S1). Dynamic functional connectivity shifts from positive to negative (LCN, Hypo, and AUD-VIS) to dominant connectivity in the LCN with anesthesia. Static functional connectivity shows a reduction in intra-hemispheric connectivity as well as in subcortical areas (agranular insular cortex, thalamic nuclei, and part of the limbic system) with anesthesia. Inter-mouse similarity in static functional connectivity is greater in awake mice. Consistent with (Jonckers et al. 2014).
Xu 2021	Iso. induction (3.5%), maintenance (1.5%), stop at 5 min then dexmed. (0.025 mg/kg) i.p. bolus, then infusion (0.05 mg/kg/h).	Globally greater CMRO2 (with some regional differences). The cortex shows a smaller change than subcortical regions (large change in auditory cortex). Results in-line with PET (mouse) and fMRI data (human).
Jonckers 2014	Group1: α-chloralose (120 mg/kg), Group2: Urethane (2.5 g/kg), Group3: Iso. (1%)	Reduction in interhemispheric functional connectivity in α-chloralose and urethane groups relative to iso. and awake groups. Iso. showed the lowest localized functional connectivity and high inter-subject variance.
Dinh 2021	Ketamine/xylazine (100 mg/kg and 10 mg/kg) i.p., supplemented at 30–45 min intervals (25 mg/kg and 1.25 mg/kg)	BOLD responses to visual stimulation are faster (all regions) and stronger in subcortical areas. There are also regional (visual area 1) differences in BOLD responses depending on stimulation frequency. At 10 Hz, a sustained positive response (anesthetized) versus a biphasic or negative response (awake). Some regions only respond to stimulation in the anesthetized group (extrastriatal postrhinal area and non-visual subiculum complex). Consistent with (Desai et al. 2011) and (Desjardins et al. 2019).
Gutierrez-Barragan 2022	Group1: Halothane (0.75%), Group2: Iso. (0.5%) & med. (0.05 mg/kg) i.v. bolus, then infusion (0.1 mg/kg/h)	Anatomically comparable networks, but topologically configure to maximize interregional communication. Data exhibit unique spatiotemporal dynamics with characteristic coactivation patterns where arousal-related forebrain nuclei participate prominently alongside anti-coordination between visual–auditory and polymodal cortical areas (DMN). Patterns are configured as network attractors alongside greater coupling between cortical and subcortical areas. Consistent with non-human primates and humans.
Fadel 2022	Iso. (2.5%)	(i) Differences in ^31^P spectra: PCr and Pi are elevated indicating less energy reserve (anesthetized) and an increase in ATP hydrolysis (awake). (ii) Manganese-enhanced MRI transport rates: increased axonal transport rate. (iii) Functional connectivity: greater index of small-worldness.

Findings stated as awake relative to anesthetized (unless specified otherwise).

Iso. (isoflurane), med. (medetomidine), dexmed. (dexmedetomidine), SNR (signal-to-noise ratio), independent component analysis (ICA), s.c. (subcutaneous), MANCOVA (multivariate analysis of covariance), ROI (region-of-interest), LCN (lateral-cortical network), Hypo (hypothalamus), AUD-VIS (auditory–visual network), i.p. (intraperitoneal), CMRO2 (cerebral metabolic rate of oxygen), PET (positron emission tomography), BOLD (blood-oxygen-level-dependent), i.v. (intravenous), DMN (default mode network), PCr (phosphocreatine), Pi (inorganic phosphate), ATP (adenosine triphosphate).

Both anesthetized and awake mice show bilateral patterns of functional connectivity (Yoshida et al. 2016) alongside anatomically comparable networks (Gutierrez-Barragan et al. 2022). However, in awake relative to anesthetized mice, there are differences in network configuration, regional involvement, and organization (Yoshida et al. 2016; Fadel et al. 2022; Gutierrez-Barragan et al. 2022). Overall, increases in static functional connectivity are observed in awake relative to anesthetized mice (Yoshida et al. 2016; Jonckers et al. 2014; Desai et al. 2011; Tsurugizawa and Yoshimaru 2021; Gutierrez-Barragan et al. 2022), alongside shifts in dynamic functional connectivity (from negative to positive (Tsurugizawa and Yoshimaru 2021), or towards anti-coordination (Gutierrez-Barragan et al. 2022), in specific regions and networks) with awakening. Further, responses to stimulation are faster and stronger in target and off-target areas (Desai et al. 2011; Dinh et al. 2021), SNR is greater and less variable (Yoshida et al. 2016), and mice appear more like one another (Tsurugizawa and Yoshimaru 2021; Jonckers et al. 2014) when awake.

Despite some encouraging cross-study agreement, it is our opinion that more comparisons between awake and anesthetized brain states are needed before any broad recommendations can be made as to when a study should (or should not) be performed on awake or anesthetized animals. Especially when considering all the additional complexities involved in performing imaging experiments on awake animals (e.g. acclimation, surgical procedures, and stress). Improving our collective ability to choose when to implement an awake or anesthetized protocol will require more research that will be more fruitful if experimental strategies, data denoising, processing methods, and reporting practices, are more consistent across research groups. The purpose of this review is to facilitate this process which—in our view—is only just taking-off.

### Inter-species comparisons of fMRI data from awake and anesthetized conditions

Part of the motivation to overcome the challenges associated with performing fMRI experiments in awake mice is to obtain data that are a better match for data obtained in other species that are more typically imaged while awake (e.g. humans, and increasingly, non-human primates, and rats). The thinking is that by matching the state of consciousness across species the disparate use of anesthesia will not be the cause of any observations made across species. In the studies reviewed here, two find that observations made in awake mice are consistent with what has been seen in higher-order species (Xu et al. 2021; Gutierrez-Barragan et al. 2022) (Table 3). Other more recent work from Gutierrez-Barragan et al. has focused directly on inter-species (mouse, macaque, and human) brain organizational patterns in awake animals (Gutierrez-Barragan et al. 2023) and found similarities that might have been obscured had anesthesia been applied in only one species. Similarly, recent work in marmosets has directly compared anesthetized (isoflurane) and awake subjects and found global decreases in functional connectivity (measured with resting-state BOLD-fMRI) alongside altered interhemispheric and thalamic connectivity (Hori et al. 2020). These observations echo observations in awake and anesthetized mice (Jonckers et al. 2014; Tsurugizawa and Yoshimaru 2021) (Table 3). Yet, the overall spatial structure of more global brain network patterning observed in awake and anesthetized marmosets (Hori et al. 2020) was well preserved across states of consciousness. This second observation is also recapitulated in mice and is in-line with recent work which examines co-activation patterns across species (mice, macaque, and humans) (Mandino et al. 2022). In sum, there are emerging consistencies across species in the observed effects anesthesia has on brain-wide functional connectivity (e.g. a decrease in functional connectivity strength), but there are also consistencies across states of consciousness that are similar across species (e.g. gross network architecture). These initial findings affirm our sentiment above, that it will take time for concrete guidelines to emerge for when experiments in awake or anesthetized animals are warranted, and that there will likely be a place for both approaches (depending on the scientific question).

## How should we approach awake mouse fMRI going forward?

A handful of milestone studies have highlighted differences in brain measures linked to the type and dose of anesthesia, thus paving the way to the emergence of studies in awake mice (Grandjean et al. 2014; Bukhari et al. 2017; Wu et al. 2017; Steiner et al. 2020; 2021). Efforts to establish and standardize protocols on anesthetized animals can inform how we collectively approach awake mouse fMRI. Many best practices for conducting and reporting experiments in awake animals can be gleaned from studies in anesthetized mice and need to be more broadly observed.

### Reporting of exclusion criteria and number of animals

The reasons to exclude animals in an awake imaging study can differ from criteria applied to anesthetized animals, e.g. failure to acclimate to awake fMRI (established using measures of stress or motion), head-post failure, or imaging artifacts caused by head-posts. Instances of these and other adverse events (even if there are zero occurrences) must be reported. Further, it may be important to consider whether exclusion due to acclimation failure is selective (Sadaka et al. 2021). Animals that are more easily habituated may differ in other ways that could be problematic depending on the study. This may be particularly relevant for studies on disease or injury models (Ferris et al. 2014; Matsubayashi et al. 2018; Shofty et al. 2019; Asleh et al. 2020; Sakurai et al. 2020; Tsurugizawa et al. 2020). Possible interactions between stress and/or acclimation success should be systematically examined as part of future research. Further, no study to date has examined sex-based differences in acclimation success or study outcome. This is unsurprising given the overall lack of female animals (<5% of mice, and <9% of rats) in the awake rodent fMRI literature. Unfortunately, this is an established problem in imaging science more broadly that is too often “explained away” as a study limitation (Beery and Zucker 2011).

### Stress in animals

Animals in awake studies have been stressed and will bear both acute and chronic effects (McEwen 2007; Low et al. 2016; Han et al. 2019). To date, measurements of stress in awake mouse fMRI studies have been included to evaluate acclimation protocol success. Future work must also consider more completely how stress affects study outcome measures. However, a reliable and reproducible quantitative measure of stress in mice undergoing awake fMRI is still needed. There is limited evidence for any one existing measurement over another, and no clear benchmarks or targets. Measures of heart rate or corticosterone seem to provide some useful information but are cumbersome to obtain and there is substantial variance in the values that have been reported both in the awake mouse fMRI literature and beyond (Xie et al. 2013; Zhang et al. 2015; Cawthorn et al. 2016). These differences are likely due to both the study procedures (e.g. time allotted to recover from head-post surgery or acclimation protocol) as well as other “hidden” factors (e.g. housing conditions, institution, or experimenter) which are hard to identify and control. There is a pressing need for more comprehensive studies that explicitly examine how to quantitatively measure stress in mice undergoing fMRI while awake. Looking ahead, establishing benchmarks for measuring stress will aid in the standardization of practices, which will help with cross-laboratory comparisons, and improve intra-lab reproducibility. Importantly, until better standards are established, the use of appropriate control groups will be key for disentangling the contributions of stress and motion from the study question. It should also be noted that all fMRI studies include some level of stress (in any species) and, perhaps, regardless of the use of anesthesia (Urosevic et al. 2022a, 2022b).

### All animals move, awake animals move more

Cut-offs for mouse, session, scan/run, and frame exclusion must be reported alongside average motion outcomes and amount of data discarded (for all groups). Quantifying the contribution of motion to study outcome can guide researchers toward standardized methods for reporting, ultimately facilitating inter-study comparisons. Currently, there is substantial variety in how motion is reported (or illustrated) making inter-study comparisons challenging. At this junction, reporting the average and SD of the six-motion-parameter estimates and FD is desirable. This does not preclude additional ways of plotting or summarizing the data. Further, as experiments become more complex and start to incorporate more tasks (a major goal in the field that some have pioneered [Harris et al. 2015; Han et al. 2019; Sakurai et al. 2020; Cover et al. 2021]) the need to uncouple motion from measures of brain activity will become increasingly important. Finally, evidence from in-scanner videography (Desjardins et al. 2019) and the two longest acclimation protocols (Gutierrez-Barragan et al. 2022; Zeng et al. 2022) suggest that there is a limit to how still mice will be during an awake imaging session. This is unsurprising given that a calm subject (including a human) will engage in a range of movements during a typical imaging timeframe.

### What we need to keep in mind to move forward

Awake mouse fMRI experiments are inherently more involved, expensive, and stressful (for the mice and experimenters) than the anesthetized counterparts. As such, it is incumbent that studies demonstrate that collecting the data while animals are awake is necessary. This means addressing why an experiment needed to be performed on awake animals and quantifying the benefits. To date, most studies fall short in this regard. This is not a dispute of there being effects of anesthesia on the brain or the validity of any of the commonly cited reasons for performing experiments on awake subjects. Instead, it is a call for a more rigorous effort to place outcome measures from awake mouse fMRI experiments in context. A study is not inherently “better” if performed using awake animals.

The continued development of successful acclimation protocols for awake mouse fMRI stands to have a profound effect on our understanding of brain function in health and disease. Yet, establishing generalizable protocols will take significant investment. We acknowledge that many of the suggestions we make increase the time and monetary requirements of a study (e.g. inclusion of both sexes, more control groups, the use of head-post implants, longer acclimation protocols that include time in the real-scanner, etc.). Improving data sharing practices (Table 1), also comes at a very real cost, but stands to help the scientific community progress more quickly through cross-institute research and data re-use. For the field of awake mouse fMRI to develop efficiently and fruitfully, increasing support for preclinical research, and more data sharing, will be crucial.

## Conclusion

Thirty studies which collect awake mouse fMRI data, identified by a systematic literature search, have been comprehensively reviewed. Wherever feasible, we have compared quantitative measures across studies and drawn general conclusions to accelerate development in the field. A similar summary of the awake rat fMRI literature is also included for context, given the influence this work has had on awake mouse fMRI. We have included broad recommendations for practices that show evidence for producing favorable results, and highlighted areas where further development is needed to address gaps in knowledge. This resource will favor cross-study comparisons and facilitate the development and standardization of robust awake mouse fMRI experiments that stand to have a wide-ranging impact on neuroimaging and neuroscience.

## Supplementary Material

Supplement_with_changes_R2_bhad478
