# Supplementary material for: Where do we stand on fMRI in awake mice?
Source: Cereb Cortex. 2023 Dec 13;34(1):bhad478. doi: 10.1093/cercor/bhad478 (PMC10793583; doi:10.1093/cercor/bhad478)
Supplement: Supplement_with_changes_R2_bhad478 [file supplement_with_changes_r2_bhad478.docx]

**Supplementary Information**

**Supplementary Tables**

1. Table S1. Queries used to identify articles within the scope of this review.
2. Table S2. Full list of articles on mice included in this review.
3. Table S3. Head- and body-restraint approaches.
4. Table S4. Descriptions of mock-scanner environments.
5. Table S5. Time of day for training/imaging.
6. Table S6. Measures of animal stress.
7. Table S7. Data acquisition hardware and sequence parameters.
8. Table S8. Data preprocessing and exclusion criteria based on motion.
9. Table S9. Exclusions for motion with 6-parameter motion outcome measures.
10. Table S10. Exclusions for motion with framewise displacement outcome measures.
11. Table S11. Exclusion criteria other than motion.
12. Table S12. Full list of articles on rats included in this review.
13. Table S13. Summary of articles on rats in Group 1a (above the timeline).
14. Table S14. Summary of articles on rats in Group 1b (above the timeline).
15. Table S15. Summary of articles on rats in Groups 2-12 (below the timeline).

**Table S1.** **Queries used to identify articles within the scope of this review.**

| **Database** | **Species** | **Query statement** |
| --- | --- | --- |
| BioRxiv | Mouse | Words appearing anywhere in the title, abstract, or full text: “Awake, Mice, fMRI, BOLD, unanesthetized.” |
| ScienceDirect | Mouse | Find articles with these terms: “(awake OR unanesthetized) AND (mouse OR mice OR murine) AND (fMRI OR BOLD OR “blood oxygen level dependent”)” Title, abstract or author-specified keywords: “(awake or unanesthetized) |
| PubMed | Mouse | ("functional magnetic resonance imaging"[All Fields] OR "fMRI"[All Fields] OR "BOLD"[All Fields] OR "blood oxygen level dependent"[All Fields]) AND ("mice"[All Fields] OR "mouse"[All Fields] OR "murine"[All Fields]) AND ("awake"[All Fields] OR "wake"[All Fields] OR "unanesthetized"[All Fields]) |
| PubMed | Rat | ("functional magnetic resonance imaging"[All Fields] OR "fMRI"[All Fields] OR "BOLD"[All Fields] OR "blood oxygen level dependent"[All Fields]) AND ("rat"[All Fields] OR "rats"[All Fields] OR "murine"[All Fields]) AND ("awake"[All Fields] OR "wake"[All Fields] OR "unanesthetized"[All Fields]) |

**Table S2.** **Full list of articles on mice included in this review.**

| **#** | **Study** | **Full Reference** |
| --- | --- | --- |
| **1** | **Desai 2011** | Desai M, Kahn I, Knoblich U, Bernstein J, Atallah H, Yang A, Kopell N, Buckner RL, Graybiel AM, Moore CI, Boyden ES. Mapping brain networks in awake mice using combined optical neural control and fMRI. J Neurophysiol. 2011 Mar;105(3):1393-405. |
| **2** | **Bergmann 2016** | Bergmann E, Zur G, Bershadsky G, Kahn I. The Organization of Mouse and Human Cortico-Hippocampal Networks Estimated by Intrinsic Functional Connectivity. Cereb Cortex. 2016 Dec;26(12):4497-4512. doi: 10.1093/cercor/bhw327. Epub 2016 Oct 25. |
| **3** | **Shofty 2019** | Shofty B, Bergmann E, Zur G, Asleh J, Bosak N, Kavushansky A, Castellanos FX, Ben-Sira L, Packer RJ, Vezina GL, Constantini S, Acosta MT, Kahn I. Autism-associated Nf1 deficiency disrupts corticocortical and corticostriatal functional connectivity in human and mouse. Neurobiol Dis. 2019 Oct;130:104479. |
| **4** | **Asleh 2020** | Asleh J, Shofty B, Cohen N, Kavushansky A, López-Juárez A, Constantini S, Ratner N, Kahn I. Brain-wide structural and functional disruption in mice with oligodendrocyte-specific *Nf1* deletion is rescued by inhibition of nitric oxide synthase. Proc Natl Acad Sci U S A. 2020 Sep 8;117(36):22506-22513. |
| **5** | **Bergmann 2020** | Bergmann E, Gofman X, Kavushansky A, Kahn I. Individual variability in functional connectivity architecture of the mouse brain. Commun Biol. 2020 Dec 4;3(1):738. |
| **6** | **Ferris 2014** | Ferris CF, Kulkarni P, Toddes S, Yee J, Kenkel W, Nedelman M. Studies on the Q175 Knock-in Model of Huntington's Disease Using Functional Imaging in Awake Mice: Evidence of Olfactory Dysfunction. Front Neurol. 2014 Jun 30;5:94. |
| **7** | **Moore 2016** | Moore K, Madularu D, Iriah S, Yee JR, Kulkarni P, Darcq E, Kieffer BL, Ferris CF. BOLD Imaging in Awake Wild-Type and Mu-Opioid Receptor Knock-Out Mice Reveals On-Target Activation Maps in Response to Oxycodone. Front Neurosci. 2016 Nov 3;10:471. |
| **8** | **Madularu 2017^a^** | Madularu D, Mathieu AP, Kumaragamage C, Reynolds LM, Near J, Flores C, Rajah MN. A non-invasive restraining system for awake mouse imaging. J Neurosci Methods. 2017 Aug 1;287:53-57. |
| **9** | **Farra 2020** | Farra YM, Eden MJ, Coleman JR, Kulkarni P, Ferris CF, Oakes JM, Bellini C. Acute neuroradiological, behavioral, and physiological effects of nose-only exposure to vaporized cannabis in C57BL/6 mice. Inhal Toxicol. 2020 Apr;32(5):200-217. |
| **10** | **Sadaka 2021** | Sadaka AH, Ozuna AG, Ortiz RJ, Kulkarni P, Johnson CT, Bradshaw HB, Cushing BS, Li AL, Hohmann AG, Ferris CF. Cannabidiol has a unique effect on global brain activity: a pharmacological, functional MRI study in awake mice. J Transl Med. 2021 May 24;19(1):220. |
| **11** | **Tsurugizawa 2013** | Tsurugizawa T, Nogusa Y, Ando Y, Uneyama H. Different TRPV1-mediated brain responses to intragastric infusion of capsaicin and capsiate. Eur J Neurosci. 2013 Dec;38(11):3628-35. doi: 10.1111/ejn.12365. Epub 2013 Sep 18. PMID: 24102723. |
| **12** | **Yoshida 2016** | Yoshida K, Mimura Y, Ishihara R, Nishida H, Komaki Y, Minakuchi T, Tsurugizawa T, Mimura M, Okano H, Tanaka KF, Takata N. Physiological effects of a habituation procedure for functional MRI in awake mice using a cryogenic radiofrequency probe. J Neurosci Methods. 2016 Dec 1;274:38-48. |
| **13** | **Matsubayashi 2018** | Matsubayashi K, Nagoshi N, Komaki Y, Kojima K, Shinozaki M, Tsuji O, Iwanami A, Ishihara R, Takata N, Matsumoto M, Mimura M, Okano H, Nakamura M. Assessing cortical plasticity after spinal cord injury by using resting-state functional magnetic resonance imaging in awake adult mice. Sci Rep. 2018 Sep 26;8(1):14406. |
| **14** | **Takata 2018** | Takata N, Sugiura Y, Yoshida K, Koizumi M, Hiroshi N, Honda K, Yano R, Komaki Y, Matsui K, Suematsu M, Mimura M, Okano H, Tanaka KF. Optogenetic astrocyte activation evokes BOLD fMRI response with oxygen consumption without neuronal activity modulation. Glia. 2018 Sep;66(9):2013-2023. |
| **15** | **Tsurugizawa 2020** | Tsurugizawa T, Tamada K, Ono N, Karakawa S, Kodama Y, Debacker C, Hata J, Okano H, Kitamura A, Zalesky A, Takumi T. Awake functional MRI detects neural circuit dysfunction in a mouse model of autism. Sci Adv. 2020 Feb 5;6(6):eaav4520. |
| **16** | **Takata 2021** | Takata N, Sato N, Komaki Y, Okano H, Tanaka KF. Flexible annotation atlas of the mouse brain: combining and dividing brain structures of the Allen Brain Atlas while maintaining anatomical hierarchy. Sci Rep. 2021 Mar 18;11(1):6234. |
| **17** | **Abe 2021** | Abe Y, Kwon S, Oishi M, Unekawa M, Takata N, Seki F, Koyama R, Abe M, Sakimura K, Masamoto K, Tomita Y, Okano H, Mushiake H, Tanaka KF. Optical manipulation of local cerebral blood flow in the deep brain of freely moving mice. Cell Rep. 2021 Jul 27;36(4):109427. |
| **18** | **Tsurugizawa 2021^a+,b^** | Tsurugizawa T, Tamada K, Debacker C, Zalesky A, Takumi T. Cranioplastic Surgery and Acclimation Training for Awake Mouse fMRI. Bio Protoc. 2021 Apr 5;11(7):e3972.  Tsurugizawa T, Yoshimaru D. Impact of anesthesia on static and dynamic functional connectivity in mice. Neuroimage. 2021 Nov 1;241:118413. doi: 10.1016/j.neuroimage.2021.118413. Epub 2021 Jul 20. PMID: 34293463. |
| **19** | **Han 2019** | Han Z, Chen W, Chen X, Zhang K, Tong C, Zhang X, Li CT, Liang Z. Awake and behaving mouse fMRI during Go/No-Go task. Neuroimage. 2019 Mar;188:733-742. |
| **20** | **Chen 2020** | Chen X, Tong C, Han Z, Zhang K, Bo B, Feng Y, Liang Z. Sensory evoked fMRI paradigms in awake mice. Neuroimage. 2020 Jan 1;204:116242. |
| **21** | **Xu 2021** | Xu M, Bo B, Pei M, Chen Y, Shu CY, Qin Q, Hirschler L, Warnking JM, Barbier EL, Wei Z, Lu H, Herman P, Hyder F, Liu ZJ, Liang Z, Thompson GJ. High-resolution relaxometry-based calibrated fMRI in murine brain: Metabolic differences between awake and anesthetized states. J Cereb Blood Flow Metab. 2021 Dec 15:271678X211062279. |
| **22** | **Jonkers 2014** | Jonckers E, Delgado y Palacios R, Shah D, Guglielmetti C, Verhoye M, Van der Linden A. Different anesthesia regimes modulate the functional connectivity outcome in mice. Magn Reson Med. 2014 Oct;72(4):1103-12. |
| **23** | **Harris 2015** | Harris AP, Lennen RJ, Marshall I, Jansen MA, Pernet CR, Brydges NM, Duguid IC, Holmes MC. Imaging learned fear circuitry in awake mice using fMRI. Eur J Neurosci. 2015 Sep;42(5):2125-34. |
| **24** | **Desjardins 2019** | Desjardins M, Kılıç K, Thunemann M, Mateo C, Holland D, Ferri CGL, Cremonesi JA, Li B, Cheng Q, Weldy KL, Saisan PA, Kleinfeld D, Komiyama T, Liu TT, Bussell R, Wong EC, Scadeng M, Dunn AK, Boas DA, Sakadžić S, Mandeville JB, Buxton RB, Dale AM, Devor A. Awake Mouse Imaging: From Two-Photon Microscopy to Blood Oxygen Level-Dependent Functional Magnetic Resonance Imaging. Biol Psychiatry Cogn Neurosci Neuroimaging. 2019 Jun;4(6):533-542. |
| **25** | **Sakurai 2020** | Sakurai K, Shintani T, Jomura N, Matsuda T, Sumiyoshi A, Hisatsune T. Hyper BOLD Activation in Dorsal Raphe Nucleus of APP/PS1 Alzheimer's Disease Mouse during Reward-Oriented Drinking Test under Thirsty Conditions. Sci Rep. 2020 Mar 3;10(1):3915. |
| **26** | **Cover 2021** | Cover CG, Kesner AJ, Ukani S, Stein EA, Ikemoto S, Yang Y, Lu H. Whole brain dynamics during optogenetic self-stimulation of the medial prefrontal cortex in mice. Commun Biol. 2021 Jan 14;4(1):66. doi: 10.1038/s42003-020-01612-x. PMID: 33446857; PMCID: PMC7809041. |
| **27** | **Dinh 2021** | Dinh TNA, Jung WB, Shim HJ, Kim SG. Characteristics of fMRI responses to visual stimulation in anesthetized vs. awake mice. Neuroimage. 2021 Feb 1;226:117542. |
| **28** | **Gutierrez-Barragan 2022** | Gutierrez-Barragan D, Singh NA, Alvino FG, Coletta L, Rocchi F, De Guzman E, Galbusera A, Uboldi M, Panzeri S, Gozzi A. Unique spatiotemporal fMRI dynamics in the awake mouse brain. Curr Biol. 2022 Feb 7;32(3):631-644.e6. |
| **29** | **Zeng 2022** | Zeng H, Jiang Y, Beer-Hammer S, Yu X. Awake Mouse fMRI and Pupillary Recordings in the Ultra-High Magnetic Field. Front Neurosci. 2022 Jul 6;16:886709. doi: 10.3389/fnins.2022.886709. PMID: 35903811; PMCID: PMC9318598. |
| **30** | **Fadel 2022** | Fadel LC, Patel IV, Romero J, Tan IC, Kesler SR, Rao V, Subasinghe SAAS, Ray RS, Yustein JT, Allen MJ, Gibson BW, Verlinden JJ, Fayn S, Ruggiero N, Ortiz C, Hipskind E, Feng A, Iheanacho C, Wang A, Pautler RG. A Mouse Holder for Awake Functional Imaging in Unanesthetized Mice: Applications in ^31^P Spectroscopy, Manganese-Enhanced Magnetic Resonance Imaging Studies, and Resting-State Functional Magnetic Resonance Imaging. Biosensors (Basel). 2022 Aug 8;12(8):616. doi: 10.3390/bios12080616. PMID: 36005011; PMCID: PMC9406174. |

**+** protocol article referring to Tsurugizawa 2020, not included in the analyses

**Table S3.** **Head- and body-restraint approaches.**

| **Study** | **Use Anes.** | **Head-restraint** | **Body-restraint** |
| --- | --- | --- | --- |
| **Surgically implanted head-post reliant systems** | | | |
| **Desai 2011** | No | Head-post with fixation posterior to mouse (**Figure 2a**) | Cylindrical tube, ‘falcon tube’, 4.5cm diameter, enclosed at tail-end with padded material inside to reduce large movements |
| **Bergmann 2016, 2020; Shofty 2019; Asleh 2020** | Yes |  |  |
| **Han 2019; Chen 2020** | No |  | No description besides closed tubular enclosure appearing in figures showing the apparatus |
| **Xu 2021** | Unk |  |  |
| **Desjardins 2019** | Yes |  | Suspended bed, body/limb movement unrestricted |
| **Dinh 2021** | Yes | Head-post with fixation points on right and left side (**Figure 2b**) | Body and neck movement restricted within box with holes in the base for free limb movement |
| **Sakurai 2020** | Unk |  | Sled-like bed depicted to which head-post is mounted, no covering of the body |
| **Gutierrez-Barragan 2022** | No |  | Tape is used to secure the back and tail, limbs are left free, cotton as placed under the jaw to reduce movement of jaw and forelimbs |
| **Yoshida 2016; Matsubayashi 2018; Takata 2018,** | No | Head-post with fixation point anterior to the skull (**Figure 2c**, left) | Sled-like bed to which the head-post is mounted with gauze taped across the back |
| **Abe 2021** | Yes |  |  |
| **Tsurugizawa 2013, 2020, 2021^b^** | Yes | Head-post with fixation point directly above the skull (**Figure 2c**, right) | Box which restricts large movements lined with cotton material |
| **Cover 2021** | No |  | Plastic tube which restricts large movements with paper tissue lining |
| **Zeng 2022** | No |  | Box which restricts large movements depicted in figures |
| **Systems that do not use surgically implanted head-posts** | | | |
| **Ferris 2014; Moore 2016; Farra 2020; Sadaka 2021** | Yes | Cushion to support the head and an incisor bar | Tube which surrounds the body and restricts large movements, bar placed around the neck to restrict head and forelimb movement |
| **Madularu 2017^a^** | Yes | Adjustable cushion | Adjustable plates on either side of the body |
| **Jonkers 2014** | Yes | Ear bars with numbing agent applied | Not discussed |
| **Harris 2015** | Yes | Animal Research, LLC, USA* | Limbs and body secured with tape, cylindrical tube fit over body, as well as shoulder bar |
| **Fadel 2022** | Yes | Incisor bar with nose cone as well as cheek support that is built into the neck holder | Sled design, gauze is inserted on the sides of the head and tape is used to secure the limbs and tail; gauze is then wrapped and taped around the whole animal |

* Commercially available device

**Table S4.** **Descriptions of mock-scanner environments.**

|  | **Study** | **Description of the mock-sys.** | **Figure** |
| --- | --- | --- | --- |
|  | **Ferris 2014** | Black box with recording of MRI noise | ✖️ |
|  | **Moore 2016** |  | ✖️ |
|  | **Sadaka 2021** |  | ✖️ |
|  | **Madularu 2017^a^** | Enclosed space with loudspeaker (~87dB) | ✔️ |
|  | **Farra 2020** | Enclosed black box with audio speakers | ✖️ |
| **Tsurugizawa 2013** | | Non-magnetic bore and head positioner | ✖️ |
| **Tsurugizawa 2020** | |  | ✖️ |
| **Tsurugizawa 2021^b^** | |  | ✔️ |
| **Yoshida 2016** | | Mock fMRI environment including bed, holder, and coil with noise (100dB) within a soundproof box | ✔️ |
| **Matsubayashi 2018** | |  | ✖️ |
| **Takata 2018** | |  | ✖️ |
| **Takata 2021** | | Mock fMRI environment | ✖️ |
| **Abe 2021** | | Mock fMRI environment with noise (100dB) | ✖️ |
| **Harris 2015** | | Mock scanner with mock scans | ✖️ |
| **Desjardins 2019** | | Mock MRI scanner bore | ✔️ |
| **Cover 2021** | | Mock scanner bore with noise (115dB) | ✖️ |
| **Dinh 2021** | | Mock scanner bore with noise (110-120dB) | ✔️ |
| **Gutierrez-Barragan 2022** | | Bed and mock coil with noise equivalent to the real scanner | ✔️ |
| **Fadel 2022** | | Bore and chamber (dimensions included) with speakers inclusive of gas delivery system | ✔️ |

**Table S5.** **Time of day for training/imaging.**

| **Study** | **Scan time** | **Light cycle** |
| --- | --- | --- |
| **Desai 2011** | 14:00 | Unknown |
| **Bergmann 2016** | Late afternoon | Reversed |
| **Tsurugizawa 2013** | 10:00-17:00 | Not reversed |
| **Yoshida 2016** | 13:00-15:00 | Not reversed |
| **Tsurugizawa 2020** | 10:00-18:00 | Not reversed |
| **Tsurugizawa 2021^b^** | 10:00-18:00 | Unknown |
| **Dinh 2021** | 13:00 | Unknown |
| **Gutierrez-Barragan 2022** | 9:00-12:00 | Not reversed |

**Table S6.** **Measures of animal stress.**

| **Measurement** | **Study** | **Outcome** |
| --- | --- | --- |
| **Qualitative measures of stress** | | |
| Behavior: struggling, eye secretions, excessive vocalization, changes in breathing patterns | **Desai 2011; Bergmann 2016; Zeng 2022** | Evidence of stress signs are reduced with acclimation |
| Task performance | **Han 2019;**  **Cover 2021** | Mice perform task in-scanner indicating low levels of stress |
| Facial movements | **Desjardins 2019** | Camera displaying mouse face during scanning |
| **Quantitative measures of stress** | | |
| Motion in fMRI data | **Ferris 2014; Sadaka 2021** | Low average group motion |
| Respiration rate  (**Figure 3a**) | **Madularu 2017^a^** | Reduced on acclimation days 2-5 (~180 breaths/min) and during data acquisition relative to day 1 (~200 breaths/min) (N=5) |
|  | **Tsurugizawa 2013** | Normal on acclimation day 4 (181 ± 8 breaths/min)  (mean ± standard error, SE) (N=18) |
|  | **Matsubayashi 2018** | Not reported |
|  | **Tsurugizawa 2020** | Stable on acclimation days 1-4 (~180-200 breaths/min) (N=15) |
|  | **Tsurugizawa 2021^b^** | Normal range |
|  | **Chen 2020** | (~300 breaths/min) during data acquisition (N=40) |
|  | **Xu 2021** | (262-293 breaths/min) during data acquisition (N=15) |
|  | **Jonckers 2014** | (320 ± 20 breaths/min) during data acquisition |
|  | **Harris 2015** | No difference between 5-day (253 ± 26 breaths/min) (mean ± SD) and 12-day acclimation protocol (206 ± 60 breaths/min) (N=3/4) |
|  | **Fadel 2022** | Not reported |
|  | **Dinh 2021** | (260-300 breaths/min) during data acquisition (N=6) |
| Heart rate  (**Figure 3b**) | **Tsurugizawa 2013** | Normal on acclimation day 4 (478 ± 48 beats/min) (mean ± SE) (N=18) |
|  | **Yoshida 2016** | Elevated on acclimation days 1-6 (630-670 beats/min), reduced on acclimation days 7 & 8 (600-620 beats/min) (N=7) |
|  | **Tsurugizawa 2020** | Elevated on acclimation day 1 (~525 beats/min), reduced on acclimation days 3 & 4 (~450 beats/min) (N=15) |
|  | **Fadel 2022** | Not reported |
| Fecal boli | **Madularu 2017^a^** | No change (2.5 boli/day) (N=5) |
|  | **Fadel 2022** | Not reported |
|  | **Zeng 2022** | <10 (N=6) |
| Fecal weight | **Yoshida 2016** | Elevated in the acclimated (N=7), relative to control (N=9) group, for acclimation days 1-5. Not different from controls on acclimation days 6 and 7 |
| Body weight | **Yoshida 2016** | Equivalent in acclimated (N=7) and control (N=9) mice and increasing in time |
|  | **Harris 2015** | Greater with 5-day (-2.1 ± 0.4g) (mean ± SD) relative to 12-day acclimation protocol (-0.8 ± 0.3g) (N=3/4) |
|  | **Gutierrez-Barragan 2022** | Acclimated and control (naïve) groups show similar weight gain trajectories and no weight loss (N=7 per group) |
| EMG power | **Yoshida 2016** | Reduced on acclimation day 2-8 relative to day 1 (N=9) |
| Corticosterone  (**Figure 3c**) | **Tsurugizawa 2020** | Elevated on acclimation day 1 (~400 ng/ml) (N=4) relative to control (~190 ng/ml) (N=5), and acclimation day 4 (~200 ng/ml) (N=5) |
|  | **Harris 2015** | Elevated with 5-day (527 ± 163 nmol/L, or ~191 ng/ml) (mean ± SD) relative to 12-day acclimation protocol (191 ± 88 nmol/L, or 69 ng/ml) (N=3/4) – values converted from nmol/L |
|  | **Dinh 2021** | Control (acclimation naïve) (~10 ng/ml) (N=2), day 5 (~30 ng/ml), and day 10 (~17 ng/ml) (with only mock-scanner training) |
|  | **Gutierrez-Barragan 2022** | Elevated in acclimated group after handling or immobilization acclimation (~100-250 ng/ml) relative to control (20-200 ng/ml), yet equivalent to circadian excursion (150-200 ng/ml) and substantially less than acute restraint (350-1100 ng/ml) |
| Cortisol ***** | **Fadel 2022** | Control (acclimation naïve) (0.46 ng/ml) (N=10), acclimation day 1 (4.1 ng/ml) (N=6), and acclimation day 4 (1.6 ng/ml) (N=6) |
| Body movement | **Harris 2015** | More frequent with 5-day (18 ± 12) (mean ± SD) relative to 12-day acclimation protocol (5 ± 3) (N=3/4) |
| Pupil dynamics | **Zeng 2022** | Reduced median diameter between week 1 (56 pixels) and week 8 (35 pixels) of acclimation (N=6) |
| Chest movement | **Zeng 2022** | Variance decreases between week 1 and > 8 of acclimation (N=6) |
| **Prophylactic practices beyond acclimation training meant to reduce stress** | | |
| Edible Buprenorphine gel | **Bergmann 2016** | Reduces pain and speeds recovery following head-post surgery |
| High-energy chow | **Abe 2021** | Facilitates recovery of bodyweight after surgery |
| Earplugs | **Tsurugizawa 2013; 2020; 2021^b^; Desjardins 2019** | Used to reduce perception of scanner noise |
| EMLA cream | **Jonckers 2014** | To reduce physical pain from ear bars (used for immobilization) |

^♦^Animals were excluded based on physiological parameters above the acceptable range.

* Cortisol is the primary endogenous adrenal steroid in humans. Corticosterone is the equivalent in rodents (Raff et al. 2016). Thus, corticosterone (not cortisol) is the more appropriate measurement.

**Table S7.** **Data acquisition hardware and sequence parameters.**

| **Study** | **B_0_** | **Coil** | **Sequence** | **TR (s)** | **Voxel size (mm)** | **Slices** | **Data / session** | |
| --- | --- | --- | --- | --- | --- | --- | --- | --- |
| **Desai 2011** | 9.4T | Custom receiver-only 20mm loop | SE and GE***** EPI | 2.50 | 0.150 x 0.150 x 0.50 | 5 | ~8 mins x 4 runs | |
| **Bergmann 2016 Bergmann 2020**  **Shofty 2019**  **Asleh 2020** |  | Bruker  receive-only 20mm loop | SE-EPI |  | 0.150 x 0.150 x 0.45  (Whole brain) | 30 |  |  |
| **Ferris 2014** | 7.0T | Bruker quadrature transmit/receive | HASTE | 6.00 | 0.260 x 0.260 x 0.75  (Whole brain) | 20 | 5 or 7 mins | |
| **Farra 2020**  **Sadaka 2021** |  |  |  |  | 0.188 x 0.188 x 0.75  (Whole brain) | 18 | 15 mins | |
| **Moore 2016** |  |  | FSE/RAREst |  | 0.312 x 0.312 x 1.00  (Whole brain) | 22 | 20 mins | |
| **Madularu 2017^a^** |  | Bruker quadrature receive-only | RAREst | 3.00 | 0.300 x 0.300 x 1.20  (Whole brain) | 22 | 10 mins x 2 runs | |
| **Tsurugizawa 2013** | 4.7T | Custom 3cm volume coil | T_2_* weighted FLASH | 15.00 | 0.385 x 0.385 x 1.00  (Whole brain) | 15 | 40 mins | |
| **Tsurugizawa 2020** |  |  | T_2_* weighted EPI | 2.00 | 0.200 x 0.200 x 1.00  (No cerebellum/bulb) |  | 11 & 11.5 mins  (2 runs total) | |
| **Tsurugizawa 2021^b^** | 11.7T | CryoProbe, Bruker | GE-EPI | 1.50 | 0.100 x 0.100 x 0.50  (Whole brain) |  | 10 mins | |
| **Yoshida 2016** | 7.0T | CryoProbe, Bruker | GE-EPI | 1.50 | 0.200 x 0.200 x 0.50  (No cerebellum/bulb) | 18 | 5 mins | |
| **Takata 2018** |  |  |  |  |  |  | 8.5 mins | |
| **Takata 2021** |  |  |  |  | 0.200 x 0.200 x 0.75  (Whole brain) | 18^☨^ | 10 mins x 2 runs | |
| **Matsubayashi 2018** |  |  |  | 1.00 | 0.200 x 0.200 x 0.50  (No cerebellum/bulb) | 16 | 10 mins | |
| **Abe 2021** |  |  | GE-EPI Enhanced^♦^ | 2.00 | 0.313 x 0.313 x 0.75  (Whole brain) | 20^☨^ | 7 mins | |
| **Han 2019** | 9.4T | Cryogenic phased array, Bruker | EPI | 1.50 | 0.150 x 0.150 x 0.40  (Whole brain) | 22 | 6 mins | |
| **Chen 2020** |  |  | EPI | 1.50 | 0.150 x 0.150 x 0.40 | 15 | ~6 mins | x 5 runs |
|  |  |  |  |  | 0.150 x 0.150 x 0.30 | 12 | ~8 mins |  |
|  |  |  |  | 0.35 | 0.200 x 0.200 x 0.40 | 10 | ~6 mins | x 5 runs |
|  |  |  |  |  | 0.200 x 0.200 x 0.30 |  | ~8 mins |  |
| **Xu 2021** |  |  | pCASL | 4.00 | 0.225 x 0.255 x 0.50 | 20 | ~10 mins | |
| **Jonkers 2014** | 9.4T | Bruker  receive-only 20mm loop | GE-EPI | 2.00 | 0.160 x 0.310 x 0.40  (Whole brain) | 16^☨^ | 5 mins | |
| **Cover 2021** |  |  |  | 1.00 | 0.260 x 0.260 x 0.60  (No cerebellum/bulb) | 15 | 10 mins x 2-3 runs on 2 days | |
| **Dinh 2021** |  | 10mm surface coil |  |  | 0.156 x 0.156 x 0.50 | 9 | 2 mins x 2 runs | |
| **Fadel 2022** |  | Unclear | SE-EPI | 1.20 | 0.250 x 0.250 x 0.60 | Unclear | 12 mins | |
| **Harris 2015** | 7.0T | Agilent, Quadrature receive/transmit | SE-EPI | 2.50 | 0.300 x 0.300 x 0.80  (No cerebellum/bulb) | 16 | ~17 mins | |
| **Desjardins 2019** |  | Custom 2x3cm surface coil | GE-EPI | 1.00 | 0.200 x 0.200 x 1.00 | 5 | Unclear | |
| **Gutierrez-Barragan 2022** |  | Four channel receiver coil | EPI |  | 0.230 x 0.230 x 0.60  (Whole brain) | 18 | 32 mins | |
| **Sakurai 2020** | 14T | Custom surface coil | SE-EPI | 2.00 | 0.269 x 0.269 x 0.75 | 6 | 3.5 mins | |
| **Zeng 2022** |  |  | EPI | 1.00 | 0.300 x 0.300 x 0.50  (Whole brain) | 24 | 3.5 mins | |

***** SE and GE were compared, and SE found to have fewer susceptibility artifacts.

^♦^ Intravenous ultrasmall superparamagnetic iron administered prior to data collection.

^☨^ Slice gap (0.05mm (Takata et al. 2021; Abe et al. 2021), or 0.1mm (Jonkers et al. 2014)).

Voxel dimensions are listed as: a x b, in-plane, x c, through-plane.

If coverage is of the whole brain, or most of the brain (excluding the olfactory bulb and cerebellum), this is noted below the voxel dimensions. Otherwise, coverage is limited to a region of interest (ROI).

TR (repetition time), SE (spin echo), GE (Gradient Echo), EPI (Echo Planar Imaging), HASTE (Half Fourier Acquisition Signal Shot Turbo Spin Echo), FSE/RAREst (Fast Spin Echo/Rapid Acquisition with Relaxation Enhancement), FLASH (Fast Low Angle Shot), pCASL (pseudo-Continuous Arterial Spin Labeling).

**Table S8.** **Data preprocessing and exclusion criteria based on motion.**

|  | **Study** | **Software** | **Regression** | **Filter** | **Smooth** | **Motion exclusion criteria** | |
| --- | --- | --- | --- | --- | --- | --- | --- |
|  |  |  |  |  |  | Frame | Run, session, trial |
|  | **Desai 2011** | AFNI | Unspecified | NA | NA | NA | NA |
|  | **Shofty 2019** | Unspecified | Motion & 1^st^ derivatives, demean, detrend, CSF, GS | 0.009 < *f* < 0.08 | 600µm | FD>0.05mm, DVARS 150% IQR >75^th^, inclusive of 1 frame after | Runs <50/200 frames & sessions <300/800 frames |
|  | **Asleh 2020** | ANTs | **Shofty 2019** & vascular |  |  |  |  |
|  | **Bergmann 2016** | Unspecified | Motion, demean, detrend, CSF, WM |  | 450µm | FD>0.05mm, DVARS of 0.5%, inclusive of 2 frames before & 1 after | Sessions <7/33mins |
|  | **Bergmann 2020** | SPM2, FSL, MATLAB | **Bergmann 2016** & 1^st^ temporal derivatives |  |  | **Shofty 2019** | Runs <50/200 frames & sessions <192/800 frames |
|  | **Madularu 2017^a^** | FSL, MATLAB | NA | NA | NA | >0.15mm in X & Y, >0.6mm in Z | NA |
|  | **Ferris 2014** | Unknown |  |  |  | NA |  |
|  | **Moore 2016** | SPM8 |  |  | 800µm |  |  |
|  | **Farra 2020** |  |  |  |  |  |  |
|  | **Sadaka 2021** | SPM12, AFNI, FSL, MATLAB | Motion, CSF, WM | 0.01 < *f* < 0.1 |  | MAD censoring, unspecified | Motion, unspecified |
|  | **Tsurugizawa 2013** | SPM8, MATLAB, MRIcron | NA | NA | NA | NA | Motion, unspecified |
|  | **Yoshida 2016** | SPM12, GIFT, Icasso, MATLAB, SPSS |  | 0.01 < *f* < 0.1 | 400µm |  | NA |
|  | **Takata 2018** | SM12 |  |  |  |  |  |
|  | **Tsurugizawa 2020** | SPM8, ICA-AROMA, FSL | FD, detrend, CSF, WM, GS |  | 600µm |  |  |
|  | **Tsurugizawa 2021^b^** | SPM12, CONN, MATLAB | Motion, detrend, CSF, WM | 0.01 < *f* < 0.08 | 0.15 x 0.15 mm^2^ / pixel |  |  |
| **Matsubayashi 2018** | | SPM12, MATLAB, CONN, SPSS | NA | 0.009 < *f* < 0.1 | 600µm |  |  |
| **Abe 2021** | | SPM12, MATLAB |  | NA |  |  |  |
| **Takata 2021** | | ANTs, SPM12, CONN, MATLAB | Motion, detrend, CSF, WM | 0.01 < *f* < 0.1 | Average in ROI | FD>0.05mm, or rotation >1.15° | Run if >20% of frames are removed |
| **Han 2019** | | SPM12, GIFT | 9 motion ICAs, motion & 1^st^ derivatives, detrend | NA | 200µm | NA | Run with max FD>0.075mm |
| **Chen 2020*** | | SPM12, MATLAB | Motion & 1^st^ derivatives & 10 PCs | 0.01 < *f* | 400µm |  | NA |
| **Xu 2021** | |  | NA | NA |  |  |  |
| **Jonkers 2014** | | SPM8, GIFT, MATLAB, SPSS | Motion, detrend, GS | 0.01 < *f* < 0.1 | 2x voxel size | NA | NA |
| **Gutierrez-Barragan 2022*** | | AFNI, FSL, ANTs | Motion & 1^st^ derivatives & squares, CSF |  | 500µm | FD>0.075mm |  |
| **Desjardins 2019** | | SPM12, MATLAB | NA | NA | 600µm | NA | Trials with motion |
| **Harris 2015** | | SPM8 | Motion | ~0.002 < *f* |  |  | Run mean >0.15mm, or >0.3° |
| **Sakurai 2020** | | SPM12, ANTs |  | NA | 800µm |  | NA |
| **Zeng 2022** | | AFNI, MATLAB | Detrend | NA | 200µm |  |  |
| **Dinh 2021*** | | AFNI, FMRIB, ANTs, MATLAB, FSL | Motion & 1^st^ derivatives, detrend | NA | 300µm |  | Trail FD>0.15mm |
| **Cover 2021** | | AFNI | Motion, ON/OFF cue, detrend | NA | NA | FD>0.075mm | Run if >20% of frames are removed |
| **Fadel 2022** | | FSL, CONN | Motion | *f* < 0.1 | NA | >1SD from group mean, or >0.125mm | Run if >10% of frames are removed |

* Results from different motion regression approaches compared

‘Motion’ is 6-parameters (3 translations, 3 rotations). ‘Motion & 1^st^ derivative’ is the first temporal derivative of the 6-parameters (i.e., is a 12-parameter model).

Frequency (*f*) filter bandwidths (in Hz) applied to remove high and/or low frequency oscillations that are not of interest.

Smoothing parameter applied to improve SNR by averaging data spatially (parameters are a Gaussian kernel FWHM, full width half maximum, unless specified otherwise).

Exclusion criteria of individual frames (i.e., censoring), or of runs/scans/trails/sessions.

FD (framewise displacement), DVARS (derivative root mean square variance over voxels), IQR (interquartile range), WM (white matter), CSF (cerebral spinal fluid), GS (global signal), MAD (median absolute deviation), PCs (principal components), SPM (statistical parametric mapping), FSL (Oxford Centre for Functional Magnetic Resonance Imaging of the Brain Software Library), AFNI (Analysis of Functional NeuroImages), ANTs (Advances Normalization Tools), GIFT (Group ICA, independent component analysis, of fMRI toolbox), Icasso (Himberg et al. 2004), SPSS (Statistical Package for Social Sciences), ICA-AROMA (Independent Component Analysis – Automatic Removal of Motion Artifacts), CONN (Whitfield-Gabrieli et al. 2012).

**Table S9.** **Exclusions for motion with 6-parameter motion outcome measures.**

| **Study** | **Exclusions** | **Group** | | **Outcome (mean/median ± SD)** | | | | | |
| --- | --- | --- | --- | --- | --- | --- | --- | --- | --- |
|  | **(N)** |  |  | **X µm** | **Y µm** | **Z µm** | **Pitch** | **Yaw** | **Roll** |
| **Desai 2011** | 0/5 mice | Day 1 | | 570 ± 10 | 75 ± 40 | 140 ± 75 | 370 ± 100µm | 220 ± 50µm | 125 ± 75µm |
|  |  | Day 4 | | 160 ± 20 | 20 ± 20 | 40 ± 20 | 25 ± 20µm | 75 ± 20µm | 75 ± 20µm |
| **Ferris 2014** | 0/29 mice | Awake | | 8 ± 9 | 2 ± 8 | 3 ± 3 | 0.00006 ± 0.0003° | 0.0007  ± 0.0007° | 0.00004 ± 0.0001° |
| **Yoshida 2016** | 1/9 mice | Awake | | 15 ± 4 | 11 ± 7 | 6 ± 2 | 0.07 ± 0.09° | 0.07 ± 0.08° | 0.07 ± 0.07° |
|  | 0/8 mice | Anesthesia | | 5 ± 3 | 4 ± 2 | 7 ± 3 | 0.04 ± 0.05° | 0.03 ± 0.03° | 0.04 ± 0.04° |
| **Chen 2020**^♦^ | 0/14 (Som.),  0/13 (Aud.),  0/13 (Olf.) mice | TR 1.5  sec | Som. | 5 ± 2.5 | 29 ± 12 | 5 ± 2.5 | 0.25 ± 0.15° | 0.10 ± 0.05° | 0.05 ± 0.02° |
|  |  |  | Aud. | 5 ± 2.5 | 22 ± 12 | 8 ± 4 | 0.25 ± 0.15° | 0.10 ± 0.05° | 0.05 ± 0.02° |
|  |  |  | Olf. | 2.5 ± 2.5 | 8 ± 4 | 3 ± 2.5 | 0.22 ± 0.15° | 0.15 ± 0.05° | 0.05 ± 0.05° |
|  |  | TR 0.35sec | Som. | 8 ± 4 | 22 ± 11 | 9 ± 4 | 0.20 ± 0.10° | 0.15 ± 0.06° | 0.15 ± 0.05° |
|  |  |  | Aud. | 9 ± 4 | 22 ± 11 | 9 ± 4 | 0.22 ± 0.12° | 0.16 ± 0.08° | 0.15 ± 0.06° |
|  |  |  | Olf. | 8 ± 3 | 18 ± 10 | 8 ± 4 | 0.30 ± 0.15° | 0.17 ± 0.05° | 0.19 ± 0.05° |
| **Tsurugizawa 2020**^♦^ | 0/50 mice | Day 3 | | 21 ± 5 | 60 ± 10 | 60 ± 12 | 0.01 ± 0.008° | 0.03 ± 0.006° | 0.06 ± 0.007° |
|  |  | Day 5 | | 20 ± 5 | 50 ± 10 | 58 ± 8 | 0.01 ± 0.008° | 0.02 ± 0.006° | 0.05 ± 0.007° |
| **Sadaka 2021** | 39/60 mice* | Awake | | 56 ± 52 | 54 ± 49 | 8 ± 16 | NA | | |
| **Zeng 2022** | 0/6 mice | 1^st^ week | | 40 ± 30 | 3 ± 2 | 40 ± 30 | 0.27 ± 0.22° | 0.08 ± 0.05° | 0.05 ± 0.05° |
|  |  | 5^th^ week | | 10 ± 10 | 2 ± 2 | 10 ± 10 | 0.11 ± 0.09° | 0.05 ± 0.04° | 0.03 ± 0.02° |
|  |  | 8^th^ week | | 10 ± 9 | 2 ± 1 | 3 ± 2 | 0.14 ± 0.08° | 0.02 ± 0.01° | 0.03 ± 0.02° |
| **Harris 2015** | 5/21 mice | Awake | | 60 ± 27 (translation) | | | ~0.06° (rotation) | | |
| **Madularu 2017^a^**^♦^ | 8/66 scans 0/33 mice | Sess. 1 | | 0 ± 20 | 0 ± 40 | 0 ± 60 | NA | | |
|  |  | Sess. 2 | |  |  |  |  |  |  |
| **Sakurai 2020**^♦^ | 0/3 mice | AD model | | 0-50 | 0-125 | 0-50 | 0-0.70° | 0-0.70° | 0-0.25° |
|  | 0/3 mice | WT mice | | 0-25 | 0-200 | 0-50 | 0-0.80° | 0-0.40° | 0-0.40° |

* N=34/60 mice were not successfully habituated.

Of these, N=5/34 had motion or technical complications.

Thus, N=18 mice were left and included in the analyses (estimates of motion).

^♦^ Values inferred from figure or plot.

Values appearing above the dashed grey line are plotted (**Figure 4**).

Motion where translation, given in µm, in X corresponds to superior-inferior (SI), Y corresponds to left-right (LR), and Z corresponds to anterior-posterior (AP).

Rotations are listed in degrees or µm as indicated.

SD (standard deviation), FD (framewise displacement), Sess. (session), AD (Alzheimer’s disease), WT (wild type), TR (repetition time), sec (second), Som. (somatosensory), Aud. (auditory), Olf. (olfactory).

**Table S10.** **Exclusions for motion with framewise displacement outcome measures.**

| **Study** | **Exclusions** | **Group** | | **Outcome (mean ± SD)** |
| --- | --- | --- | --- | --- |
|  | **(N)** |  |  | **FD µm** |
| **Bergmann 2016^‡^** | 32/132 sess.  0/12 mice | NA | | NA |
| **Han 2019** | 16/76 scans  0/8 mice | All data | | 7 ± 1 |
|  |  | With exclusions | | 1.1 ± 0.5 |
| **Desjardins 2019**^☨^ | 15-20% trials | Sensory stimuli | | NA |
|  | 5-10% trials | Optogenetic stimuli | |  |
| **Shofty 2019** | 20/128 sess.  0/22 mice | Group 1 | | 48 ± 7 |
|  |  | Group 2 | | 51 ± 6 |
| **Chen 2020*** | 0/14 (Som.),  0/13 (Aud.),  0/13 (Olf.) mice | TR 1.5 sec | Som. | 8 ± 5 |
|  |  |  | Aud. | 9 ± 6 |
|  |  |  | Olf. | 5 ± 2.5 |
|  |  | TR 0.35 sec | Som. | 7 ± 4 |
|  |  |  | Aud. | 9 ± 6 |
|  |  |  | Olf. | 8 ± 5 |
| **Asleh 2020** | 29/208 sess.  0/18 mice | NA | | NA |
| **Bergmann 2020**^♦^ | 6/96 sess.  2/19 mice | Analysis 1 (ID) | |  |
|  | 6/96 sess.  2/19 mice | Analysis 2 (CPM) | |  |
| **Dinh 2021** | 7/43 trials  0/3 mice | Actual system | | 17 ± 2.5 |
|  | 22/52 trials  1/4 mice | Mock system | | 40 ± 13 |
|  | 0/6 mice | Anesthetized | | 4.0 ± 0.4 |
| **Takata 2021** | 3% frames | NA | | NA |
| **Tsurugizawa 2021^b^** | 0/9 mice | Awake | | 25 ± 10* |
|  | 0/9 mice | Iso. | | 10 ± 2* |
|  | 0/9 mice | Iso. & Med. | | 7 ± 2* |
| **Gutierrez-Barragan 2022** | 11% frames*  0/10 mice | Awake | | 40 ± 5* |
|  | 1% frames*  0/33 mice | Anesthetized | | 30 ± 5* |
| **Fadel 2022** | 0/11 mice | Awake | | 12 ± 40 |
|  | 0/5 mice | Anesthetized | | 3 ± 3 |

* Values inferred from figure or plot

^♦^ Assumptions made to infer numbers from text that was not explicit.

^☨^ Motion monitored using videography

^‡^ Reason for exclusion was listed as ‘motion or other’.

SD (standard deviation), FD (framewise displacement), Sess. (session), ID (identification), CPM (connectivity predictive modeling).

**Table S11.** **Exclusion criteria other than motion.**

| **Study** | **Exclusions** | **Group** | **Reason** |
| --- | --- | --- | --- |
|  | **(N)** |  |  |
| **Bergmann 2016** | 32/132 sess. | Awake | Imaging artifacts or motion |
| **Bergmann 2020** | 1/19 mice | All analyses  (**Table 7**) | Susceptibility artifacts |
|  | 2/19 sessions |  | Ghosting |
| **Cover 2021** | 5/15 mice | Awake | Lack of viral expression |
| **Xu 2021** | 5/22 mice | Awake | Death |
|  | 1/22 mice |  | Outcome measures out of range |
|  | 2/22 mice |  | Physiological measure out of range |
| **Zeng 2022*** | 2/9 mice | Awake | Head-post crack |

* Data from only N=6 or N=3 mice shown in figures

**Table S12.** **Full list of articles on rats included in this review.**

| **#** | **Study** | **Full Reference** |
| --- | --- | --- |
| **1** | **Lahti 1998** | Lahti KM, Ferris CF, Li F, Sotak CH, King JA. Imaging brain activity in conscious animals using functional MRI. J Neurosci Methods. 1998 Jul 1;82(1):75-83. doi: 10.1016/s0165-0270(98)00037-5. PMID: 10223517. |
| **2** | **Lahti 1999** | Lahti KM, Ferris CF, Li F, Sotak CH, King JA. Comparison of evoked cortical activity in conscious and propofol-anesthetized rats using functional MRI. Magn Reson Med. 1999 Feb;41(2):412-6. doi: 10.1002/(sici)1522-2594(199902)41:2<412::aid-mrm28>3.0.co;2-3. PMID: 10080292. |
| **3** | **Tenney 2003** | Tenney JR, Duong TQ, King JA, Ludwig R, Ferris CF. Corticothalamic modulation during absence seizures in rats: a functional MRI assessment. Epilepsia. 2003 Sep;44(9):1133-40. doi: 10.1046/j.1528-1157.2003.61002.x. PMID: 12919383; PMCID: PMC2962948. |
| **4** | **Sicard 2003** | Sicard K, Shen Q, Brevard ME, Sullivan R, Ferris CF, King JA, Duong TQ. Regional cerebral blood flow and BOLD responses in conscious and anesthetized rats under basal and hypercapnic conditions: implications for functional MRI studies. J Cereb Blood Flow Metab. 2003 Apr;23(4):472-81. doi: 10.1097/01.WCB.0000054755.93668.20. PMID: 12679724; PMCID: PMC2989608. |
| **5** | **Brevard 2003** | Brevard ME, Duong TQ, King JA, Ferris CF. Changes in MRI signal intensity during hypercapnic challenge under conscious and anesthetized conditions. Magn Reson Imaging. 2003 Nov;21(9):995-1001. doi: 10.1016/s0730-725x(03)00204-2. PMID: 14684202; PMCID: PMC2962949. |
| **6** | **Tenney 2004** | Tenney JR, Duong TQ, King JA, Ferris CF. FMRI of brain activation in a genetic rat model of absence seizures. Epilepsia. 2004 Jun;45(6):576-82. doi: 10.1111/j.0013-9580.2004.39303.x. PMID: 15144421; PMCID: PMC2949946. |
| **7** | **Febo 2004** | Febo M, Segarra AC, Tenney JR, Brevard ME, Duong TQ, Ferris CF. Imaging cocaine-induced changes in the mesocorticolimbic dopaminergic system of conscious rats. J Neurosci Methods. 2004 Oct 30;139(2):167-76. doi: 10.1016/j.jneumeth.2004.04.028. PMID: 15488229; PMCID: PMC2949948. |
| **8** | **Ferris 2005** | Ferris CF, Kulkarni P, Sullivan JM Jr, Harder JA, Messenger TL, Febo M. Pup suckling is more rewarding than cocaine: evidence from functional magnetic resonance imaging and three-dimensional computational analysis. J Neurosci. 2005 Jan 5;25(1):149-56. doi: 10.1523/JNEUROSCI.3156-04.2005. PMID: 15634776; PMCID: PMC6725197. |
| **9** | **Febo 2005^a^** | Febo M, Ferris CF, Segarra AC. Estrogen influences cocaine-induced blood oxygen level-dependent signal changes in female rats. J Neurosci. 2005 Feb 2;25(5):1132-6. doi: 10.1523/JNEUROSCI.3801-04.2005. PMID: 15689549; PMCID: PMC6725975. |
| **10** | **Febo 2005^b^** | Febo M, Segarra AC, Nair G, Schmidt K, Duong TQ, Ferris CF. The neural consequences of repeated cocaine exposure revealed by functional MRI in awake rats. Neuropsychopharmacology. 2005 May;30(5):936-43. doi: 10.1038/sj.npp.1300653. PMID: 15637636; PMCID: PMC2962946. |
| **11** | **King 2005** | King JA, Garelick TS, Brevard ME, Chen W, Messenger TL, Duong TQ, Ferris CF. Procedure for minimizing stress for fMRI studies in conscious rats. J Neurosci Methods. 2005 Oct 30;148(2):154-60. doi: 10.1016/j.jneumeth.2005.04.011. Epub 2005 Jun 16. PMID: 15964078; PMCID: PMC2962951. |
| **12** | **Duong 2007** | Duong TQ. Cerebral blood flow and BOLD fMRI responses to hypoxia in awake and anesthetized rats. Brain Res. 2007 Mar 2;1135(1):186-94. doi: 10.1016/j.brainres.2006.11.097. Epub 2007 Jan 2. PMID: 17198686; PMCID: PMC2949962. |
| **13** | **Ferris 2008** | Ferris CF, Stolberg T, Kulkarni P, Murugavel M, Blanchard R, Blanchard DC, Febo M, Brevard M, Simon NG. Imaging the neural circuitry and chemical control of aggressive motivation. BMC Neurosci. 2008 Nov 13;9:111. doi: 10.1186/1471-2202-9-111. PMID: 19014547; PMCID: PMC2601047. |
| **14** | **Febo 2009** | Febo M, Akbarian S, Schroeder FA, Ferris CF. Cocaine-induced metabolic activation in cortico-limbic circuitry is increased after exposure to the histone deacetylase inhibitor, sodium butyrate. Neurosci Lett. 2009 Nov 20;465(3):267-71. doi: 10.1016/j.neulet.2009.07.065. Epub 2009 Jul 26. PMID: 19638299; PMCID: PMC2760625. |
| **15** | **Ferris 2010** | Ferris CF, Stolberg T. Imaging the immediate non-genomic effects of stress hormone on brain activity. Psychoneuroendocrinology. 2010 Jan;35(1):5-14. doi: 10.1016/j.psyneuen.2009.09.003. PMID: 19783105. |
| **16** | **Caffrey 2010** | Caffrey MK, Nephew BC, Febo M. Central vasopressin V1a receptors modulate neural processing in mothers facing intruder threat to pups. Neuropharmacology. 2010 Jan;58(1):107-16. doi: 10.1016/j.neuropharm.2009.06.023. Epub 2009 Jun 30. Erratum in: Neuropharmacology. 2010 Sep;59(3):218. PMID: 19573540; PMCID: PMC2784100. |
| **17** | **Kulkarni 2012** | Kulkarni P, Stolberg T, Sullivanjr JM, Ferris CF. Imaging evolutionarily conserved neural networks: preferential activation of the olfactory system by food-related odor. Behav Brain Res. 2012 Apr 21;230(1):201-7. doi: 10.1016/j.bbr.2012.02.002. Epub 2012 Feb 11. PMID: 22343130. |
| **18** | **Johnson 2013** | Johnson TR, Smerkers B, Moulder JK, Stellar JR, Febo M. Neural processing of a cocaine-associated odor cue revealed by functional MRI in awake rats. Neurosci Lett. 2013 Feb 8;534:160-5. doi: 10.1016/j.neulet.2012.11.054. Epub 2012 Dec 20. PMID: 23262077; PMCID: PMC3809094. |
| **19** | **Reed 2013** | Reed MD, Price KE, Archbold J, Moffa A, Febo M. Predator odor-evoked BOLD activation in the awake rat: modulation by oxytocin and V₁a vasopressin receptor antagonists. Brain Res. 2013 Feb 4;1494:70-83. doi: 10.1016/j.brainres.2012.11.045. Epub 2012 Dec 3. PMID: 23219972; PMCID: PMC3809104. |
| **20** | **Shah 2014** | Shah L, Kulkarni P, Ferris C, Amiji MM. Analgesic efficacy and safety of DALDA peptide analog delivery to the brain using oil-in-water nanoemulsion formulation. Pharm Res. 2014 Oct;31(10):2724-34. doi: 10.1007/s11095-014-1370-y. Epub 2014 May 3. PMID: 24792826; PMCID: PMC4198448. |
| **21** | **Yee 2015** | Yee JR, Kenkel W, Caccaviello JC, Gamber K, Simmons P, Nedelman M, Kulkarni P, Ferris CF. Identifying the integrated neural networks involved in capsaicin-induced pain using fMRI in awake TRPV1 knockout and wild-type rats. Front Syst Neurosci. 2015 Feb 19;9:15. doi: 10.3389/fnsys.2015.00015. PMID: 25745388; PMCID: PMC4333803. |
| **22** | **Madularu 2015** | Madularu D, Yee JR, Kenkel WM, Moore KA, Kulkarni P, Shams WM, Ferris CF, Brake WG. Integration of neural networks activated by amphetamine in females with different estrogen levels: a functional imaging study in awake rats. Psychoneuroendocrinology. 2015 Jun;56:200-12. doi: 10.1016/j.psyneuen.2015.02.022. Epub 2015 Mar 20. PMID: 25827963. |
| **23** | **Ferris 2015** | Ferris CF, Yee JR, Kenkel WM, Dumais KM, Moore K, Veenema AH, Kulkarni P, Perkybile AM, Carter CS. Distinct BOLD Activation Profiles Following Central and Peripheral Oxytocin Administration in Awake Rats. Front Behav Neurosci. 2015 Sep 17;9:245. doi: 10.3389/fnbeh.2015.00245. PMID: 26441574; PMCID: PMC4585275. |
| **24** | **Kenkel 2016** | Kenkel WM, Yee JR, Moore K, Madularu D, Kulkarni P, Gamber K, Nedelman M, Ferris CF. Functional magnetic resonance imaging in awake transgenic fragile X rats: evidence of dysregulation in reward processing in the mesolimbic/habenular neural circuit. Transl Psychiatry. 2016 Mar 22;6(3):e763. doi: 10.1038/tp.2016.15. PMID: 27003189; PMCID: PMC4872441. |
| **25** | **Madularu 2016** | Madularu D, Kulkarni P, Yee JR, Kenkel WM, Shams WM, Ferris CF, Brake WG. High estrogen and chronic haloperidol lead to greater amphetamine-induced BOLD activation in awake, amphetamine-sensitized female rats. Horm Behav. 2016 Jun;82:56-63. doi: 10.1016/j.yhbeh.2016.04.007. Epub 2016 May 15. PMID: 27154458. |
| **26** | **Ferris 2017** | Ferris CF, Kulkarni P, Yee JR, Nedelman M, de Jong IEM. The Serotonin Receptor 6 Antagonist Idalopirdine and Acetylcholinesterase Inhibitor Donepezil Have Synergistic Effects on Brain Activity-A Functional MRI Study in the Awake Rat. Front Pharmacol. 2017 Jun 12;8:279. doi: 10.3389/fphar.2017.00279. PMID: 28659792; PMCID: PMC5467007. |
| **27** | **Dumais 2017** | Dumais KM, Kulkarni PP, Ferris CF, Veenema AH. Sex differences in neural activation following different routes of oxytocin administration in awake adult rats. Psychoneuroendocrinology. 2017 Jul;81:52-62. doi: 10.1016/j.psyneuen.2017.04.003. Epub 2017 Apr 7. PMID: 28412582; PMCID: PMC5497485. |
| **28** | **Madularu 2017^b^** | Madularu D, Yee JR, Kulkarni P, Ferris CF. System-specific activity in response to Δ^9^ -tetrahydrocannabinol: a functional magnetic resonance imaging study in awake male rats. Eur J Neurosci. 2017 Dec;46(12):2893-2900. doi: 10.1111/ejn.13754. Epub 2017 Nov 6. PMID: 29057576. |
| **29** | **Iriah 2019** | Iriah SC, Trivedi M, Kenkel W, Grant SE, Moore K, Yee JR, Madularu D, Kulkarni P, Ferris CF. Oxycodone Exposure: A Magnetic Resonance Imaging Study in Response to Acute and Chronic Oxycodone Treatment in Rats. Neuroscience. 2019 Feb 1;398:88-101. doi: 10.1016/j.neuroscience.2018.11.042. Epub 2018 Dec 12. PMID: 30550747. |
| **30** | **Zhang 2010** | Zhang N, Rane P, Huang W, Liang Z, Kennedy D, Frazier JA, King J. Mapping resting-state brain networks in conscious animals. J Neurosci Methods. 2010 Jun 15;189(2):186-96. doi: 10.1016/j.jneumeth.2010.04.001. Epub 2010 Apr 9. PMID: 20382183; PMCID: PMC2896018. |
| **31** | **Huang 2011** | Huang W, Heffernan ME, Li Z, Zhang N, Overstreet DH, King JA. Fear induced neuronal alterations in a genetic model of depression: an fMRI study on awake animals. Neurosci Lett. 2011 Feb 4;489(2):74-8. doi: 10.1016/j.neulet.2010.11.069. Epub 2010 Dec 4. PMID: 21134416; PMCID: PMC3022083. |
| **32** | **Liang 2011** | Liang Z, King J, Zhang N. Uncovering intrinsic connectional architecture of functional networks in awake rat brain. J Neurosci. 2011 Mar 9;31(10):3776-83. doi: 10.1523/JNEUROSCI.4557-10.2011. PMID: 21389232; PMCID: PMC3073070. |
| **33** | **King 2011** | King J, Huang W, Chen W, Heffernan M, Shields J, Rane P, Bircher R, DiFranza JR. A comparison of brain and behavioral effects of varenicline and nicotine in rats. Behav Brain Res. 2011 Sep 30;223(1):42-7. doi: 10.1016/j.bbr.2011.04.012. Epub 2011 Apr 17. PMID: 21527291. |
| **34** | **Liang 2012** | Liang Z, King J, Zhang N. Anticorrelated resting-state functional connectivity in awake rat brain. Neuroimage. 2012 Jan 16;59(2):1190-9. doi: 10.1016/j.neuroimage.2011.08.009. Epub 2011 Aug 12. PMID: 21864689; PMCID: PMC3230741. |
| **35** | **Liang 2014** | Liang Z, King J, Zhang N. Neuroplasticity to a single-episode traumatic stress revealed by resting-state fMRI in awake rats. Neuroimage. 2014 Dec;103:485-491. doi: 10.1016/j.neuroimage.2014.08.050. Epub 2014 Sep 3. PMID: 25193500; PMCID: PMC4253640. |
| **36** | **Crenshaw 2015** | Crenshaw DG, Asin K, Gottschalk WK, Liang Z, Zhang N, Roses AD. Effects of low doses of pioglitazone on resting-state functional connectivity in conscious rat brain. PLoS One. 2015 Feb 11;10(2):e0117973. doi: 10.1371/journal.pone.0117973. PMID: 25671601; PMCID: PMC4324644. |
| **37** | **Liang 2015** | Liang Z, Watson GD, Alloway KD, Lee G, Neuberger T, Zhang N. Mapping the functional network of medial prefrontal cortex by combining optogenetics and fMRI in awake rats. Neuroimage. 2015 Aug 15;117:114-23. doi: 10.1016/j.neuroimage.2015.05.036. Epub 2015 May 20. PMID: 26002727; PMCID: PMC4512884. |
| **38** | **Ma 2017** | Ma Y, Hamilton C, Zhang N. Dynamic Connectivity Patterns in Conscious and Unconscious Brain. Brain Connect. 2017 Feb;7(1):1-12. doi: 10.1089/brain.2016.0464. Epub 2016 Dec 15. PMID: 27846731; PMCID: PMC5312592. |
| **39** | **Hamilton 2017** | Hamilton C, Ma Y, Zhang N. Global reduction of information exchange during anesthetic-induced unconsciousness. Brain Struct Funct. 2017 Sep;222(7):3205-3216. doi: 10.1007/s00429-017-1396-0. Epub 2017 Mar 13. PMID: 28289883; PMCID: PMC5597454. |
| **40** | **Pérez 2018** | Pérez PD, Ma Z, Hamilton C, Sánchez C, Mørk A, Pehrson AL, Bundgaard C, Zhang N. Acute effects of vortioxetine and duloxetine on resting-state functional connectivity in the awake rat. Neuropharmacology. 2018 Jan;128:379-387. doi: 10.1016/j.neuropharm.2017.10.038. Epub 2017 Nov 2. PMID: 29104073. |
| **41** | **Ma 2018^a^** | Ma Z, Zhang N. Temporal transitions of spontaneous brain activity. Elife. 2018 Mar 8;7:e33562. doi: 10.7554/eLife.33562. PMID: 29517975; PMCID: PMC5843406. |
| **42** | **Ma 2018^b^** | Ma Z, Perez P, Ma Z, Liu Y, Hamilton C, Liang Z, Zhang N. Functional atlas of the awake rat brain: A neuroimaging study of rat brain specialization and integration. Neuroimage. 2018 Apr 15;170:95-112. doi: 10.1016/j.neuroimage.2016.07.007. Epub 2016 Jul 5. PMID: 27393420; PMCID: PMC5217744. |
| **43** | **Ma 2018^c^** | Ma Z, Ma Y, Zhang N. Development of brain-wide connectivity architecture in awake rats. Neuroimage. 2018 Aug 1;176:380-389. doi: 10.1016/j.neuroimage.2018.05.009. Epub 2018 May 5. PMID: 29738909; PMCID: PMC6345182. |
| **44** | **Dopfel 2019** | Dopfel D, Perez PD, Verbitsky A, Bravo-Rivera H, Ma Y, Quirk GJ, Zhang N. Individual variability in behavior and functional networks predicts vulnerability using an animal model of PTSD. Nat Commun. 2019 May 30;10(1):2372. doi: 10.1038/s41467-019-09926-z. PMID: 31147546; PMCID: PMC6543038. |
| **45** | **Liu 2019** | Liu Y, Zhang N. Propagations of spontaneous brain activity in awake rats. Neuroimage. 2019 Nov 15;202:116176. doi: 10.1016/j.neuroimage.2019.116176. Epub 2019 Sep 9. PMID: 31513942; PMCID: PMC6919312. |
| **46** | **Liu 2020** | Liu Y, Perez PD, Ma Z, Ma Z, Dopfel D, Cramer S, Tu W, Zhang N. An open database of resting-state fMRI in awake rats. Neuroimage. 2020 Oct 15;220:117094. doi: 10.1016/j.neuroimage.2020.117094. Epub 2020 Jun 28. PMID: 32610063; PMCID: PMC7605641. |
| **47** | **Ma 2020** | Ma Y, Ma Z, Liang Z, Neuberger T, Zhang N. Global brain signal in awake rats. Brain Struct Funct. 2020 Jan;225(1):227-240. doi: 10.1007/s00429-019-01996-5. Epub 2019 Dec 4. PMID: 31802256; PMCID: PMC7391791. |
| **48** | **Tu 2021^a^** | Tu W, Ma Z, Ma Y, Dopfel D, Zhang N. Suppressing Anterior Cingulate Cortex Modulates Default Mode Network and Behavior in Awake Rats. Cereb Cortex. 2021 Jan 1;31(1):312-323. doi: 10.1093/cercor/bhaa227. PMID: 32820327; PMCID: PMC7727348. |
| **49** | **Tu 2021^b^** | Tu W, Ma Z, Zhang N. Brain network reorganization after targeted attack at a hub region. Neuroimage. 2021 Aug 15;237:118219. doi: 10.1016/j.neuroimage.2021.118219. Epub 2021 May 27. PMID: 34052466; PMCID: PMC8289586. |
| **50** | **Ma 2022** | Ma Z, Zhang Q, Tu W, Zhang N. Gaining insight into the neural basis of resting-state fMRI signal. Neuroimage. 2022 Apr 15;250:118960. doi: 10.1016/j.neuroimage.2022.118960. Epub 2022 Feb 1. PMID: 35121182; PMCID: PMC8935501. |
| **51** | **Zhang 2022** | Zhang Q, Cramer SR, Ma Z, Turner KL, Gheres KW, Liu Y, Drew PJ, Zhang N. Brain-wide ongoing activity is responsible for significant cross-trial BOLD variability. Cereb Cortex. 2022 Nov 21;32(23):5311-5329. doi: 10.1093/cercor/bhac016. PMID: 35179203; PMCID: PMC9712744. |
| **52** | **Han 2022** | Han X, Cramer SR, Zhang N. Deriving causal relationships in resting-state functional connectivity using SSFO-based optogenetic fMRI. J Neural Eng. 2022 Nov 8;19(6):10.1088/1741-2552/ac9d66. doi: 10.1088/1741-2552/ac9d66. PMID: 36301683; PMCID: PMC9681600. |
| **53** | **Tabuchi 2002** | Tabuchi E, Yokawa T, Mallick H, Inubushi T, Kondoh T, Ono T, Torii K. Spatio-temporal dynamics of brain activated regions during drinking behavior in rats. Brain Res. 2002 Oct 4;951(2):270-9. doi: 10.1016/s0006-8993(02)03173-6. PMID: 12270506. |
| **54** | **Khubchandani 2003** | Khubchandani M, Mallick HN, Jagannathan NR, Mohan Kumar V. Stereotaxic assembly and procedures for simultaneous electrophysiological and MRI study of conscious rat. Magn Reson Med. 2003 May;49(5):962-7. doi: 10.1002/mrm.10441. PMID: 12704780. |
| **55** | **Khubchandani 2005** | Khubchandani M, Jagannathan NR, Mallick HN, Mohan Kumar V. Functional MRI shows activation of the medial preoptic area during sleep. Neuroimage. 2005 May 15;26(1):29-35. doi: 10.1016/j.neuroimage.2005.01.002. PMID: 15862202. |
| **56** | **Tsurugizawa 2010^a^** | Tsurugizawa T, Uematsu A, Uneyama H, Torii K. Effects of isoflurane and alpha-chloralose anesthesia on BOLD fMRI responses to ingested L-glutamate in rats. Neuroscience. 2010 Jan 13;165(1):244-51. doi: 10.1016/j.neuroscience.2009.10.006. Epub 2009 Oct 9. PMID: 19819307. |
| **57** | **Tsurugizawa 2010^b^** | Tsurugizawa T, Uematsu A, Uneyama H, Torii K. The role of the GABAergic and dopaminergic systems in the brain response to an intragastric load of alcohol in conscious rats. Neuroscience. 2010 Dec 1;171(2):451-60. doi: 10.1016/j.neuroscience.2010.09.016. Epub 2010 Sep 16. PMID: 20849934. |
| **58** | **Tsurugizawa 2012** | Tsurugizawa T, Uematsu A, Uneyama H, Torii K. Functional brain mapping of conscious rats during reward anticipation. J Neurosci Methods. 2012;206(2):132-7. doi: 10.1016/j.jneumeth.2012.02.014. Epub 2012 Feb 22. PMID: 22387258. |
| **59** | **Uematsu 2015** | Uematsu A, Kitamura A, Iwatsuki K, Uneyama H, Tsurugizawa T. Correlation Between Activation of the Prelimbic Cortex, Basolateral Amygdala, and Agranular Insular Cortex During Taste Memory Formation. Cereb Cortex. 2015 Sep;25(9):2719-28. doi: 10.1093/cercor/bhu069. Epub 2014 Apr 15. PMID: 24735672. |
| **60** | **Kitamura 2019** | Kitamura A, Hojo Y, Ikeda M, Karakawa S, Kuwahara T, Kim J, Soma M, Kawato S, Tsurugizawa T. Ingested d-Aspartate Facilitates the Functional Connectivity and Modifies Dendritic Spine Morphology in Rat Hippocampus. Cereb Cortex. 2019 Jun 1;29(6):2499-2508. doi: 10.1093/cercor/bhy120. PMID: 29850790. |
| **61** | **Skoubis 2006** | Skoubis PD, Hradil V, Chin CL, Luo Y, Fox GB, McGaraughty S. Mapping brain activity following administration of a nicotinic acetylcholine receptor agonist, ABT-594, using functional magnetic resonance imaging in awake rats. Neuroscience. 2006;137(2):583-91. doi: 10.1016/j.neuroscience.2005.08.072. Epub 2005 Nov 14. PMID: 16289887. |
| **62** | **Chin 2008^a^** | Chin CL, Pauly JR, Surber BW, Skoubis PD, McGaraughty S, Hradil VP, Luo Y, Cox BF, Fox GB. Pharmacological MRI in awake rats predicts selective binding of alpha4beta2 nicotinic receptors. Synapse. 2008 Mar;62(3):159-68. doi: 10.1002/syn.20474. PMID: 18081183. |
| **63** | **Chin 2008^b^** | Chin CL, Tovcimak AE, Hradil VP, Seifert TR, Hollingsworth PR, Chandran P, Zhu CZ, Gauvin D, Pai M, Wetter J, Hsieh GC, Honore P, Frost JM, Dart MJ, Meyer MD, Yao BB, Cox BF, Fox GB. Differential effects of cannabinoid receptor agonists on regional brain activity using pharmacological MRI. Br J Pharmacol. 2008 Jan;153(2):367-79. doi: 10.1038/sj.bjp.0707506. Epub 2007 Oct 29. PMID: 17965748; PMCID: PMC2219521. |
| **64** | **Chin 2011** | Chin CL, Upadhyay J, Marek GJ, Baker SJ, Zhang M, Mezler M, Fox GB, Day M. Awake rat pharmacological magnetic resonance imaging as a translational pharmacodynamic biomarker: metabotropic glutamate 2/3 agonist modulation of ketamine-induced blood oxygenation level dependence signals. J Pharmacol Exp Ther. 2011 Mar;336(3):709-15. doi: 10.1124/jpet.110.173880. Epub 2010 Dec 20. Erratum in: J Pharmacol Exp Ther. 2011 May;337(2):567. PMID: 21172908. |
| **65** | **Upadhyay 2011** | Upadhyay J, Baker SJ, Chandran P, Miller L, Lee Y, Marek GJ, Sakoglu U, Chin CL, Luo F, Fox GB, Day M. Default-mode-like network activation in awake rodents. PLoS One. 2011;6(11):e27839. doi: 10.1371/journal.pone.0027839. Epub 2011 Nov 18. PMID: 22125628; PMCID: PMC3220684. |
| **66** | **Baker 2012** | Baker S, Chin CL, Basso AM, Fox GB, Marek GJ, Day M. Xanomeline modulation of the blood oxygenation level-dependent signal in awake rats: development of pharmacological magnetic resonance imaging as a translatable pharmacodynamic biomarker for central activity and dose selection. J Pharmacol Exp Ther. 2012 Apr;341(1):263-73. doi: 10.1124/jpet.111.188797. Epub 2012 Jan 20. PMID: 22267203. |
| **67*** | **Brydges 2013** | Brydges NM, Whalley HC, Jansen MA, Merrifield GD, Wood ER, Lawrie SM, Wynne SM, Day M, Fleetwood-Walker S, Steele D, Marshall I, Hall J, Holmes MC. Imaging conditioned fear circuitry using awake rodent fMRI. PLoS One. 2013;8(1):e54197. doi: 10.1371/journal.pone.0054197. Epub 2013 Jan 22. PMID: 23349824; PMCID: PMC3551953. |
| **68*** | **Harris 2016** | Harris AP, Lennen RJ, Brydges NM, Jansen MA, Pernet CR, Whalley HC, Marshall I, Baker S, Basso AM, Day M, Holmes MC, Hall J. The role of brain-derived neurotrophic factor in learned fear processing: an awake rat fMRI study. Genes Brain Behav. 2016 Feb;15(2):221-30. doi: 10.1111/gbb.12277. Epub 2016 Jan 5. PMID: 26586578; PMCID: PMC4819698. |
| **69** | **Becerra 2011^a^** | Becerra L, Chang PC, Bishop J, Borsook D. CNS activation maps in awake rats exposed to thermal stimuli to the dorsum of the hindpaw. Neuroimage. 2011 Jan 15;54(2):1355-66. doi: 10.1016/j.neuroimage.2010.08.056. Epub 2010 Sep 9. PMID: 20817102. |
| **70** | **Becerra 2011^b^** | Becerra L, Pendse G, Chang PC, Bishop J, Borsook D. Robust reproducible resting state networks in the awake rodent brain. PLoS One. 2011;6(10):e25701. doi: 10.1371/journal.pone.0025701. Epub 2011 Oct 18. PMID: 22028788; PMCID: PMC3196498. |
| **71** | **Chang 2016** | Chang PC, Procissi D, Bao Q, Centeno MV, Baria A, Apkarian AV. Novel method for functional brain imaging in awake minimally restrained rats. J Neurophysiol. 2016 Jul 1;116(1):61-80. doi: 10.1152/jn.01078.2015. Epub 2016 Apr 6. PMID: 27052584; PMCID: PMC4961750. |
| **72** | **Chang 2017** | Chang PC, Centeno MV, Procissi D, Baria A, Apkarian AV. Brain activity for tactile allodynia: a longitudinal awake rat functional magnetic resonance imaging study tracking emergence of neuropathic pain. Pain. 2017 Mar;158(3):488-497. doi: 10.1097/j.pain.0000000000000788. PMID: 28135213; PMCID: PMC5303183. |
| **73** | **Becerra 2017** | Becerra L, Bishop J, Barmettler G, Kainz V, Burstein R, Borsook D. Brain network alterations in the inflammatory soup animal model of migraine. Brain Res. 2017 Apr 1;1660:36-46. doi: 10.1016/j.brainres.2017.02.001. Epub 2017 Feb 3. PMID: 28167076; PMCID: PMC5731648. |
| **74** | **Bishop 2019** | Bishop J, Becerra L, Barmettler G, Chang PC, Kainz V, Burstein R, Borsook D. Modulation of brain networks by sumatriptan-naproxen in the inflammatory soup migraine model. Pain. 2019 Sep;160(9):2161-2171. doi: 10.1097/j.pain.0000000000001583. PMID: 31033778; PMCID: PMC7193782. |
| **75** | **Wei 2021** | Wei X, Centeno MV, Ren W, Borruto AM, Procissi D, Xu T, Jabakhanji R, Mao Z, Kim H, Li Y, Yang Y, Gutruf P, Rogers JA, Surmeier DJ, Radulovic J, Liu X, Martina M, Apkarian AV. Activation of the dorsal, but not the ventral, hippocampus relieves neuropathic pain in rodents. Pain. 2021 Dec 1;162(12):2865-2880. doi: 10.1097/j.pain.0000000000002279. PMID: 34160168; PMCID: PMC8464622. |
| **76** | **Airaksinen 2012** | Airaksinen AM, Hekmatyar SK, Jerome N, Niskanen JP, Huttunen JK, Pitkänen A, Kauppinen RA, Gröhn OH. Simultaneous BOLD fMRI and local field potential measurements during kainic acid-induced seizures. Epilepsia. 2012 Jul;53(7):1245-53. doi: 10.1111/j.1528-1167.2012.03539.x. Epub 2012 Jun 12. PMID: 22690801. |
| **77** | **Paasonen 2018** | Paasonen J, Stenroos P, Salo RA, Kiviniemi V, Gröhn O. Functional connectivity under six anesthesia protocols and the awake condition in rat brain. Neuroimage. 2018 May 15;172:9-20. doi: 10.1016/j.neuroimage.2018.01.014. Epub 2018 Jan 28. PMID: 29414498. |
| **78** | **Stenroos 2018** | Stenroos P, Paasonen J, Salo RA, Jokivarsi K, Shatillo A, Tanila H, Gröhn O. Awake Rat Brain Functional Magnetic Resonance Imaging Using Standard Radio Frequency Coils and a 3D Printed Restraint Kit. Front Neurosci. 2018 Aug 20;12:548. doi: 10.3389/fnins.2018.00548. PMID: 30177870; PMCID: PMC6109636. |
| **79** | **Paasonen 2020** | Paasonen J, Laakso H, Pirttimäki T, Stenroos P, Salo RA, Zhurakovskaya E, Lehto LJ, Tanila H, Garwood M, Michaeli S, Idiyatullin D, Mangia S, Gröhn O. Multi-band SWIFT enables quiet and artefact-free EEG-fMRI and awake fMRI studies in rat. Neuroimage. 2020 Feb 1;206:116338. doi: 10.1016/j.neuroimage.2019.116338. Epub 2019 Nov 12. PMID: 31730923; PMCID: PMC7008094. |
| **80** | **Paasonen 2022** | Paasonen J, Stenroos P, Laakso H, Pirttimäki T, Paasonen E, Salo RA, Tanila H, Idiyatullin D, Garwood M, Michaeli S, Mangia S, Gröhn O. Whole-brain studies of spontaneous behavior in head-fixed rats enabled by zero echo time MB-SWIFT fMRI. Neuroimage. 2022 Apr 15;250:118924. doi: 10.1016/j.neuroimage.2022.118924. Epub 2022 Jan 19. PMID: 35065267; PMCID: PMC9464759. |
| **81** | **Dvořáková 2022** | Dvořáková L, Stenroos P, Paasonen E, Salo RA, Paasonen J, Gröhn O. Light sedation with short habituation time for large-scale functional magnetic resonance imaging studies in rats. NMR Biomed. 2022 Jun;35(6):e4679. doi: 10.1002/nbm.4679. Epub 2022 Jan 19. PMID: 34961988; PMCID: PMC9285600. |
| **82**^♦^ | **Peeters 2001** | Peeters RR, Tindemans I, De Schutter E, Van der Linden A. Comparing BOLD fMRI signal changes in the awake and anesthetized rat during electrical forepaw stimulation. Magn Reson Imaging. 2001 Jul;19(6):821-6. doi: 10.1016/s0730-725x(01)00391-5. PMID: 11551722. |
| **83** | **Sachdev 2003** | Sachdev RN, Champney GC, Lee H, Price RR, Pickens DR 3rd, Morgan VL, Stefansic JD, Melzer P, Ebner FF. Experimental model for functional magnetic resonance imaging of somatic sensory cortex in the unanesthetized rat. Neuroimage. 2003 Jul;19(3):742-50. doi: 10.1016/s1053-8119(03)00093-4. PMID: 12880803. |
| **84** | **Martin 2013** | Martin CJ, Kennerley AJ, Berwick J, Port M, Mayhew JE. Functional MRI in conscious rats using a chronically implanted surface coil. J Magn Reson Imaging. 2013 Sep;38(3):739-44. doi: 10.1002/jmri.23914. Epub 2013 May 15. PMID: 23677870; PMCID: PMC4500943. |
| **85** | **Ferenczi 2016** | Ferenczi EA, Zalocusky KA, Liston C, Grosenick L, Warden MR, Amatya D, Katovich K, Mehta H, Patenaude B, Ramakrishnan C, Kalanithi P, Etkin A, Knutson B, Glover GH, Deisseroth K. Prefrontal cortical regulation of brainwide circuit dynamics and reward-related behavior. Science. 2016 Jan 1;351(6268):aac9698. doi: 10.1126/science.aac9698. PMID: 26722001; PMCID: PMC4772156. |
| **86** | **Tang 2018** | Tang H, Kukral D, Li YW, Fronheiser M, Malone H, Pena A, Pieschl R, Sidik K, Tobon G, Chow PL, Bristow LJ, Hayes W, Luo F. Mapping the central effects of (±)-ketamine and traxoprodil using pharmacological magnetic resonance imaging in awake rats. J Psychopharmacol. 2018 Feb;32(2):146-155. doi: 10.1177/0269881117746901. Epub 2018 Jan 30. PMID: 29378483. |
| **87** | **Russo 2021** | Russo G, Helluy X, Behroozi M, Manahan-Vaughan D. Gradual Restraint Habituation for Awake Functional Magnetic Resonance Imaging Combined With a Sparse Imaging Paradigm Reduces Motion Artifacts and Stress Levels in Rodents. Front Neurosci. 2021 Dec 21;15:805679. doi: 10.3389/fnins.2021.805679. PMID: 34992520; PMCID: PMC8724036. |
| **88** | **Derksen 2021** | Derksen M, Rhemrev V, van der Veer M, Jolink L, Zuidinga B, Mulder T, Reneman L, Nederveen A, Feenstra M, Willuhn I, Denys D. Animal studies in clinical MRI scanners: A custom setup for combined fMRI and deep-brain stimulation in awake rats. J Neurosci Methods. 2021 Aug 1;360:109240. doi: 10.1016/j.jneumeth.2021.109240. Epub 2021 Jun 5. PMID: 34097929. |

*Shares authorship with Harris et al. 2015 (awake mouse fMRI, see **Table S2**).

^♦^ Shares authorship with Jonkers et al. 2014 (awake mouse fMRI, see **Table S2**).

Articles which exclusively re-use already published data are not included.

**Table S13.** **Summary of articles on rats in Group 1a (above the timeline).**

| **Study** | **M** | **F** | **Excluded** | **Strain** | **Anes.** | | | **Acclimation** | **Total** |
| --- | --- | --- | --- | --- | --- | --- | --- | --- | --- |
| **Lahti 1998** | 18 | 0 | 1 rat | SD | Chloral hydrate  (300 mg/kg; Sigma, St. Louis, MO) | | | None | 0 |
| **Lahti 1999*** | 6 |  | 0 |  |  |  |  |  |  |
| **Tenney 2003*** | 8 |  |  |  | Domitor and Antiseden | | |  |  |
| **Brevard 2003*** | 6 |  | 2/23 trial  8/2100 frames |  | Ketamine (10 mg/kg IP) and Domitor (medetomidine HCL; Pfizer, CT) (1 mg/kg IM) Antisedan (atipamizole HCL; Pfizer CT)  (5 mg/kg IM) to reverse the anesthesia | | |  |  |
| **Sicard 2003^♦^** | 15 | | 0 |  | 2% iso. | | |  |  |
| **Duong 2007^♦^** | 7 | |  |  |  |  |  |  |  |
| *Imaging begun 30 minutes after being placed in the holder and awakened.  **^♦^**Imaging began 1 hour after animal was placed in the restraint devise. | | | | | | | | | |
| **Study** | **M** | **F** | **Excluded** | **Strain** | **Anes.** | **Stress** | **Acclimation** | | **Total** |
| **Tenney 2004** | 10 | 0 | 0 | WAG/Rij | **Brevard 2003** | King (submitted) | Mock scanner | | 3-4 |
| **Febo 2004** | 26 |  | 2 scans | SD | 2% iso. | King (submitted) |  |  |  |
| **Ferris 2005** | 0 | 16 | Unk |  | 4% iso. | None |  |  | 4 |
| **King 2005** | 8 | 0 | 0 | SD | **Brevard 2003** | Measured | Using mock scanner and the real system | | 8 |
| **Febo 2005^a^** | 24 | 0 | 4 rats | SD | 2% iso. | None | Mock scanner | | 3 |
| **Febo 2005^b^** | 0 | 19 | 0 |  | ✔️ |  |  |  | 3-4 |
| **Ferris 2008** | 71 | 0 | 9 rats | LE | 2–3% iso. | **King 2005** |  |  | 4 |
| **Febo 2009** | 24 |  | 0 |  | ✔️ | None |  |  |  |
| **Caffrey 2010** | 0 | 20 | 3 rats |  | 2–4% iso. | **King 2005** |  |  |  |
| **Ferris 2010** | 55 | 0 | 9 rats |  | 2–3% iso. |  |  |  |  |
| **Kulkarni 2012*** | 58 |  | 6 rats | SD | ✔️ | None | **King 2005*** | | 8 |
| **Johnson 2013*** | 20 |  | 8 rats  or 12 sess. |  | 2–3% iso. | None | **King 2005*** | |  |
| **Reed 2013** | Unk |  | Unk | LE | 2–4% iso. | **King 2005** | Mock scanner (incremented) | | 5 |
| **Shah 2014** | 5 |  | 0 | SD | ✔️ |  | Mock scanner | | 4 |
| **Dumais 2017** | 22 | 25 | 9 sess. |  |  |  |  |  |  |
| **Madularu 2015** | 0 | 27 | 0 |  | 2–3% iso. |  |  |  | 5 |
| **Yee 2015** | 48 | 0 |  |  |  |  |  |  | 1 |
| **Ferris 2015** | 24 |  |  |  |  |  |  |  | 4-5 |
| **Kenkel 2016** | 22 |  |  |  |  |  |  |  |  |
| **Iriah 2019** | 25 |  | 15 rats |  |  |  |  |  |  |
| **Madularu 2016** | 0 | 36 | 0 |  | ✔️ |  |  |  | 5 |
| **Ferris 2017** | 48 | 0 | 11 rats |  | 2–3% iso. |  |  |  |  |
| **Madularu 2017^b^** | 36 |  | 10 rats | LE |  |  |  |  |  |
| **Total M/F** | **564** | **143** |  | | **Applied universally** |  | **Range (days)** | | **0-8**  **4 ± 2** |
| **Total (all)** | **729** | |  |  |  |  | **Mean ± SD (days)** | |  |

An unknown number of animals means that the dataset includes old and new data without the proportions being described. An unknown amount of data excluded means criteria were applied but amount of data that meeting these criteria was not specified. Zero excluded means no criteria for data exclusion was applied.

*Unclear if the acclimation protocol faithfully followed King et al. 2005.

Male (M), female (F), Sprague-Dawley (SD), anesthesia (Anes.), Long-Evans (LE), isoflurane (iso.), unknown (Unk), session (sess.), standard deviation (SD).

**Table S14.** **Summary of articles on rats in Group 1b (above the timeline).**

| **Study** | **M** | **F** | **Excluded** | **Strain** | **Anes.** | **Stress** | **Acclimation** | **Total** |
| --- | --- | --- | --- | --- | --- | --- | --- | --- |
| **King 2011** | 14 | 0 | Unk | SD | 2.5% Iso. | **King 2005** | Mock scanner | 3 |
| **Zhang 2010** | 8 |  | 0 | LE | Iso. |  | Mock scanner (incremented) | 8 |
| **Huang 2011** | 16 |  | Unk | FSL/FLR | ✔️ |  | **Lahti 1998**; **King 2005** |  |
| **Liang 2011** | 16 |  | 8 runs | LE | Iso. |  | Mock scanner (incremented) |  |
| **Liang 2012** | 8 |  | 17 runs |  |  |  |  |  |
| **Liang 2014** | 16 |  | Unk |  | 2% Iso. | Chronic stress via behavior test |  | 7 |
| **Crenshaw 2015** | 38 |  | 21% sess. | Wistar | 2.5% Iso. | **King 2005**; **Liang 2014** |  |  |
| **Liang 2015** | 7 |  | 0 | LE | 2% Iso. |  |  |  |
| **Ma 2017** | 25 |  |  |  | Iso. | **Liang 2014** |  |  |
| **Hamilton 2017** | 37 |  |  |  |  |  |  |  |
| **Ma 2018^b^** | 31 |  | Unk |  | 2% Iso. |  |  |  |
| **Ma 2018^c^** | 59 |  |  |  | 3% Iso. |  |  |  |
| **Ma 2018^a^** | 10 |  | 2% frames |  | 2-3% Iso. | **King 2005**; **Liang 2014**; **Ferenczi 2016** |  |  |
| **Pérez 2018** | 17 |  | Unk |  | 2-4% Iso. |  |  |  |
| **Dopfel 2019** | 87 |  |  |  |  | **King 2005** |  |  |
| **Liu 2019** | Unk |  | 4% frames |  |  | **Liang 2014**; **Dopfel 2019** |  |  |
| **Liu 2020**^*^ | Unk |  | 6% frames |  |  | **King 2005**; **Liang 2014**; **Dopfel 2019** |  |  |
| **Ma 2020** | 21 |  | Unk |  |  | Reviews |  |  |
| **Ma 2022** | 23 |  |  |  |  |  |  |  |
| **Tu 2021^a^** | 35 |  |  |  | Iso. |  |  |  |
| **Tu 2021^b^** | 9 |  |  |  |  |  |  |  |
| **Zhang 2022** | 35 |  |  |  |  |  |  |  |
| **Han 2022** | 17 | |  |  | 3% Iso. | **King 2005** |  |  |
| **Total (M/F)** | **512** | **0** |  | | **Applied universally** |  | **Range (days)** | **3-8** |
| **Total (all)** | **529** | |  |  |  |  | **Mean ± SD (days)** | **7 ± 1** |

An unknown number of animals means that the dataset includes old and new data without the proportions being described. An unknown amount of data excluded means criteria were applied but amount of data that meeting these criteria was not specified. Zero excluded means no criteria for data exclusion was applied.

^*^Open-source dataset shared.

Male (M), female (F), Sprague-Dawley (SD), anesthesia (Anes.), Long-Evans (LE), isoflurane (iso.), unknown (Unk), standard deviation (SD).

**Table S15.** **Summary of articles on rats in Groups 2-12 (below the timeline).**

| **Study** | **M** | **F** | **Exclude** | **Strain** | **Anes.** | **Stress** | **Restrain** | **Acclimation** | **Total** |
| --- | --- | --- | --- | --- | --- | --- | --- | --- | --- |
| **Tabuchi 2002*^☨^** | 10 | 0 | 2 or 5 rats | Wistar | Unk | None | 5-10 plastic screws. | Real scanner 7 days  (10 mins to 2 hrs.). | 7 |
| **Khubchandani^♦^* 2003** | 12 |  | Unk |  | None |  | Surgical implant with 4 pockets for acrylic screws. | In cages in scan room (noise) pre-surgery 3-4 days. Post-surgery, 2 days in scan room. Body then body & head restraint (few mins to 4-5 hrs.). | Unk |
| **Khubchandani^♦^* 2005** | 8 |  | 3 rats |  |  |  |  |  |  |
| **Tsurugizawa^☨^ 2010^a^** | 9 |  | Unk | SD | Iso. | **King 2005**, heart & respiration | Acrylic cement with 4 holes for glass-fiber bars. | Mock-scanner 3 days (30, 90 & 90 mins). Real-scanner  day 4 & 5. | 5 |
| **Tsurugizawa^☨^ 2010^b^** | 32 |  |  | Wistar |  |  |  |  |  |
| **Tsurugizawa^☨^ 2012** | 12 |  |  |  |  |  |  |  |  |
| **Uematsu^☨^ 2015** | 14 |  |  |  |  |  |  | **Tsurugizawa 2010^a^**, + daily handling 3 days. |  |
| **Kitamura^☨^ 2019** | 17 |  |  |  |  | **Tsurugizawa 2010^a^**, & no diarrhea | Resin affixed plastic pole  (7 x 30mm). | **Tsurugizawa 2010^a^** |  |
| **Skoubis 2006** | 44 | 0 | 7 rats | SD | Iso. | **King 2005** | **Group 1** | Mock-scanner 4 days (10, 30, 60 & 70 mins). | 4 |
| **Chin 2008 ^a^** | 15 |  | Unk |  |  |  |  | Mock-scanner 4 days (7, 30, 60 & 60 mins). |  |
| **Chin 2008^b^** | 62 |  | 6 rats |  |  |  |  |  |  |
| **Chin 2011** | 20 |  | Unk |  |  |  |  |  |  |
| **Upadhyay 2011** | 8 |  | 1 or 3 rats | LE |  | Respiration |  | Daily handling 5 days. Mock-scanner 2 days (10 & 30 mins), image day 5, 6, 8, 10 & 12. | 2 |
| **Baker 2012** | 15 |  | Unk | SD |  | None |  | Mock-scanner on nonconsecutive days over a 10-day span (a 10, a 30 and two 60 min sess.). | 4 |
| **Brydges 2013** | 32 |  | 10 or 8 rats | Lister Hooded |  | Heart rate |  | Daily handling 14-21 days. Mock-scanner day 1 & 3 (30 mins). | 2 |
| **Harris 2016** | 27 |  | 2 rats | BDNF^+/−^  (SD) |  | No group corticosterone differences |  | Daily handling 15 ± 5 days. Mock-scanner day 1 & 3 (30 mins). |  |
| **Becerra 2011^a^** | 24 | 0 | 5 rats | SD | Iso. | **King 2005**, & heart rate | **Group 1** | Mock-scanner for 3 days (30 mins). | 3 |
| **Becerra 2011^b^** | 15 |  | 2 rats |  |  | **King 2005** |  |  |  |
| **Chang* 2016** | 10 |  | <27 frames per rat |  | None | Respiration & corticosterone | Square plastic nut attached with Metabond & snuggle-sack. | Daily handling 3 days. Mock-scanner 8-10 sess. (30 mins/sess.) over 14 days, real-scanner 2 days. | 12 |
| **Chang* 2017** | 22 |  | 0, 3 or 8 rats |  |  | Respiration |  |  |  |
| **Wei* 2021** | 9 |  | 2 or 3 rats |  |  | None |  |  |  |
| **Becerra 2017** | 24 |  | 2 rats |  | Iso. |  | Cradle with ear bars and bite bar. | Mock-scanner 3 days (60 mins). | 3 |
| **Bishop 2019** | 24 |  | Unk |  |  |  |  |  |  |
| **Airaksinen 2012** | 5 | 0 | Unk | Wistar | Iso. | None | Stereotaxic apparatus & paralytics. | None | 0 |
| **Stenroos*^☨^ 2018** | 8 |  | 2/16 sess. |  |  | Respiration & heart rate, corticosterone | Head, shoulder & neck cradle with bite bar, limbs and tail taped, flexible plastic body cone. | Mock-scanner days 1-4 (15, 25, 35, 45 mins), day 8-11 (45 mins), image (25 mins) day 5 & 12. | 4 or 8 |
| **Paasonen*^☨^ 2018** | 8 |  | 2/16 sess. |  |  | Heart rate & corticosterone |  |  |  |
| **Paasonen*^☨^ 2020** | 9 |  | Unk |  |  | Respiration |  | Mock-scanner 4 days (15, 25, 35, & 45 mins). | 4 |
| **Dvořáková*^☨^ 2022** | 10 |  |  | SD |  |  |  |  |  |
| **Paasonen*^☨^ 2022** | 10 |  | 3.0 ± 2.4% of frames |  | None | Respiration & corticosterone | Head implant with two anchoring screws & body harness (limbs free but dangle). | Daily handling 7 days. Mock-scanner explore 3 days, head restraint by hand <30 sec. on day 4, day 5-9 full set-up (2, 5, 10, 15, & 20 mins), image day 10, 14, & 19. | 9 |
| **Peeters 2001** | 6 | | 0 | Wistar | Halo. | None | Stereotaxic apparatus & paralytics. | None | 0 |
| **Sachdev* 2003** | 3 | 2 | 2 of 9 sess. | LE | None | Tranquilized (diazepam) during imaging. | Three threaded nylon posts & body restraint device. | Daily handling 7 days. Mock-scanner >7 days gradual body restraint, then head-post surgery. Post-surgery, 9 days head & body restraint. | > 16 |
| **Martin 2013** | 0 | 4 | <10% of data &  1 rat for detached post | Lister Hooded |  | **King 2005** | Nylon cylinder (12 x 1 mm) & suspended body in a harness. | Mock-scanner 14 days gradual body restraint, then head-post surgery. Post-surgery, 7 days head & body restraint. Trained twice/day. | 21 |
| **Ferenczi***^☨^ **2016** | 0 | 24 | 43% of data | LE | Iso. | Respiration; **King 2005** | Head-post (3 screws) & ‘Snuggle’ system. | Mock-scanner 5-10 days (5 mins to full time in 5-10min increments). | 5-10 |
|  | 11 | 0 |  | SD |  |  |  |  |  |
| **Tang* 2018** | 37 | 0 | 5 rats | SD |  | **King 2005** | **Group 1** | Mock-scanner 5 days, day 1 (30 mins), days 2-5 (60 mins). | 5 |
| **Russo* 2021** | 6 |  | None | Lister Hooded |  | Respiration, weight & fecal corticosterone | **Stenroos 2018** | Daily handling 3 days. Mock-scanner, each step 3-days  (20, 40 & 60 mins), [1] Handling, [2] Body restraint (lights on), [3] Body restraint (dark), [4] Noise. [5] Head fixation. 2 days off between steps. | 12 |
| **Derksen 2021** | 16 |  | 2 rats (not trained) | Wistar |  | Dense corticosterone | Body restraint, bite bar & plastic head screw. | Mock-scanner 6 days (60 mins) versus untrained. | 6 or 0 |
| **Total (M/F)** | **588** | **30** |  | |  |  | | **Range (days)** | **0-21** |
| **Total (all)** | **624** | |  |  |  |  |  | **Mean ± SD (days)** | **6 ± 4** |

*Reward given during or after acclimation training.

^♦^Only animals that slept during noise exposure underwent surgery (number excluded unknown).

^☨^Strategy to reduce noise perception (e.g., earplugs) used.

Handling days are not counted as part of the total.

Anesthetized (Anes.), unknown (Unk), isoflurane (iso.), session (sess.), Long Evans (LE), Sprague Dawley (SD), halothane (halo.)

**Supplementary Figures**

1. Figure S1. Taxonomy of articles identified in our systematic review of the awake mouse fMRI literature (Figure 1 with color-coding).
2. Figure S2. Surgically implanted head-posts (Figure 2 with color-coding).
3. Figure S3. Experimental protocols for acclimating mice to awake fMRI.
4. Figure S4. Days of acclimation training and motion.
5. Figure S5. Quantitative measure of stress (Figure 3 with color-coding).
6. Figure S6. Acquisition hardware and sequence parameters.
7. Figure S7. Motion outcome measures 6-parameters.
8. Figure S8. Motion outcome measures FD (Figure 4 with color-coding).
9. Figure S9. Fraction of excluded data based on motion.
10. Figure S10. Taxonomy of articles identified in our systematic review of the awake rat fMRI literature (Figure 5 with color-coding).
11. Figure S11. Quantitative measure of stress from rats (Figure 7 with color-coding).
12. Figure S12. Motion outcome measures from rats (Figure 8 with color-coding).

**Figure S1.** **Taxonomy of articles identified in our systematic review of the awake mouse fMRI literature (Figure 1 with color-coding).**

**

**

**Figure S1.** Taxonomy of articles identified in our systematic review of the awake mouse fMRI literature. Articles are listed in chronological order. The number of articles published per year is indicated by a gray histogram background. Articles which share co-authors, and by extension an experimental approach, are linked by lines and color-coded (navy, salmon, marigold, and cyan). Groups which have contributed one article to the literature are shown in black and indicated by numbers with dashed outlines. The seminal article from each independent group is numbered.

**Figure S2.** **Surgically implanted head-posts (Figure 2 with color-coding).**

**
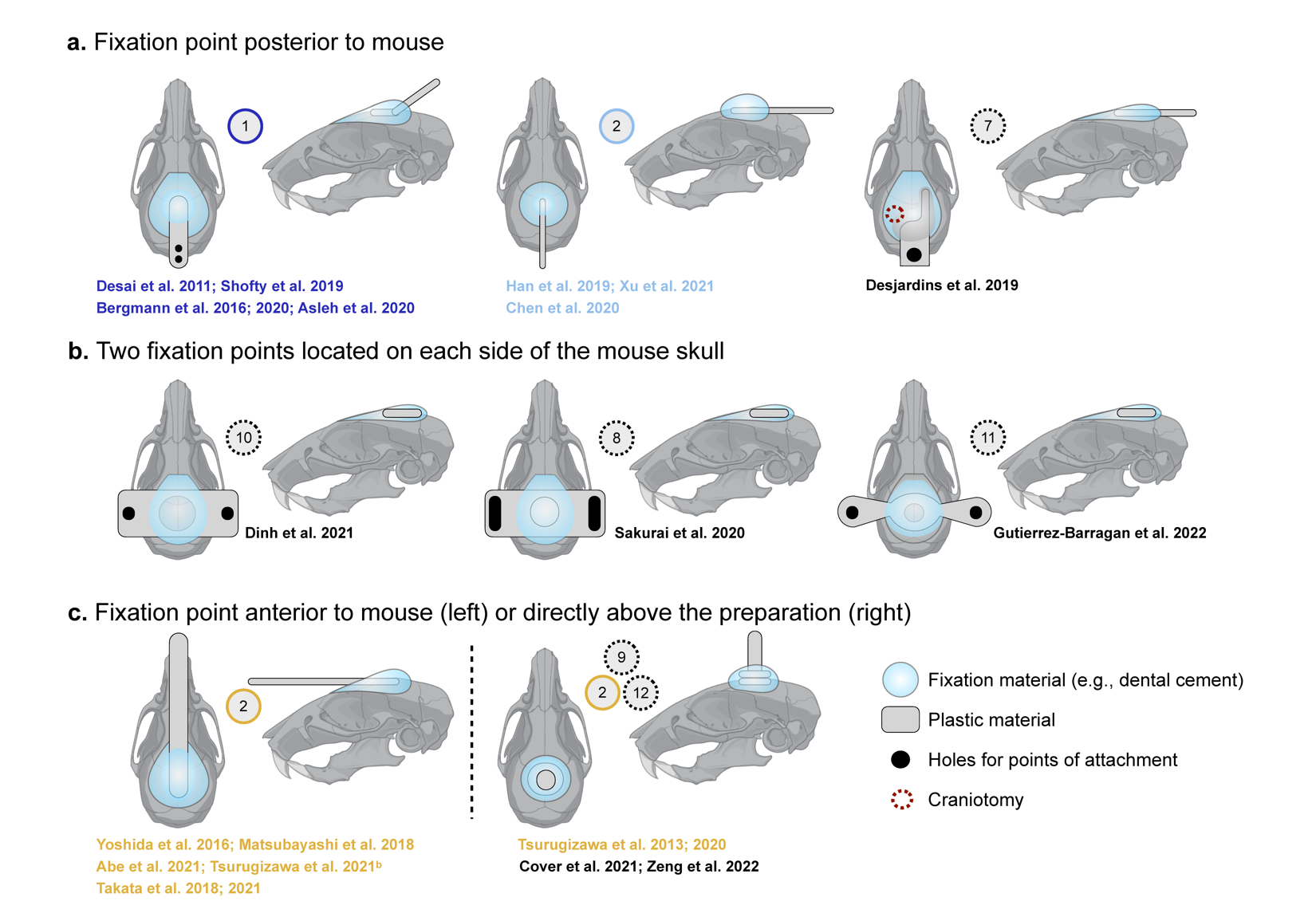
**

**Figure S2.** Surgically implanted head-posts. Head-posts used for immobilization during awake mouse fMRI fall into four categories based on the location of the fixation point(s): Posterior to the skull (**a.**), on both the right and left side of the skull (**b.**), anterior to the skull (**c**., left), and directly above the skull (**c.**, right). Cartoon drawings of the preparations are shown in two views (from above, and the left side). The articles that implement each preparation are listed below the images. Every preparation uses a fixation material (e.g. dental cement) to attach a plastic piece (custom-made) to the bone. Many groups also etch, scratch, or thin the bone to obtain a more robust attachment.

**Figure S3.** **Experimental protocols for acclimating mice to awake fMRI.**
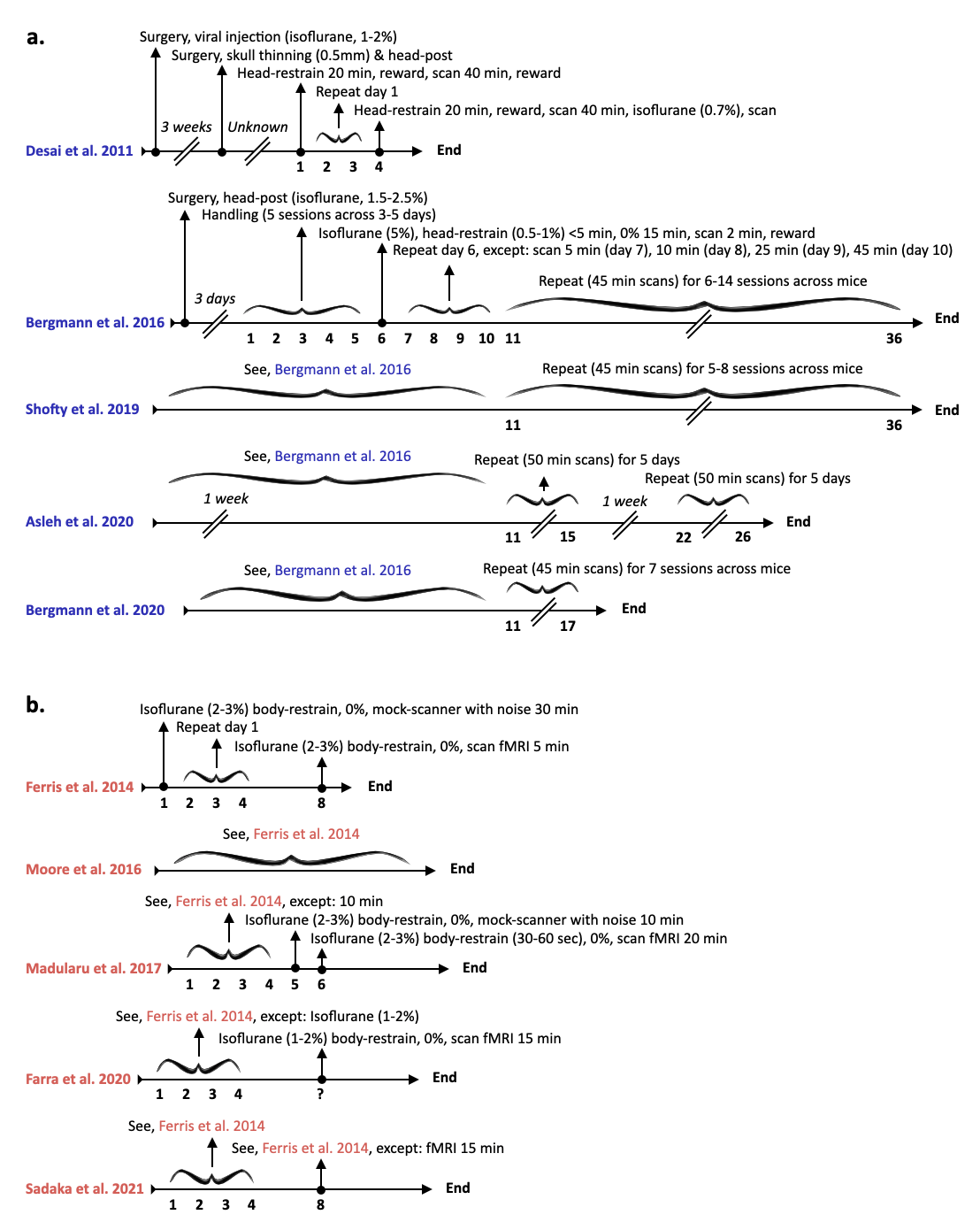


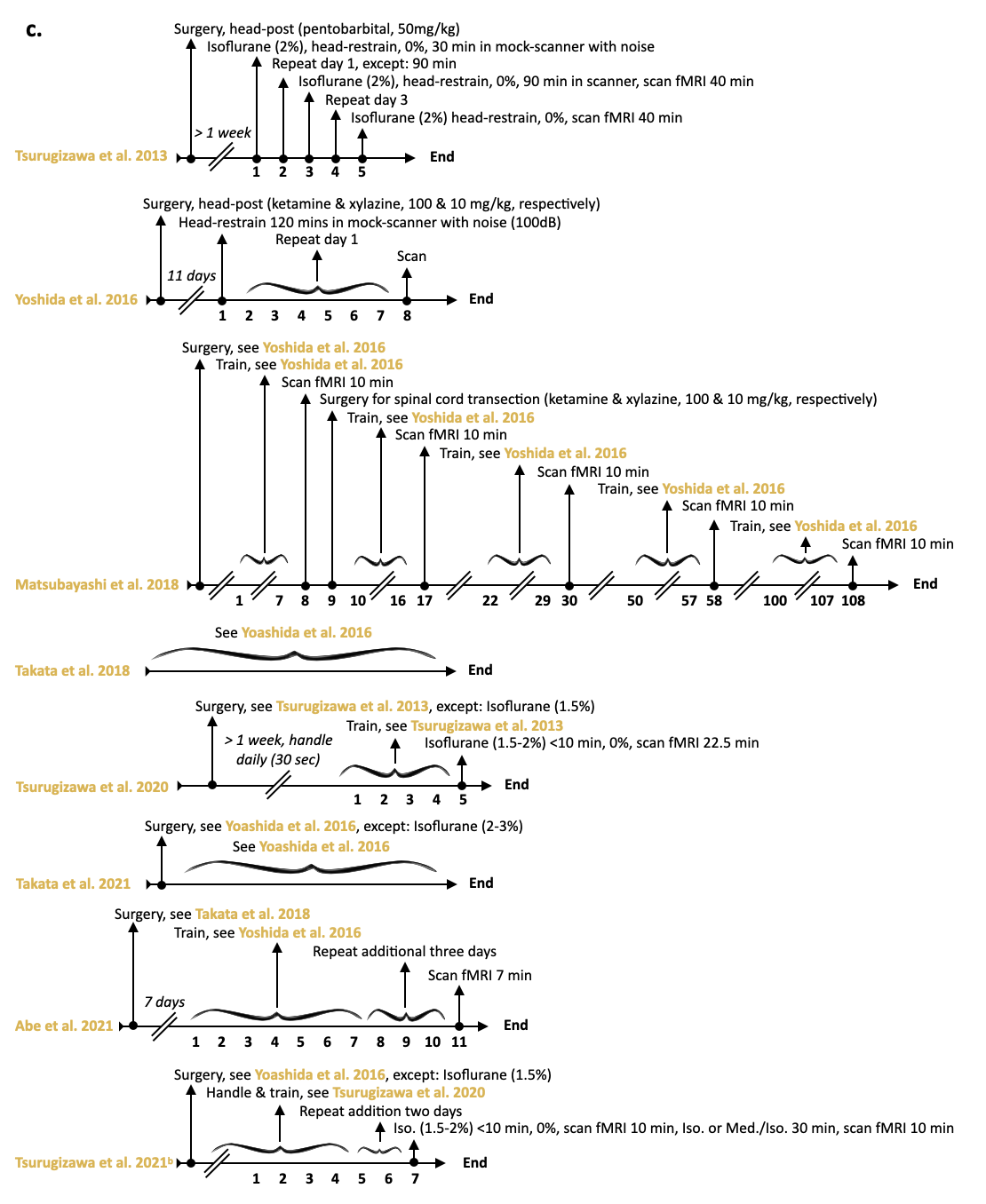


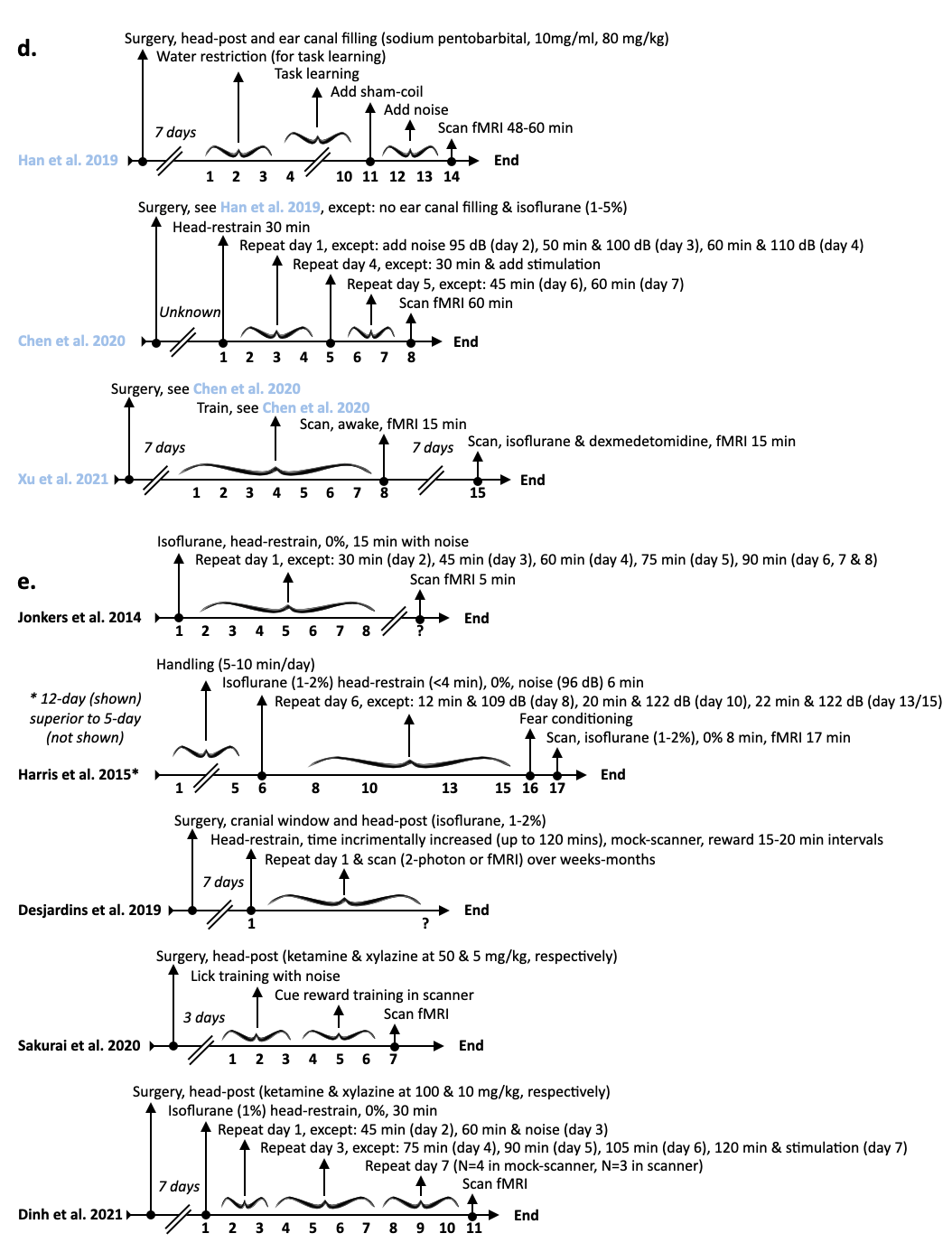


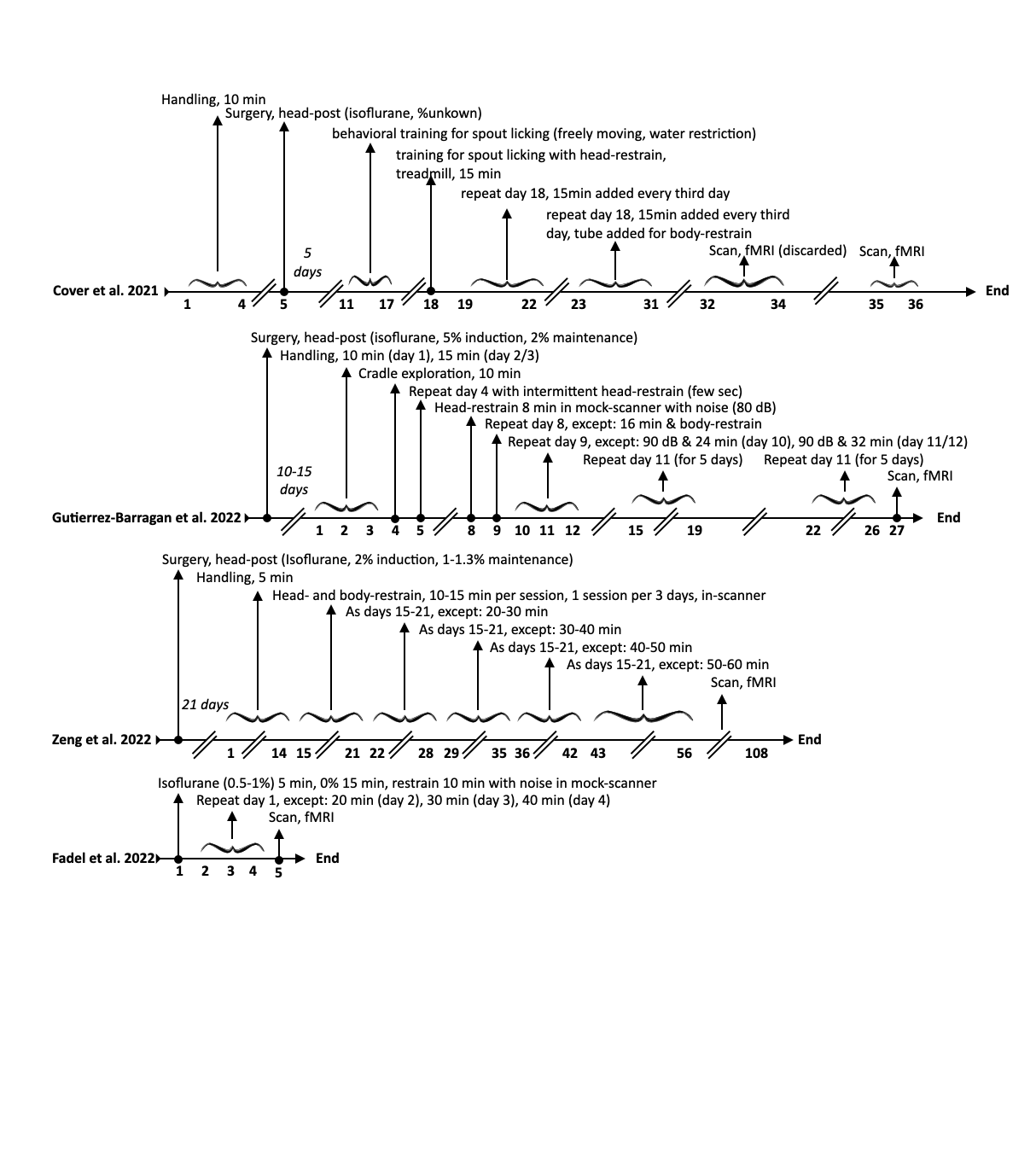


**Figure S3.** Each acclimation protocol is shown as a timeline. Day 1 is the first day of acclimation. Head-post surgery (if performed) and recovery occur before day 1. Handling or restraint are considered part of acclimation. Protocols are grouped by common authorship (**a.**, **b.**, **c.**, and **d.**). Protocols from groups with one publication are shown in (**e.**). When anesthesia is utilized for surgery or to mount the animal in a restraint system the type and dose are noted as well as the time allotted for the effects to wane during acclimation and imaging (if this information is provided). For example, (**a.**) Bergmann et al. 2016, day 6: “Isoflurane (5%), head-restrain (0.5%-1%) <5min, 0% 15 min, scan 2min” – anesthesia is induced with 5% isoflurane which is then reduce to 0.5-1% while the animal is mounted into the restraint system. This procedure takes <5 minutes. Isoflurane is then discontinued for 15 minutes before data are collected (for 2 minutes). If a mock-scanner or the real system is described, this is noted. Medetomidine (Med.), isoflurane (Iso.).

**
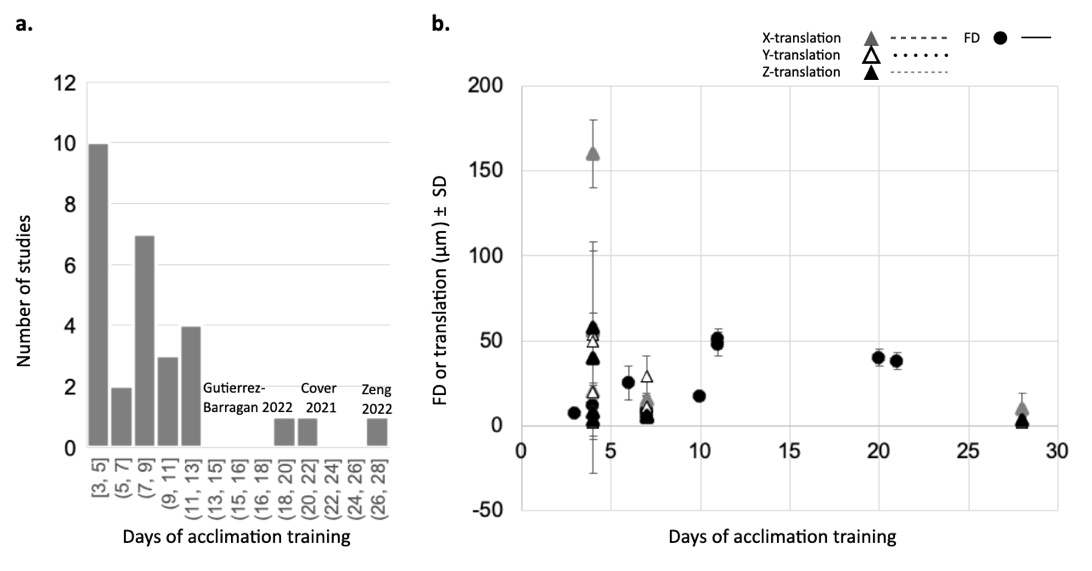
Figure S4.** **Days of acclimation training and motion.**

**Figure S4.** Histogram showing the number of days studies used in their acclimation protocol (**a.**) (**Table 2**). Plot of average motion, either FD (circles) or translation (triangles), against the number of days in the study acclimation protocol (**b.**). Linear trends are indicated but are for display purposes only due to insufficient data (and the likelihood of a non-linear relationship). MATLAB, *corr.*

**Figure S5.** **Quantitative measure of stress (Figure 3 with color-coding).**


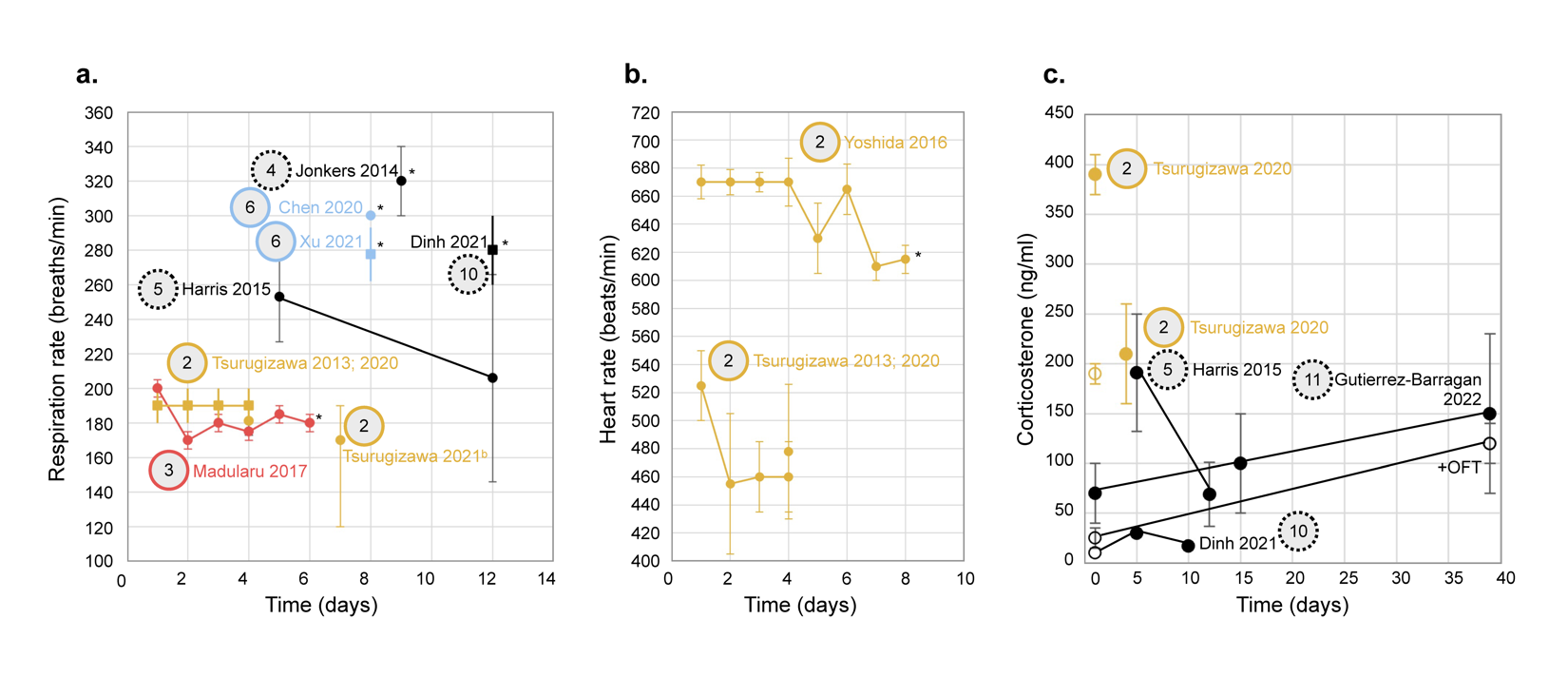


**Figure S5.** Quantitative measures of stress. Respiration rate (**a.**), heart rate (**b.**), and corticosterone (**c.**) are the three most common quantitative measures of stress used to evaluate acclimation (**Table S6**). All data (**a.**, **b.**, & **c.**) are collected from adult male C57BL/6 J mice except a subset of heart rate measurements obtained in the 2020 Tsurugizawa et al. study (Tsurugizawa et al. 2020) which included 15q dup animals (a model of 15q11–13 duplication (15q dup), a copy number variation model of autism on a C57BL/6 J background) (**Table 1**). Notably, 15q dup mice show no differences in heart rate compared to wild-type (WT). Moreover, corticosterone measures in (Tsurugizawa et al. 2020) were only acquired from WT animals. Thus, sex and strain are not driving inter-study differences. On all plots, study (1st author and date) is indicated and color-coded as above. An asterisk indicates physiological data collected during fMRI acquisition, or on the “imaging day” if in-scanner training was performed (**Figure S3**). In all plots, some values are taken from the text while others are inferred from figures. Data from the same study are linked by lines. Reported error (standard deviation, or standard error, see **Table 1**), is indicated by error bars (with caps). (**a.**) Studies which reported a range, (Tsurugizawa et al. 2020; Dinh et al. 2021), are plotted as squares with solid-colored lines (no caps) indicating the range and the data point indicating the middle of the range. (**c.**) Studies reporting measures of corticosterone. Dinh et al. report longitudinal data (*n* = 2) acquired before training (day 0) as well as after 5 and 10 d of acclimation. Data are acquired from the same mice (linked with a solid line). Control data (day 0) are plotted using an open circle. Tsurugizawa et al. report data from three groups: *N* = 5 (without training, day 0, open circle), *n* = 4 (day 1, solid circle), and *n* = 5 (day 4, solid circle). It is unclear whether data are acquired from the same animals (no lines). Finally, Gutierrez-Barragan et al. collect data from co-housed control (*n* = 7) and acclimated (*n* = 7) groups. Corticosterone measures are acquired after handling (prior to mock-scanner training, **Figure S3**) in the acclimated group, as a baseline (day 0, solid circle) alongside measures from a control group (no handling, day 0, open circle). Handling is not considered part of training in this plot. Following 24 d of mock-scanner training (solid circle, solid line) or regular housing (open circle, dotted line) corticosterone levels are re-measured. Notably, control mice undergo an open field test (OFT) prior to this second corticosterone measurement.

**Figure S6.** **Acquisition hardware and sequence parameters.**


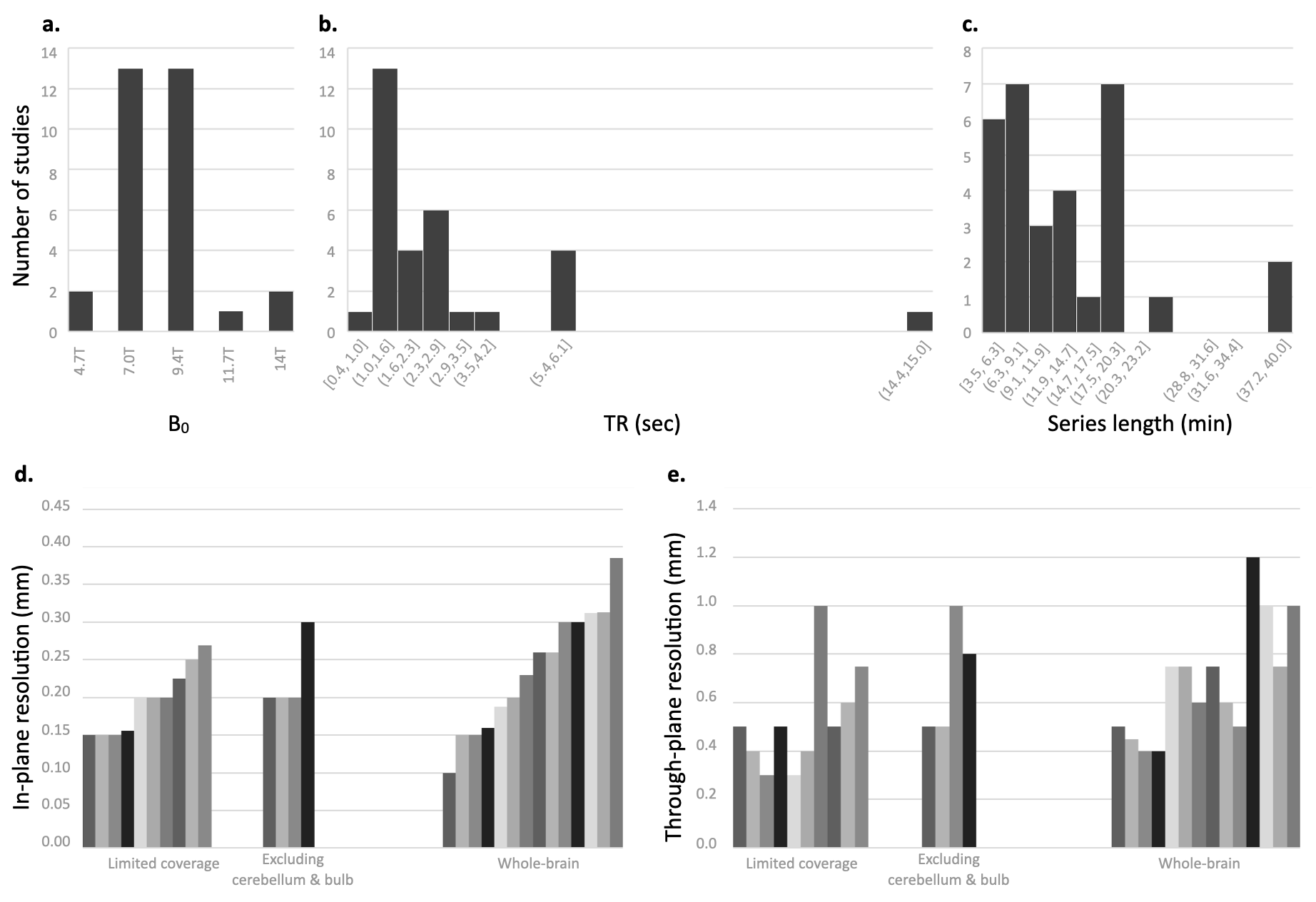


**Figure S6.** Histograms of scanner field strength (B_0_) (**a.**), temporal resolution (TR) (**b.**), and time series length (**c.**). Spatial resolution including in-plane (**d.**) and through-plane (**e.**) resolution. In (**d.** & **e.**) each bar corresponds to an acquisition (some studies include more than one, e.g., Chen et al. (Chen et al. 2020)). Acquisitions are separated into three ‘coverage groups’, from left-to-right, those that have limited brain coverage (focus on a specific ROI), excluded the cerebellum & olfactory bulb, and included the whole brain. For each coverage group, acquisitions are ordered from lowest-to-highest in-plane resolution (**d.**). This ordering is preserved in (**e.**) to maintain correspondence across (**d.** & **e.**). ROI = region of interest.

**Figure S7.** **Motion outcome measures 6-parameters.**

**
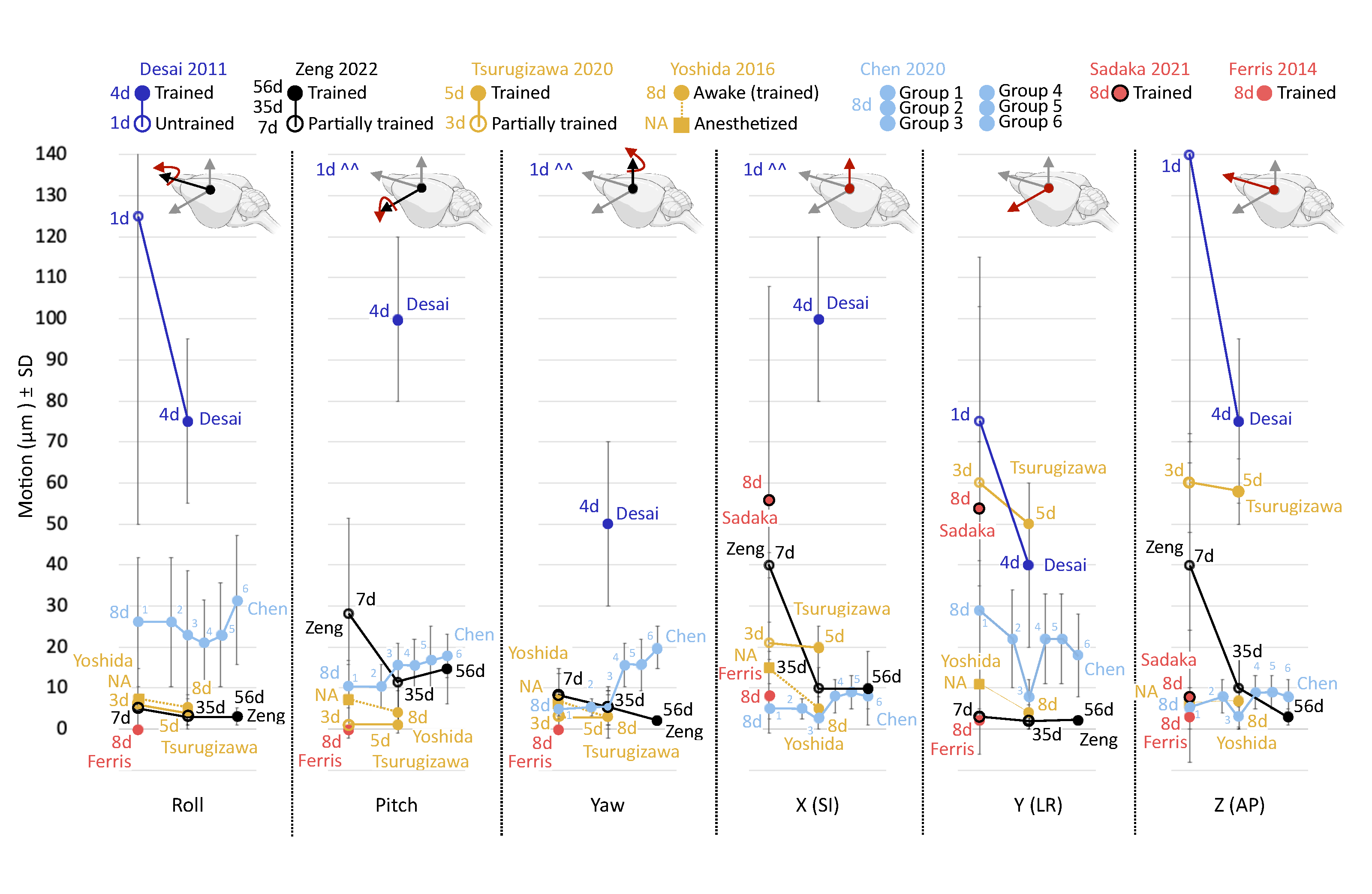
**

**Figure S7.** Studies that report motion using either a six-parameter estimate (above) or FD (**Figure 4** or **Figure S8**). Rotations are reported in μm assuming the mouse brain has a diameter of 6 mm (Yoshida et al. 2016). Data from the same group of mice are linked by lines (solid, dashed, or dotted) based on group (see legend). The six-parameters are separated by vertical black dotted lines. Only one study reports both 6-motion parameter estimates (above) as well as FD (Chen et al. 2020) (**Figure 4** or **Figure S8**). Superior–inferior (SI), left–right (LR), and anterior–posterior (AP).

**Figure S8.** **Motion outcome measures FD (Figure 4 with color-coding).**

**
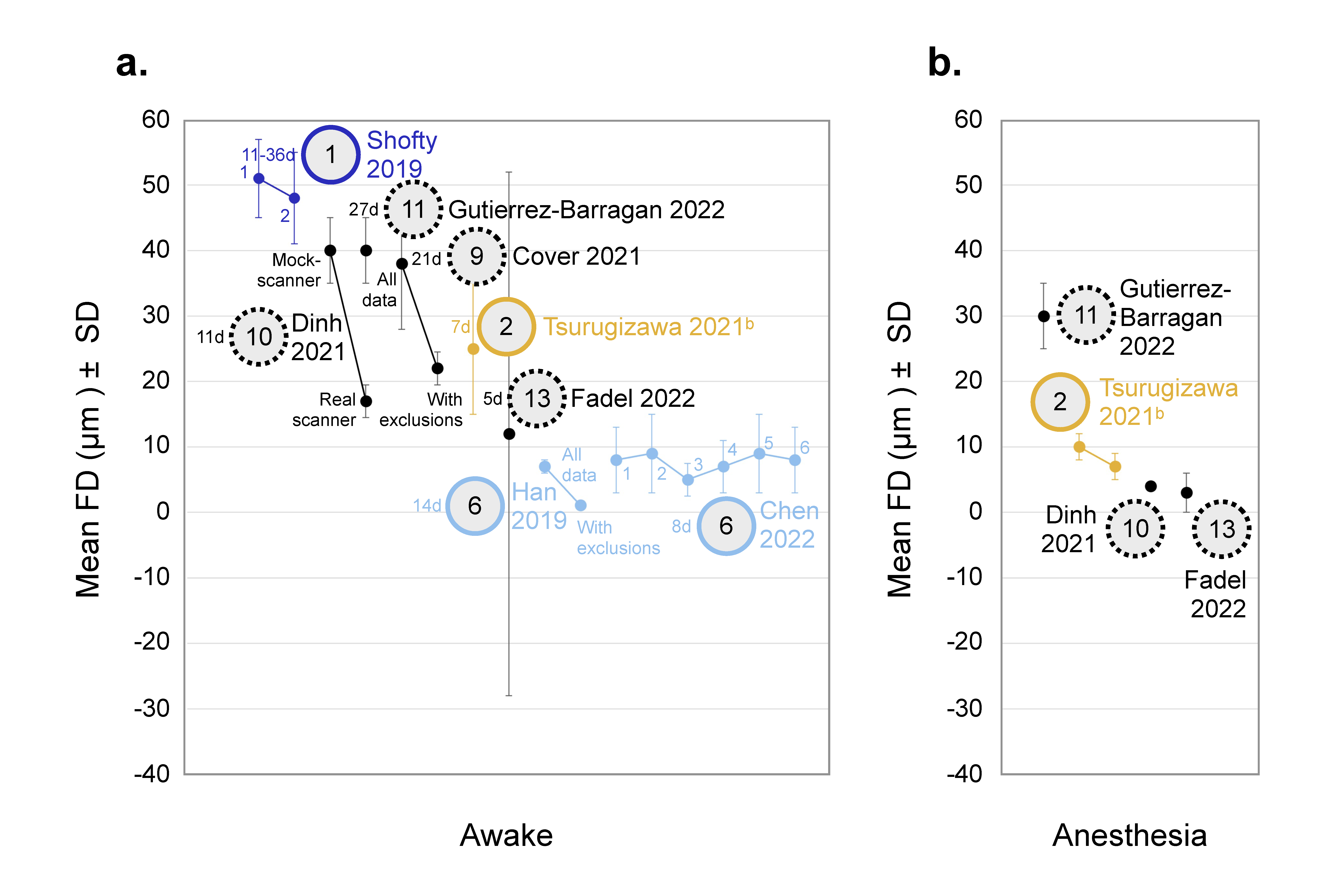
**

**Figure S8.** Motion outcome measures. Studies that report motion using either a six-parameter estimate (**Figure S7**) or FD (**a.** & **b.**). In (**a.**), data from awake mice are plotted. In (**b.**), data from anesthetized mice are plotted. Data from the same study (different groups) are included.

**
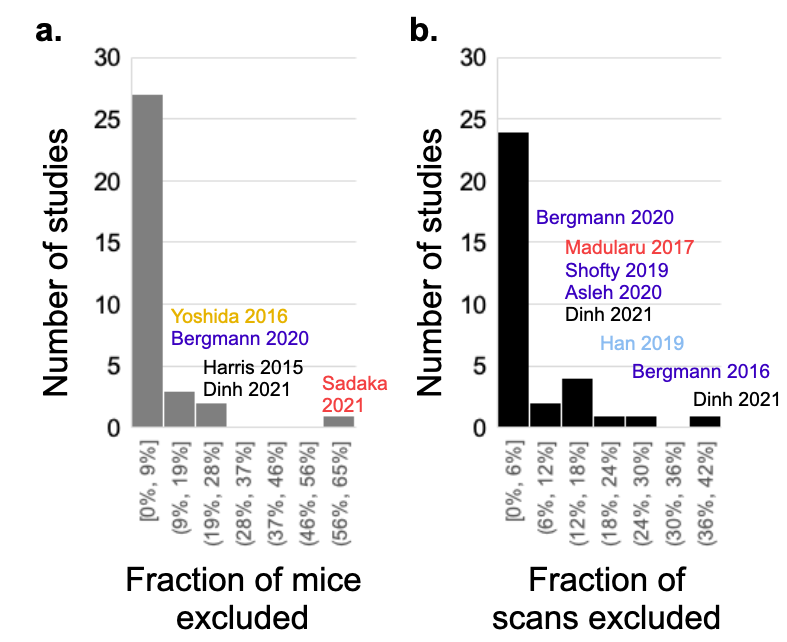
Figure S9.** **Fraction of excluded data based on motion.**

**Figure S9.** Histograms showing how many studies exclude various fractions of data either by number of mice (**a.**) or scans (**b.**). Studies plotted are in **Tables S9** & **S10**. Studies that exclude fractions of data that are greater than most, > 9% of mice (**a.**) or > 6% of scans, are listed on the histograms above their corresponding bins.

**Figure 10.** **Taxonomy of articles identified in our systematic review of the awake rat fMRI literature (Figure 5 with color-coding).**

**

**

**Figure S10.** Taxonomy of articles identified in our systematic review of the awake rat fMRI literature. Summary of articles which collect fMRI data from awake rats (as **Figure 1** does for mice). Articles are listed in chronological order. The number of articles on rats published per year is indicated by a gray histogram background. For reference, the number of articles on mice published per year is indicated by a yellow histogram background. Articles which share co-authors, and by extension an experimental approach, are linked by lines and color-coded. Groups which have contributed one article to the literature are indicated by numbers with dashed outlines in black. The seminal article from each independent group is numbered. The total number of articles in subgroups 1b, 1a, and all others are noted on the far right (24, 29, and 36, respectively). Groups (labeled here as: 1, 3, and 5) have also published awake mouse data (see legend).

**Figure 11.** **Quantitative measure of stress from rats (Figure 7 with color-coding).**

**
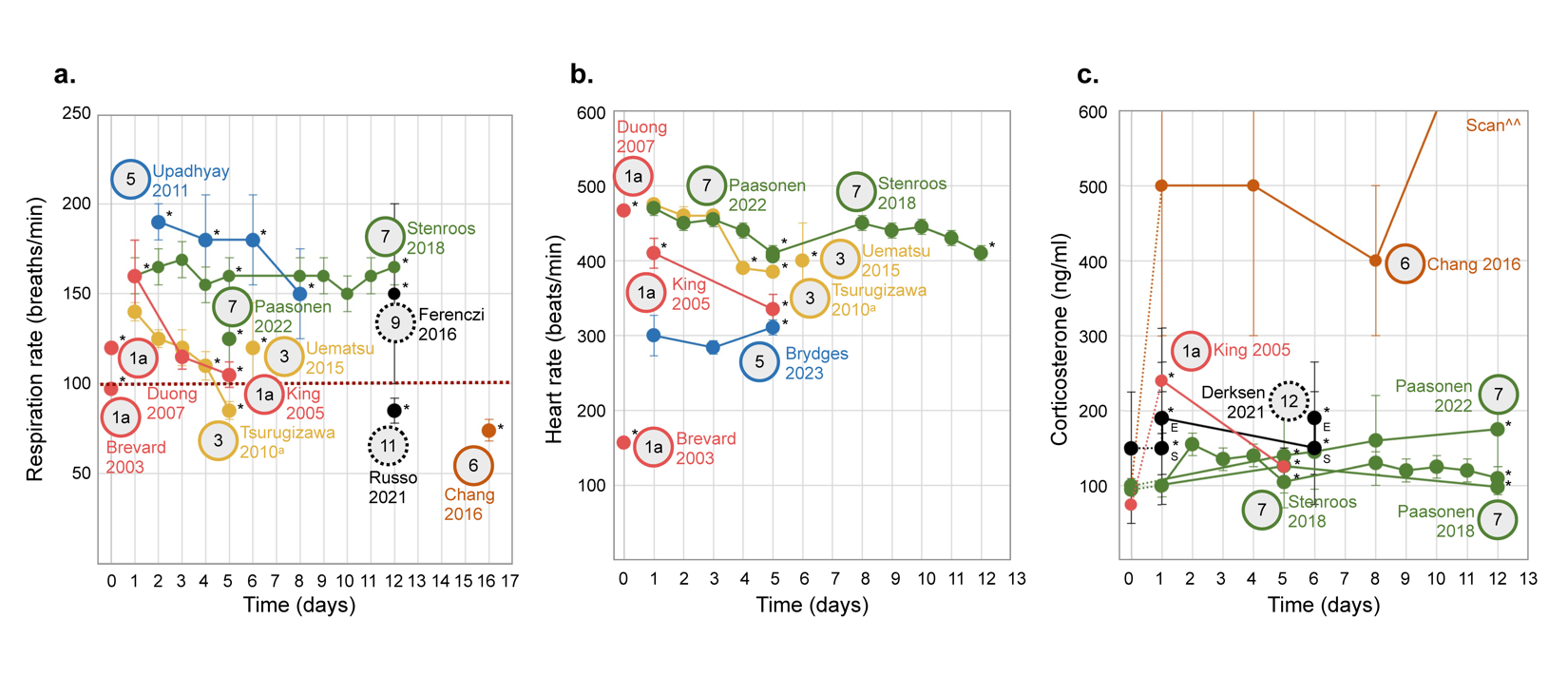
**

**Figure S11.** Quantitative measure of stress from rats. Respiration rate (a), heart rate (b), and corticosterone (c) for awake rat fMRI studies. Data from the same study are linked by lines. Dotted lines link data collected at baseline (time, 0 d) and data collected during acclimation training or imaging. Data points indicated by an asterisk are collected on imaging days (real-scanner). In (a), a horizontal red dotted line (at 100 bpm) is shown because many studies only proceed with fMRI data acquisition after this rate has been exceeded (as an indication that the effects of anesthesia have worn off). Data points/lines are color-coded to match numbered groups (**Figure S10**). Strains break down as: SD (Brevard et al. 2003; King et al. 2005; Duong et al. 2007; Tsurugizawa et al. 2010a; Chang et al. 2016; Paasonen et al. 2022), Wistar (Uematsu et al. 2015; Paasonen et al. 2018; Stenroos et al. 2018; Derksen et al. 2021), Lister hooded (Brydges et al. 2013; Russo et al. 2021), LE (Upadhyay et al. 2011), and unknown (Ferenczi et al. 2016). Sex is not specified by two studies (Duong 2007; Ferenczi et al. 2016) but is otherwise male. Two studies do not use anesthesia (Chang et al. 2016; Paasonen et al. 2022). In (c), two data points appear on days 1 and 6 (Derksen et al. 2021). These are collected at the “start” (S) and “end” (E) of the imaging session (as indicated on the plot).

**Figure 12.** **Motion outcome measure from rats (Figure 8 with color-coding).**

**
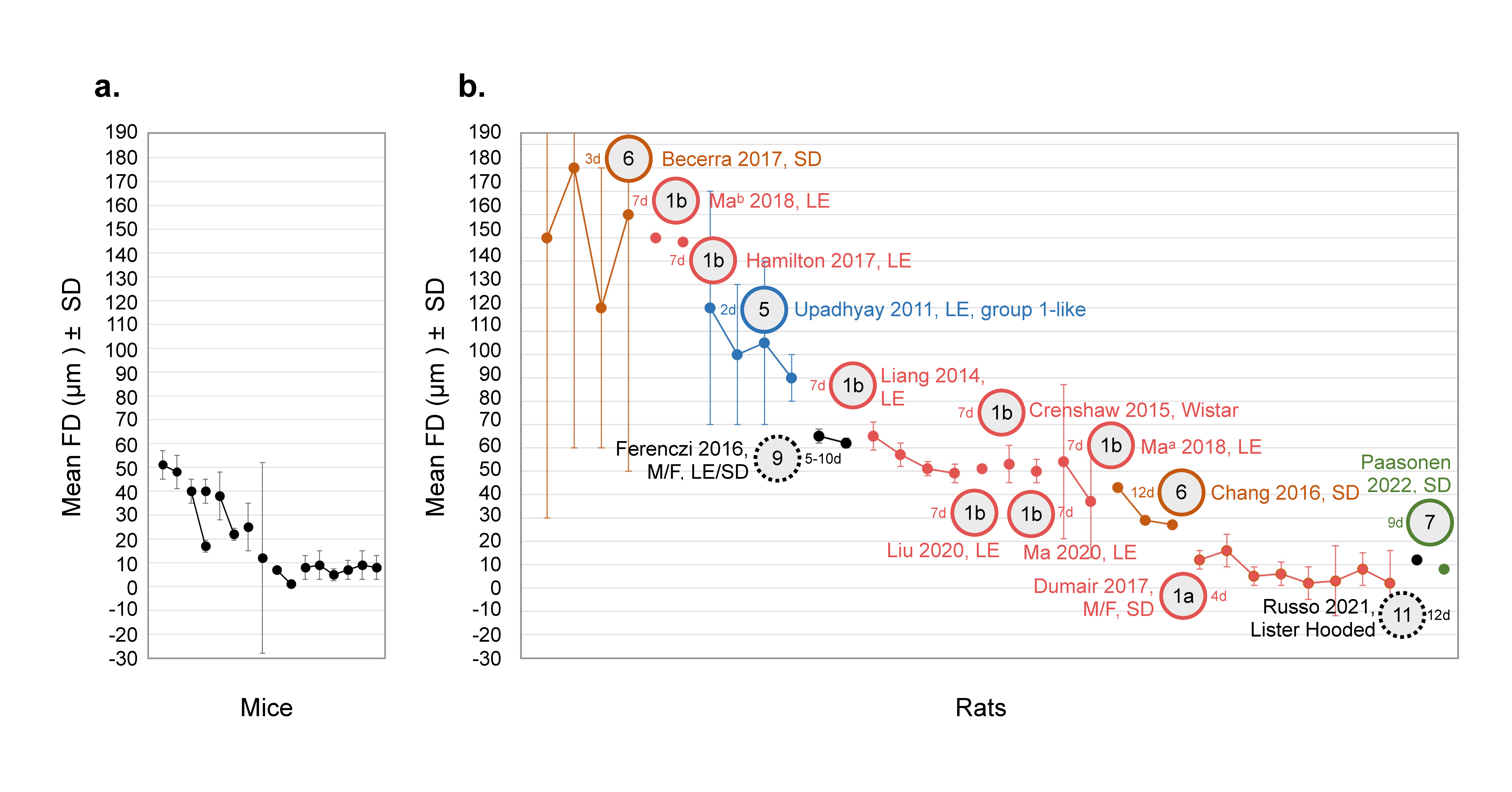
**

**Figure S12.** Motion outcome measures from rats. Studies in mice (**a.**) and rats (**b.**) that report mean framewise displacement (FD). Data in (**a.**), from mice, are reproduced from **Figure 4** for reference. Data in (**b.**), are color-coded to match numbered groups (**Figure S10**). Data from the same study are linked by lines. In (**b.**), subgroups 1a and 1b, as well as group 1-like are noted alongside rat strain and the length of the acclimation protocol. Two studies, by Ferenczi et al. (2016) and Dumais et al. (2017) use both male and female rats - Indicated as M/F - (all others use only male rats). Three studies, by Chang et al. (2016), Ferenczi et al. (2016) and Paasonen et al. (2022), use a surgical implant to aid in reducing head motion. Chang et al. (2016) and Paasonen et al. (2022) are the only two that do not use anesthesia to mount and unmount rats from the immobilization set-ups.
